# Earthquake-triggered cascading hazards under Arctic amplification

**DOI:** 10.1073/pnas.2617210123

**Published:** 2026-09-08

**Authors:** Guilherme W. S. de Melo, Reginald L. Hermanns, Jacob M. Bendle, Ingo Grevemeyer, Sylvain Fiolleau, Simone Cesca, Aderson F. do Nascimento, Lars Ottemöller, Gökhan Aslan, Quentin Brissaud, Volker Oye, Heidrun Kopp

**Affiliations:** ^a^ Division of Dynamics of the Seafloor-Marine Geodynamics, Division of Dynamics of the Seafloor-Marine Geodynamics, GEOMAR Helmholtz Centre for Ocean Research Kiel, Kiel 24148, Germany; ^b^ Geological Survey of Norway, Trondheim 7040, Norway; ^c^Helmholtz Centre for Geosciences Potsdam, Potsdam 14467, Germany; ^d^ Departamento de Geofisica, Federal University of Rio Grande do Norte, Natal 59078-900, Brazil; ^e^ Department of Earth Science, University of Bergen, Bergen 5020, Norway; ^f^ Norwegian Seismic Array, Kjeller 2027, Norway; ^g^ Faculty of Mathematics and Natural Sciences, Institute of Geoscience, Kiel University, Kiel 24118, Germany

**Keywords:** earthquake-triggered rock avalanche, Arctic amplification, permafrost degradation, tectonic–cryosphere interactions

## Abstract

Arctic warming is reshaping permafrost and glacierized terrains, potentially changing their natural hazards in response to seismic shaking. On 10 March 2025, a Mw 6.5 oceanic transform fault earthquake near Jan Mayen Island in the Arctic Ocean triggered a large rock avalanche that buried part of the Kjerulf Glacier, generating infrasound signals and glacier calving. Using seismic, Global Navigation Satellite System data, infrasound records, satellite imagery, and long-term climatological records, we reconstruct a cascade of processes linking earthquake rupture to rock slope failure and ice calving. The rock avalanche occurred within minutes of the earthquake accompanied by a transient reduction in near-surface seismic velocity. Satellite observations further reveal multiple precursor slope failures during the years preceding the earthquake, indicating progressive instability of the slope. Long-term warming and permafrost degradation, likely contributed to reduce the slope stability, increasing susceptibility to the seismic ground shaking trigger. Our findings suggest that Arctic warming may progressively increase the susceptibility of slopes to earthquake-triggered failure in the Arctic, highlighting the emerging interactions between tectonic hazards, cryosphere instability, and climate change.

## Significance

Arctic warming is rapidly changing the permafrost and glacierized places, yet its influence on earthquake-related hazards remains poorly understood. We investigate a major 2025 Arctic earthquake that triggered a large rock avalanche and glacier burial on Jan Mayen Island using seismic, satellite, infrasound, Global Navigation Satellite System, and climate observations. Our results show how strong seismic shaking can produce cascading impacts across the lithosphere and cryosphere, while long-term climate warming may progressively increase the slope susceptibility to failure. By integrating multiple geophysical monitoring techniques, this study highlights the emerging interactions between climate change and natural hazards in rapidly changing polar environments.

**Fig. 1.**
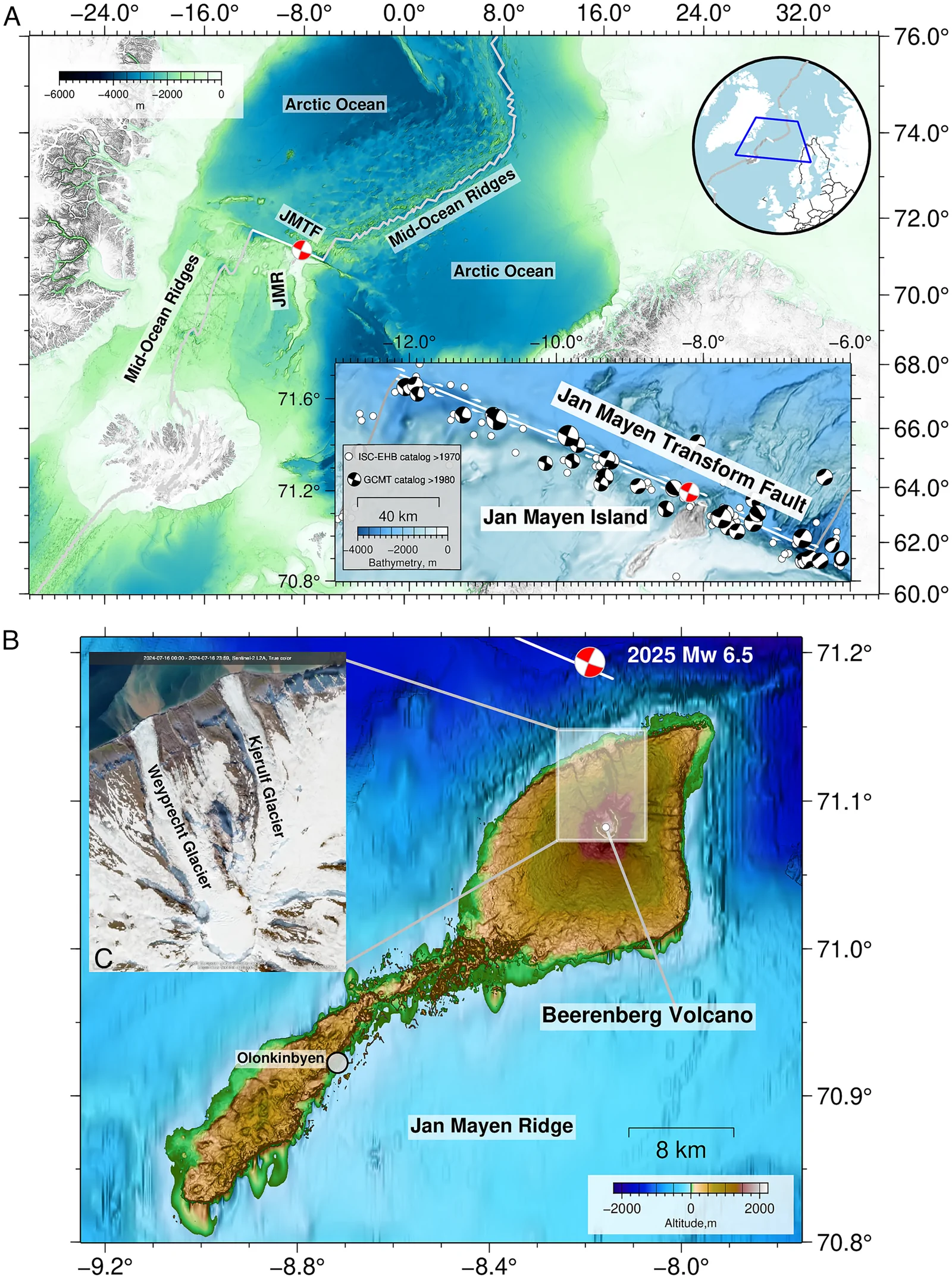
Tectonic setting of the Jan Mayen Transform Fault (JMTF) and location of the 10 March 2025 Mw 6.5 earthquake. (*A*) Bathymetric map of the Arctic Ocean. Gray traces indicate the Mid-Ocean Ridges, and white traces refer to the oceanic transform faults (1). The location of the Jan Mayen Ridge (JMR) and the JMTF are indicated in the map. Red focal mechanism indicates the epicenter location of the 10 March 2025 Mw 6.5 strike-slip earthquake, rupturing close to JMI presented on bathymetric map on right bottom. Black focal mechanisms refer to events from the Global Centroid Moment Tensor catalog (GCMT) (2, 3) since 1980, and white circles refer to earthquakes occurring since 1970 cataloged by the International Seismological Centre (ISC). (*B*) Enlarged map of the JMI showing in more detail the island with the location of the Beerenberg stratovolcano, as well as the location of the Weyprecht and Kjerulf Glaciers shown on 10m resolution Sentinel-2 satellite image (4) in *C*).

On 10 March 2025, an oceanic strike-slip earthquake with moment magnitude (Mw) of 6.5 occurred in the area west of Jan Mayen Island (JMI) in the Arctic Ocean. The island lies next to the Jan Mayen oceanic Transform Fault (JMTF; Fig. 1 *A* and *B*), which offsets the Mid-Atlantic Ridge and the Eurasian and North American plates, slipping at 14 mm/y (5). The event was the largest magnitude earthquake recorded in the region (~7 km away NW of the JMI) over the last 100 years, surpassing the previous 1979 Mw 5.8, 1988 Mw 6.0, 1997 Mw 5.6, 2000 Mw 6.0, and 2004 Mw 5.9 events reported by the United States Geological Survey (USGS) (6). The magnitude of the 2025 event suggests that its rupture extended ~40 km along strike (7), affecting the northern sector of JMI. Local aftershock distribution, Global Navigation Satellite System (GNSS) displacement data, and near field seismological stations provided critical constraints on earthquake rupture behavior. Together, these observations offer a rare opportunity to investigate the impact of a strong seafloor earthquake on adjacent landforms and associated environmental consequences.

JMI hosts Beerenberg volcano (Fig. 1*B*), the northernmost above sea level active volcano on Earth. This 2,277 m tall stratovolcano (8), formed by seafloor volcanic activity within the last 700,000 years (9), and erupted last in 1985 (10). Owing to its Arctic location, the Beerenberg cone-shaped hills are glaciated and snow-covered for most of the year, with the summit always covered. Its two largest outlet glaciers, Weyprecht and Kjerulf (Fig. 1*C*) (11), with their ice cliff along the shoreline, located only 6 to 7 km from the USGS epicenter. The automated ground failure model of the USGS predicted a small probability of seismic-triggered landslides at northwestern rock flanks of the Kjerulf (12). In Arctic environments, the climate warming and permafrost degradation are increasingly recognized as key controls on rock slope stability, progressively weakening the rocks and preconditioning slopes to failure (13, 14).

Previous studies suggested that shaking of earthquakes with Mw > 6.5 can induce rock avalanches up to 50 to 90 km away from an epicenter, and 20 to 50 km from a ruptured fault (15), particularly in steep volcanic terrain with ice-covered slopes occurring within ~10 km of the epicenter (16), consistent with the USGS prediction for the Jan Mayen earthquake. Rock avalanches in such settings can be detected through geophysical and satellite signatures, including infrasound (17, 18), changes in local seismic velocities (19, 20), and ground deformation from interferometric synthetic aperture radar (21, 22). With such data available, Jan Mayen provides a unique opportunity for integrating diverse geophysical observations, yet no earthquake-related rock avalanches have ever been documented on the JMI.

Here, we use local seismic and GNSS data, together with high resolution (50 cm) panchromatic satellite imagery, to demonstrate that the Mw 6.5 Jan Mayen earthquake generated >1500 aftershocks, ruptured ~40 km eastward along the JMTF, displaced the JMI surface by up to 2.4 cm, caused local seismic velocity changes, and triggered a rock avalanche on Kjerulf Glacier located 7 km away of the epicenter. The rock avalanche with an estimated source area volume of $990,000\pm 180,000\;m^{3}$ occurred within ~3 min after the mainshock as evidenced by infrasound signals generated by the rock mass movement. The resulting debris sheet covered 30 to 50% of the glacial surface, with implications for long-term permafrost degradation and glacier mass balance. Additionally, satellite radar imagery taken 4 h after the earthquake revealed evidence that an ice calving event occurred at shoreline of the Weyprecht Glacier, later confirmed by optical imagery. This study highlights the importance of multidisciplinary geoscientific approaches not only for characterizing earthquake rupture, but also for understanding emerging earthquake–climate–landscape interactions in Arctic environments.

## Seismicity and Ground Displacement

The USGS located the 10 March 2025 Mw 6.5 strike-slip earthquake northwest of the island, with Kjerulf Glacier shoreline only 5.5 km from the epicenter. We reanalyzed the source parameters using polarization analysis (23, 24) of the first-arriving Pg phase from the local seismic stations JNE, JNW, and JMI (*Methods* and *SI Appendix*, *Supplementary Text S1*). Iterative relocation yielded a best-fitting epicenter at 71.1935 N, 8.1905° W and a source depth of 18 km (*SI Appendix*, Fig. S1) with a horizontal uncertainty of 0.9 km, originating at 02:33:15.8 UTC. Our epicenter locates less than 1 km away from the USGS solution (*SI Appendix*, Table S1). Regional waveform inversion (25) (*Methods* and *SI Appendix*, *Supplementary Text S1* and Table S2 and Figs. S2–S4) using eight broadband land stations provided a consistent focal depth of 18 to 19 km after water-layer correction (26, 27), supporting the polarization-based estimate.

**Fig. 2.**
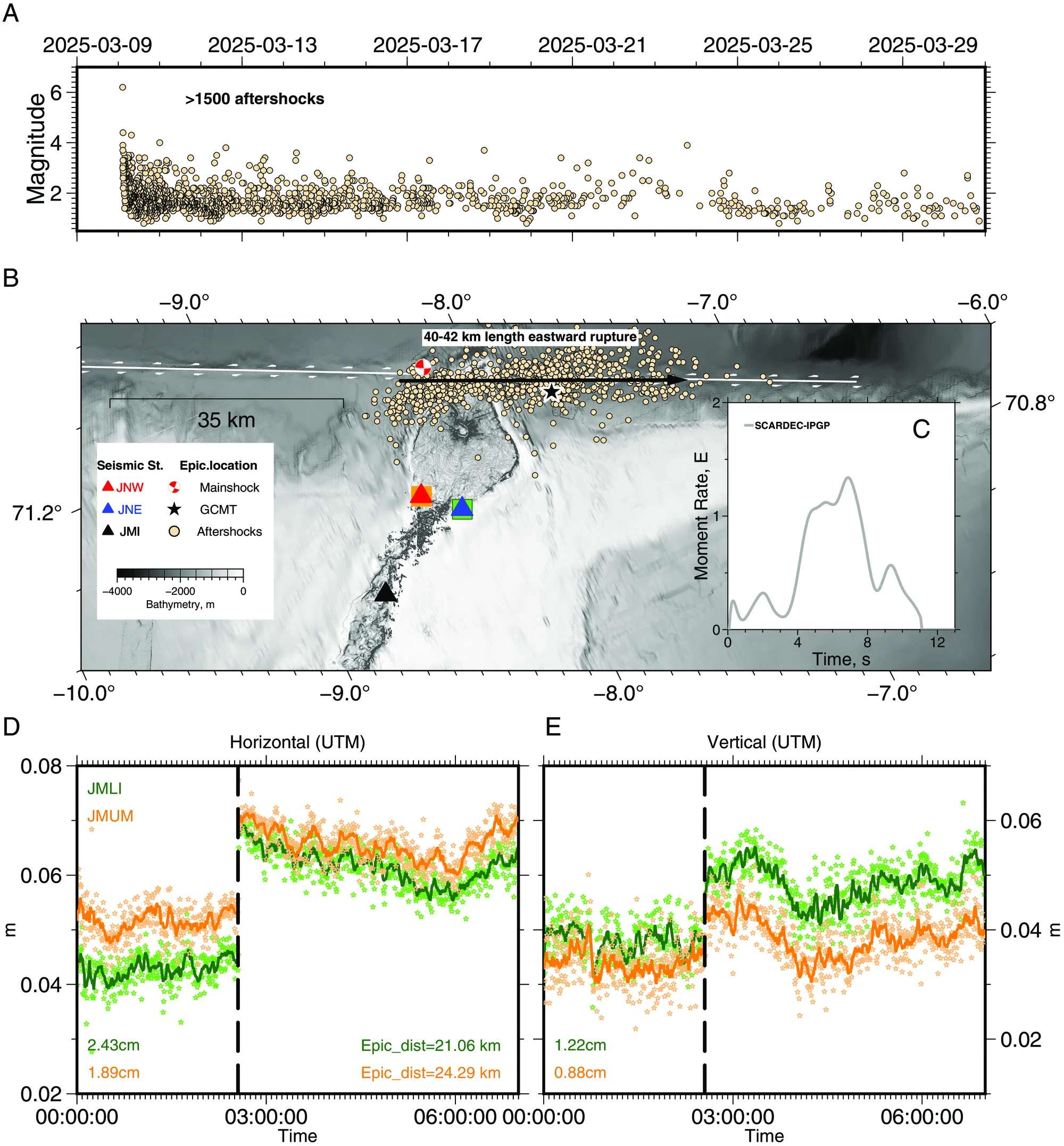
Earthquake source characteristics, aftershock distribution, and geodetic observations of the 10 March 2025 Mw 6.5 mainshock. (*A*) magnitude distribution of the seismicity over time. Beige circles show the whole mainshock-aftershock earthquake activity occurring between March 10 and 30, 2025 as reported by the NNSN (28). (*B*) bathymetric map of the JMI and JMTF, indicating unilateral rupture of the mainshock toward the southeast. The local seismic stations are represented in triangles. Red focal mechanisms indicate the relocated epicenter of the mainshock with the focal mechanism reported by the GCMT (2, 3). Beige circles refer to the relocated epicenter of the aftershock seismicity along the transform fault. The black star indicates the centroid location of the GCMT, correlating with the central area of the aftershock distribution. Moment Rate obtained by the SCARDEC (31, 32), presented on (*C*). (*D* and *E*) Horizontal and Vertical GPS derived ground displacement, being the location of the JMLI and JMUM ground motion GNSS stations presented by the green and orange squares in (*B*). Vertical dashed lines indicated the mainshock origin time.

The Norwegian National Seismic Network (NNSN) (28) reported >1,500 aftershocks between 10 to 31 March (Fig. 2*A*) with magnitudes of 0.7 to 4.2. We relocated 1443 events using a Bayesian approach (29, 30) considering both local and regional seismic stations (*Methods* and *SI Appendix*, *Supplementary Text S1*). More than 500 events had horizontal uncertainties <10 km (*SI Appendix*, Figs. S5–S7). Spatially, the aftershocks indicate rupture initiation near the relocated mainshock epicenter and its southeastward propagation for ~40 to 42 km along the JMTF (Fig. 2*B*). Regional P-wave durations of ~10 to 12 s suggest rupture velocities of 3.3 to 4.0 km/s along a 110° azimuth (*Methods* and *SI Appendix*, Fig. S8 and Fig. S9), matching the moment rate of the Seismic source ChAracteristics Retrieved from DEConvolvolving (SCARDEC) (31, 32) catalog (Fig. 2*C*) and aftershock distribution along the geometry of the JMTF (~110° to 114° strike; Fig. 2*B*).

Two permanent GNSS stations recorded coseismic surface displacements, revealing horizontal shifts of 1.9 to 2.4 cm (Fig. 2*D*) and 0.9 to 1.2 cm in vertical of the Universal Transverse Mercator projection (UTM; Fig. 2*E*), respectively. Both stations show a brief postseismic transient offset, but the final static offsets are stable and consistent with the expected southeastward coseismic displacement. Ground motions of Intensity IV (MMI) were felt in Olonkinbyen (Fig. 1*B*), where the Norwegian Armed Forces station is located (*SI Appendix*, *Supplementary Text S1* and Figs. S9 and S10). Together, these results on rupture behavior and surface displacement indicate that the earthquake caused southeastward ground displacement that strongly shook Kjerulf Glacier.

## Seismic Velocity Changes

**Fig. 3.**
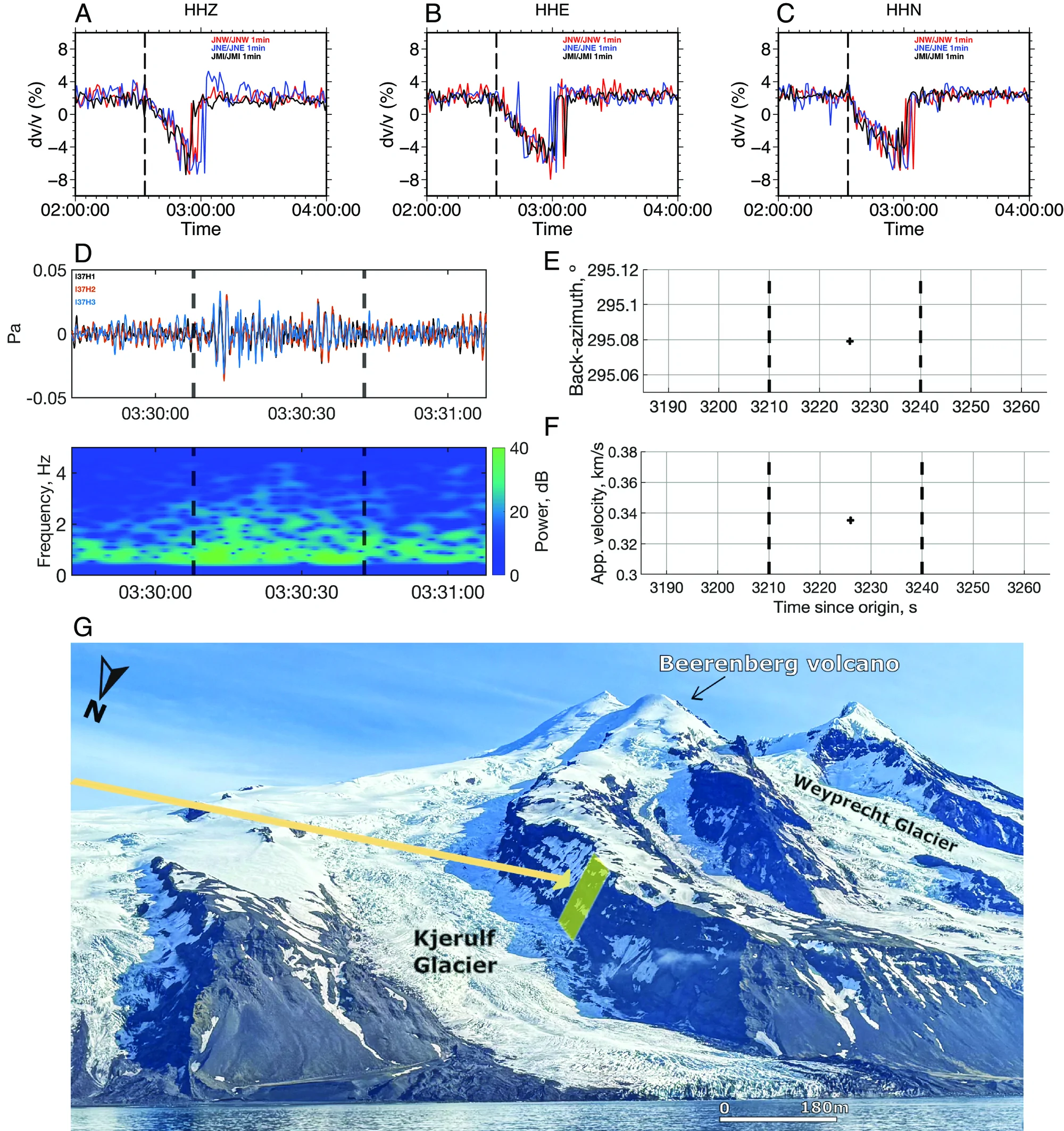
Seismic velocity changes, infrasound, and local view of the Kjerulf Glacier. (*A*–*C*) show relative seismic velocity changes (dv/v) at the onset of seismic rupture and ground motion are observed at the three local seismic stations on Jan Mayen. The vertical black dashed line indicates the mainshock origin time measured by the local seismic stations (02:33:15.8 UTC). (*D*) *Top*: acoustic signal recorded by the infrasound sensors of the NORSAR (39) CTBTO (vDEC, www.ctbto.org/specials/vdec/) network presented by the black/red/blue waveforms. The vertical black dashed lines indicate the signal range cross-correlated in analysis using the back-azimuth approach (7). *Bottom*: spectrogram of the same signal plotted in the *Upper* part of (*D*). (*E*) Back‐azimuth obtained from cross‐correlation of three‐infrasound pairs is presented by the black cross on top plot. The apparent velocity referent of the infrasound propagation is shown on (*F*). (*G*) Orthogonal structure view (northward) of the Kjerulf glacier and Beerenberg volcano at south, before the earthquake. Image adapted from original photographed by Robert Michael Poole, 27 June 2024. (with permission). Yellow 40% transparent shape indicates the rock avalanche source area along the south basaltic wall. The back-azimuth direction obtained by the infrasound signal is shown by the yellow arrow.

The 10 March 2025 Mw 6.5 earthquake generated strong seismic ground motion at the JMI. Natural phenomena like earthquakes (33–36), icequakes (37), and landslides (20, 38), can directly affect the local seismic velocity structure. Our results of seismic noise interferometry from the cross-correlation process (*Methods* and *SI Appendix*, *Supplementary Text S2*) show a short-term reduction of ~7% in local seismic velocity for ~30 min (Fig. 3 *A–C* and *SI Appendix*, *Supplementary Text S2*) starting a few minutes after the mainshock origin time. The rapid decrease is consistent with changes in the dynamic elasticity of shallow crustal rocks and soil. Given the 0.2 to 0.5 Hz frequency band used in analysis (*Method* and *SI Appendix*, *Supplementary Text S2*), the inferred dv/v changes integrate over the upper crust (approximately 1 to 3 km depth, depending on local shear-wave velocities), rather than reflecting only the shallow geotechnical layer (e.g., the Vs30 depth range), or the deeper seismogenic zone. Temporal changes in seismic velocity (dv/v) caused by strong earthquakes can be observed for months or years (40–42), including strike-slip events that occurred on oceanic transform faults (33). In contrast, the dynamic velocity decrease identified here recovered within less than one hour. We are unaware of previous reports documenting such a short-lived earthquake-induced dv/v perturbation, suggesting that the observed response is unusual.

The observed decrease in seismic velocity after the earthquake can be explained by different mechanisms. Strong seismic waves most likely generated small cracks and mineral grain delamination in near-surface rocks, reducing elastic moduli and weakening the material (41, 42). Dynamic shaking in volcanic and glaciated terrain may also raise pore fluid pressure in cracks or beneath ice, reducing effective stress and stiffness (43). Furthermore, coupling with neighboring glaciers could have increased surface wave energy or changed near-surface stress conditions at the ice–rock interface (19). These combined impacts may result in an enhanced dv/v response, demonstrating the ability of ambient seismic noise interferometry to detect changes in shallow subsurface properties following strong earthquakes, which may also be sensitive to surface processes.

## Infrasound Signals

Acoustic signals originating ~3 min after the earthquake were detected at the infrasound array I37N in Norway and the BORG station installed in Iceland (*Methods* and *SI Appendix*, Fig. S11). The signal propagated at a velocity of ~0.290 km/s to BORG arriving at ~3,220 s (*SI Appendix*, Fig. S12). Our estimates from the I37N array (Fig. 3*D* and *Methods*) indicated a source of the infrasound signal at a back-azimuth of 295.07° ± 0.1° (Fig. 3*E*) and an apparent velocity of 0.336 km/s (Fig. 3*F*). At the source distance of the I37N array, the back-azimuth angular uncertainty corresponds to a lateral positional uncertainty of ~1.5 km (*Methods*). The back-projection of the ray tracing produced by the back-azimuth points to the Kjerulf Glacier area, where satellite images reveal slope failure after the earthquake (Fig. 3*G*). Based on apparent velocities, the infrasound event originated between 02:36:50 and 02:37:20 UTC.

Several lines of evidence indicate that the infrasound signals did not originate from the earthquake rupture in the transform fault. The Mw 6.5 event occurred away from the infrasound source area at ~18 km beneath the sealevel and involved dominantly strike-slip motion, conditions that couple seismic energy extremely poorly into the atmosphere (44). Detectable energy in the infrasound originating from seafloor earthquakes is generally limited to shallow and tsunamigenic events with large vertical seafloor displacement (45–47). In contrast, the efficient acoustic radiation produced by energetic surface mass movements such as snow or rock avalanches generate infrasound signals that propagate over large distances (18, 48, 49).

Given the source location coincident with the mapped head scarp (Fig. 3*G*), the non-tsunamigenic strike-slip mechanism, the offshore hypocenter, and the timing of the signal relative to the dv/v drop, the most consistent interpretation is that the infrasound was generated by a large postseismic rock avalanche on the Kjerulf Glacier triggered by the Mw 6.5 mainshock. The smaller precursor failures were not detected by the regional seismic and infrasound array. These observations indicate that combined seismic noise and infrasound monitoring can identify rapid, short-lived changes associated with large earthquake-triggered slope failures, while smaller failure events may remain below the detection threshold.

## Rock Slope Failure and Rock Avalanche

We examined the USGS Ground Failure automatic model, which warned of potential earthquake-triggered rock slope failure after the 10 March 2025 Mw 6.5 earthquake (*SI Appendix*, Fig. S9) (12). To independently check the expected shaking at the Kjerulf Glacier, we modeled the earthquake ground motion (*SI Appendix*, *Supplementary Text S3* and Figs. S13 and S14). The results indicate that the Kjerulf Glacier experienced peak ground acceleration (PGA) of approximately 0.40 to 0.45 g, peak ground velocity of 45 cm/s, and 0.2-s spectral acceleration (SA0.2) approaching 1.0 g (*SI Appendix*, Figs. S13–S15). The modeled PGA substantially exceeds the ~0.05 to 0.11 g lower-bound threshold commonly associated with the onset of earthquake-triggered landslides (50, 51), indicating that the earthquake generated sufficiently strong shaking to initiate the slope failure. Sentinel-2 optical (10 m resolution) imagery (4) revealed significant pre- and post-earthquake (*SI Appendix*, Figs. S21 and S22) changes on the south wall of Kjerulf Glacier that had not been observed in previous years, with debris deposits being evident in images taken from 24 April 2025 by Sentinel-2 (*SI Appendix*, Fig. S22), and local oblique view on 5 May 2025 compared to another taken on 8 June 2005 (*SI Appendix*, Figs. S23 and S24). By studying the imagery, we identified a large rock slope failure that occurred following the seismic event. The timing of the pictures and infrasound records, indicating that the rock slope failure and rock avalanche happened within ~3 min after the mainshock. However, local seismic waveform confirmation of the rock slope failure from the local seismic stations was not possible as the signals were masked by the long seismic coda of the mainshock.

**Fig. 4.**
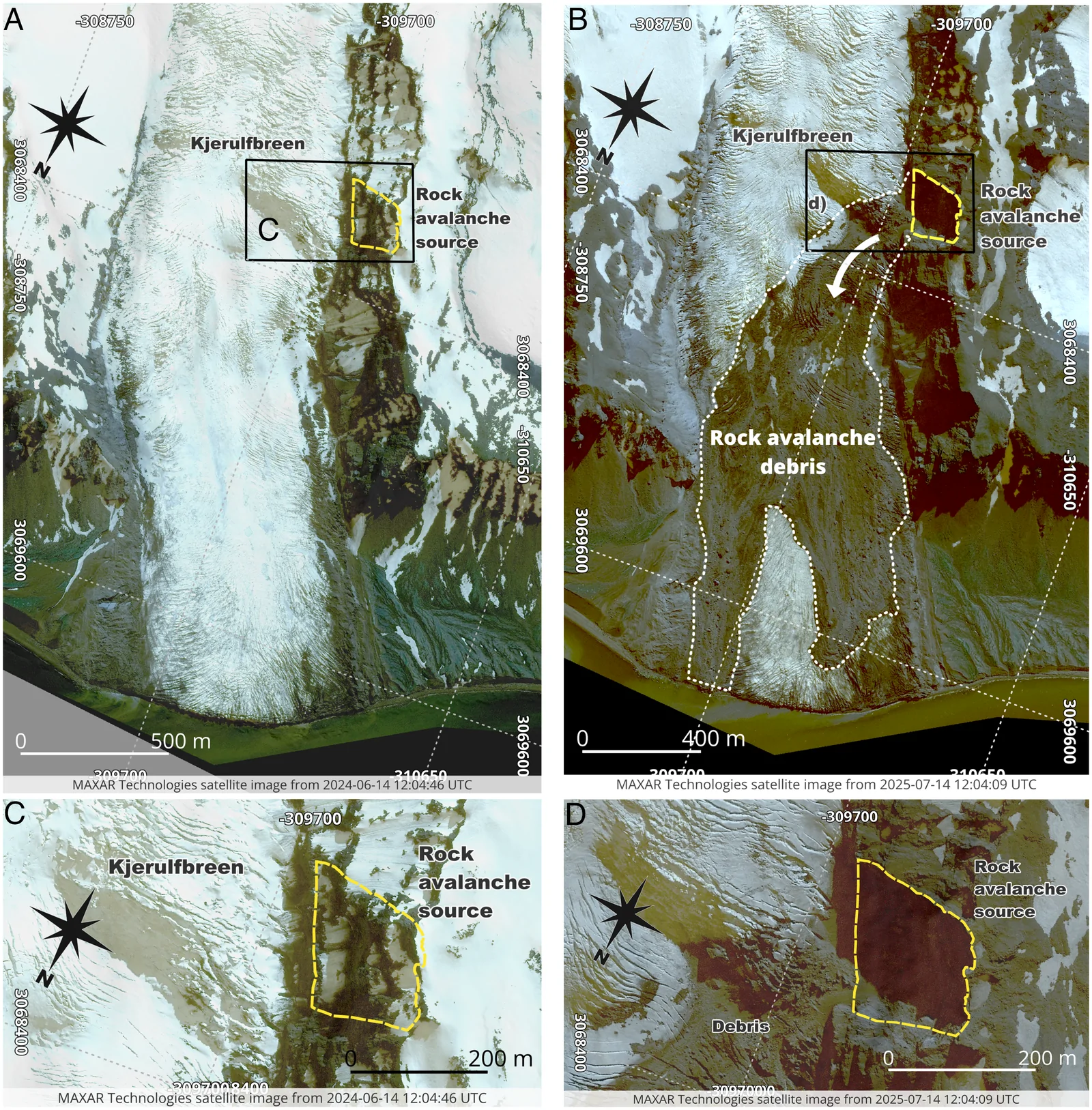
High-resolution satellite observations documenting the Kjerulf Glacier pre- and post-earthquake induced rock avalanche. (*A*) Pre-earthquake MAXAR satellite image of the Kjerulf Glacier from 14 June 2024, with a zoom-in of the southern rock wall where the rock failure occurred, source of the rock avalanche (*B*) (Satellite image © 2025 Maxar Technologies provided by European Space Imaging). (*C* and *D*) Post-earthquake MAXAR satellite image of the Kjerulf Glacier from 14 July 2025 (Satellite image © 2025 Maxar Technologies provided by European Space Imaging), with debris distribution (dashed white line) clearly visible along the glacier surface (*B*). The yellow dashed line outlines the source area of the rock slope failure before (*C*) and after (*D*) the earthquake.

High-resolution (0.5m) MAXAR images (52) (*Methods* and Fig. 4) revealed rock avalanche deposits covering the southern slope of the glacier that originated at ~800 m above sea-level (https://livingatlas2.arcgis.com/arcticdemexplorer/) (71.1262° latitude, −8.1473° longitude). This is located ~7 km away from the mainshock, representing the deposits of the suspected slope failure (*Methods* and *SI Appendix*, *Supplementary Text S4* and Figs. S16 and S17). Compared to pre-earthquake imagery of 2024 (Fig. 4 *A* and *C*), a debris sheet covered approximately 0.9 km^2^ of the glacier surface and reached the coastline after the 800 m vertical descent (Fig. 4 *B* and *D*), over a runout distance of 2.3 km, corresponding to a minimum H/L ratio of 0.35 (*SI Appendix*, *Supplementary Text S4* and Fig. S18 and Table S3). The displaced material produced a headwall scarp and a broad debris deposit extending across the glacier surface, confirming these features as a rock avalanche overrunning the glacier (53). The debris spreading over the glacier surface likely occurred at high velocities after the rock detachment (54). According to MAXAR images and ArcticDEM (https://livingatlas2.arcgis.com/arcticdemexplorer/) analysis using the Sloping Local Base-Level (55) (*Methods* and *SI Appendix, Supplementary Text S5* and Table S3 and Figs. S19 and S20), the rock avalanche moved $0.81\;\mathrm{to}\;1.17\times 10^{6}\;m^{3}$ of rocks. Because the rock avalanche reached the shoreline (Fig. 4*B* and *SI Appendix*, Figs. S16 and S17), we investigated if the event generated a detectable tsunami signal. Tide-gauge records from the Ittoqqortoormiit station (Scoresbysund, East Greenland) located ~500 km away, show no anomalous sea-level oscillations after the earthquake, with a maximum oscillation of 0.15 m in the first 10 h after the earthquake (*SI Appendix*, *Supplementary Text S5* and Figs. S25–S27), suggesting that the rock avalanche did not generate a measurable regional tsunamigenic wave.

In addition to the large 2025 rock failure, Sentinel-2 imagery (4) reveals that the south wall of Kjerulf Glacier experienced four earlier and much smaller rock-slope failures in 2017, 2019, 2023, and 2024 (*SI Appendix*, *Supplementary Text S6* and *SI Appendix*, Figs. S28–S31). A small failure occurred ~450 m south of the 2025 source area in late 2019, while another failure occurred in October 2023 approximately 400 m north of the 2025 site. A third rather minor slope failure occurred in October 2024 within the same structural domain as the 2025 detachment. None of these failures were associated with earthquakes in the NNSN catalog. Their spatial clustering around the 2025 head scarp indicates that the slope had been progressively weakening over several years. These repeated minor failures demonstrate a ~9 y pattern of instability that predisposed the basaltic rock wall to failure once strong shaking occurred in 2025.

In addition to the slope failure, satellite radar and optical data link the earthquake to a calving event on Weyprecht Glacier (*Methods* and *SI Appendix*, *Supplementary Text S8*), located ~2 km southwest of the Kjerulf Glacier shoretline. Sentinel-1 SAR backscatter imagery (4) taken only 4 h after the mainshock origin time revealed increased backscatter offshore in front of the glacier (*SI Appendix*, Fig. S32), indicating detached ice blocks, while MAXAR imagery confirmed a marked retreat of the glacier front and newly exposed crevasse fields on 14 July 2025 (*SI Appendix*, Fig. S33), as well as local images of the Weyprecht Glacier front taken pre- and post-earthquakes (*SI Appendix*, Figs. S34 and S35). In concert, these datasets show that the earthquake caused both slope failure at Kjerulf Glacier and glacier calving at Weyprecht Glacier, emphasizing the multihazard response of Arctic cryospheric systems to tectonic earthquakes.

## Local Air Temperature Increases From 1970 to 2025

**Fig. 5.**
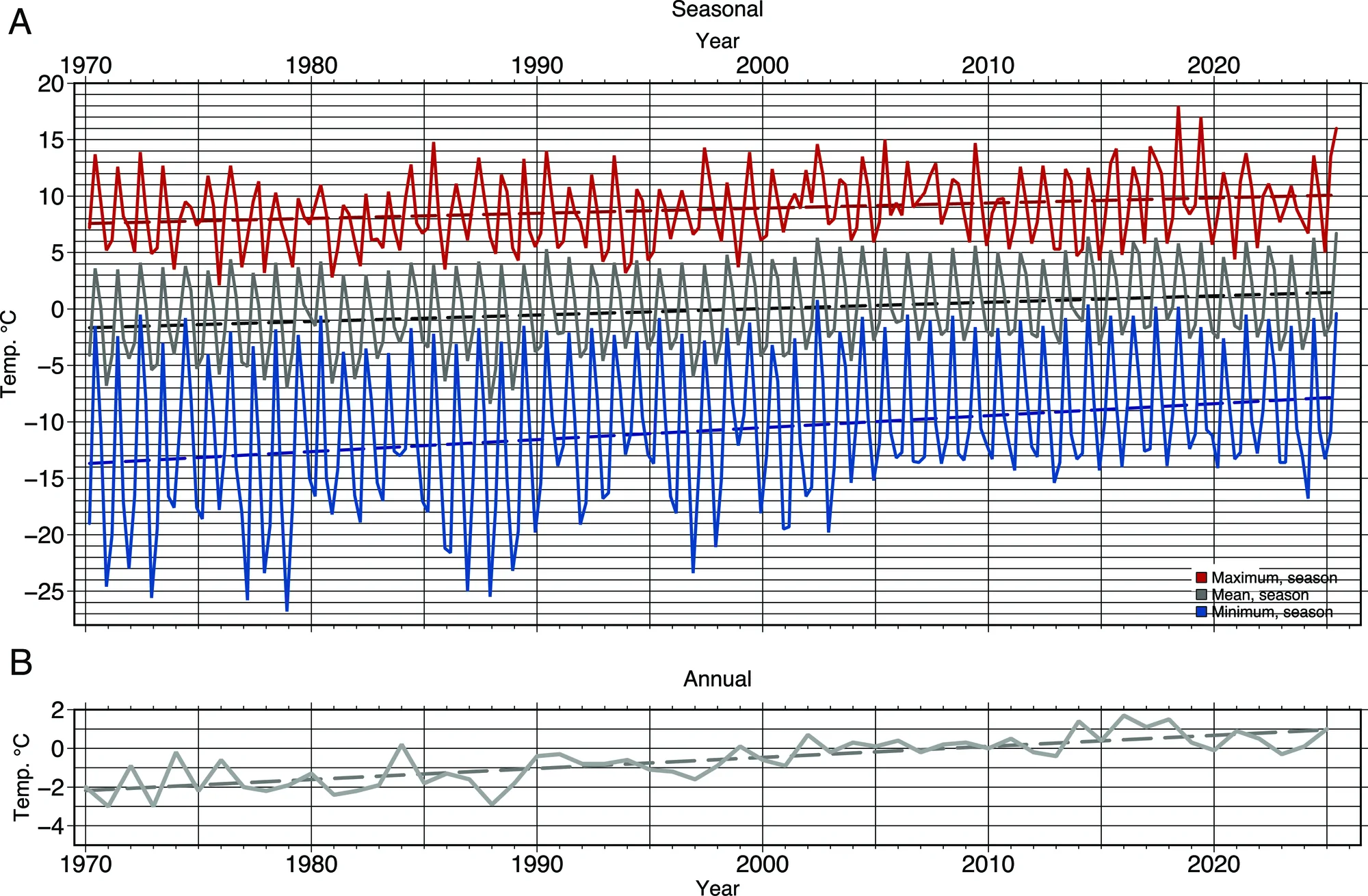
Long-term air temperature records at JMI between 1970 and 2025. (*A*) Long-term distribution of the local maximum, mean, minimum seasonal air temperatures distributed along the four seasons between 1970 and 2025. Dashed lines represent linear least-squares regression to the temperature records. (*B*) Long-term distribution of the annual mean temperatures at JMI, showing a gradual increase, with the annual mean temperatures exceeding 0 °C after 2000.

To evaluate potential long-term environmental controls on slope stability, we analyzed the long-term (~55 y) local air temperature records at JMI. The records show a consistent warming trend with Arctic amplification, with the summer mean temperatures (June) increasing from ~2 to 3 °C in the 1970s to ~5 to 6 °C in recent years (*Methods* and Fig. 5*A*), while winter means (December) shifted from about −6 to −7 °C to −2 to −1 °C. The extreme cold events below −20 °C common in the 1970s–1980s (which reached −27 °C in 1979) changed much more profoundly, becoming rare since the late 1990s, with minimal winter temperatures typically reaching −12 to −14 °C in the 2010s–2020s. In parallel, summer maximum temperatures rose from 11 to 13 °C in the earlier records (1970s–1980s) to episodic extremes of 17 to 18 °C after 2000. Similarly, the annual mean temperatures show a progressive increase of ~2 °C between 1970 and 2025 (Fig. 5*B*), with anomalies shifting from predominantly negative values prior to ~2000, to near-zero or positive values reaching 1.7 °C during the last two decades. These changes illustrate both the loss of severe cold periods and the emergence of anomalously warm summer days.

These trends are consistent with broader Arctic-wide signals. Temperature changes occurring during the cold winter season are accelerated by surface albedo feedback and decreased sea ice (56), as evidenced by the increased winter warming at JMI. We see that the occurrence of higher summer maximum temperatures parallels the warming Arctic summers, which in turn is associated with sea ice loss (57). Furthermore, the disappearance of very low winter minimum temperatures is consistent with findings of decreased temperature variance across the Arctic (58, 59). Therefore, the air temperature record from Jan Mayen highlights a sustained warming trend that may have influenced the slope stability over time, consistent with the occurrence of the small, end of summer rock failures in 2017, 2019, 2023, and 2024.

## Discussion and Conclusion

Our study demonstrates how earthquake ground shaking can affect and cascade through multiple environmental systems in the Arctic. Lithospheric faulting, strong ground shaking, and temporal changes in rock properties which in turn resulted in slope failure, glacier modification, and atmospheric infrasound signals. By combining local seismic data, waveform modeling, GNSS time series, seismic noise interferometry, infrasound detection, and high-resolution satellite imagery, we reconstruct a dynamic chain of events in which intense shaking from the Mw 6.5 Jan Mayen earthquake triggered a rock-slope failure and resulted in a debris-covered glacier.

Earthquakes of Mw up to 7.0 occurred previously on the JMTF, the last one occurring in 1951 (6). The USGS reports four earthquakes of Mw 5.6 to 6.0 close to the 2025 Mw 6.5 epicenter in 1988, 1997, 2000, and 2004 (6) (*SI Appendix*, Table S5). Our ground-motion modeling indicates that the Kjerulf Glacier rock avalanche source area experienced peak ground acceleration of ~0.40 to 0.45 g, peak ground velocity of 45 cm/s, and 0.2-s spectral acceleration approaching 1.0 g (*SI Appendix*, Figs. S13–S15), values well above the lower-bound shaking threshold commonly associated with earthquake-triggered landsliding (50, 51). Yet despite repeated seismic activity and inferred strong ground shaking, no earthquake-triggered rock avalanches with a similar debris deposit extension have been documented on the JMI or observed by Landsat satellite images for the last 40 years (earthquakes occurred in 1988–2004; see *SI Appendix*, Supplementary Text S9 and Table S5 and Figs. S36–S49). Previous mass movement events include a submarine landslide occurring on the Jan Mayen Ridge (60) and a snow avalanche that killed two people in 2021 (61), both unrelated to earthquakes. The 2025 event therefore represents the first scientifically documented case of earthquake-triggered rock failure on Jan Mayen, and the first known case of a rock avalanche burying most of the glacier surface area (30 to 50%). Debris-covered glaciers typically require years to decades to sequester the rock avalanche debris into the glacier (14, 21, 62), underscoring the long-lasting environmental effect of such events in fragile Arctic areas.

Sentinel-2 imagery evidence of four precursor failures in 2017, 2019, 2023, and 2024 (*SI Appendix*, *Supplementary Text S6* and Figs. S28–S31) further indicates that the Kjerulf Glacier south wall had already entered a prolonged phase of progressive weakening prior to the Mw 6.5 earthquake. These failures occurred without any local seismic activity and are consistent with slow gravitational deformation and stress redistribution within the rock mass. The spatial proximity of these events to the 2025 headscarp suggests that the slope was approaching a limit-equilibrium state, and the 2025 shaking acted as the final trigger releasing an already compromised rock mass. The temporary ~7% decrease in near-surface seismic velocity lasting approximately 30 min after the earthquake is consistent with a short-lived perturbation of the shallow medium during intense ground shaking. These observations primarily indicate transient changes in the mechanical properties of the material surrounding the seismic station (within approximately 1 to 3 km), rather than within the landslide source area itself. However, it is reasonable to hypothesize that similar mechanical perturbations also occurred in the landslide area. If so, they may have temporarily weakened the shallow material, thereby preconditioning the slope for failure. The unusually rapid recovery of the dv/v signal, compared with longer-lasting coseismic velocity reductions observed after strong earthquakes (35, 36, 40, 41), suggests that the local perturbation primarily reflects transient surface shaking associated with the earthquake rather than long-term structural damage in the shallow crust. At the same time, the infrasound array recorded the acoustic signal of the rock avalanche within minutes of the mainshock, confirming that the slope failure occurred rapidly following the seismic shaking. Although the infrasound back-azimuth lateral uncertainty corresponds to approximately 1.5 km, a significant part of this lateral range still intercepts the surface of the Kjerulf Glacier (approximately 650 m wide at the source area), suggesting that the inferred infrasound source remains spatially aligned with the mapped rock-failure area, and not associated with glacial calving at the Weyprecht Glacier. In addition, no tide gauge signal was detected neither in Greenland nor Norway, making alternative explanations for the detected infrasound signal, like a tsunami wave or a failure of a glacier like the Weyprecht ice calving, unlikely.

The impacts of climate change are increasingly evident worldwide (18, 63–67), and in Arctic environments, these changes may act as conditioning factors that weaken slope stability over long time scales. Thus, warming serves as a long-term preconditioning influence that may amplify the impacts of natural hazards. Recent work also shows that ongoing permafrost degradation is destabilizing Arctic terrain more broadly (68), reinforcing the interpretation that long-term climatic warming can precondition steep rock slopes to failure. Arctic warming in recent decades (13) has weakened permafrost (69, 70) and reduced the stability of glacier-adjacent rock slopes (18). Permafrost degradation accelerates meltwater infiltration and promotes crack growth, predisposing volcanic slopes to failure (63, 71–74). Seasonal air temperature records show a clear Arctic amplification effect, with extreme cold temperatures in winter largely absent since the late 1990s (Fig. 5*A*), and summers increasingly punctuated by anomalously warm days (56–59). This is consistent with broader Arctic observations (56, 61), corroborating the high permafrost degradation in recent decades caused by the Arctic amplification (75–77). Enhanced summer heat and exposure of steep rock faces likely further accelerate fracture propagation and weakening of slopes (77). The local temperature records shows a persistent warming with annual mean anomalies shifting ~2 °C from 1970 to 2025 (Fig. 5*B*). Arctic observations and permafrost models indicate that atmospheric warming commonly propagates into the ground, producing permafrost temperature increases of ~0.3 to 1.0 °C per decade and correlates with the rapid decline in shear strength of ice-filled fractures (78, 79). Laboratory experiments show that near the melting point the strength of ice-cemented joints can decrease by more than 50% (80). Under these conditions, rock temperatures in the failed slope approached the critical thawing range and preconditioned the rock mass for failure. Under these conditions, the 2025 earthquake was able to trigger slope failure that might not have occurred under colder permafrost conditions in the past. Furthermore, the previous four small failures align with the expected behavior of rock slopes progressively destabilized under warming conditions and reduced permafrost cohesion. These precursor small failures reinforce the interpretation that environmental weakening played a key conditioning role, while the oceanic transform Mw 6.5 2025 earthquake served as the ultimate trigger.

Taken together, our observations document a cascade linking earthquake rupture, rock slope failure, glacier disruption, and atmospheric infrasound generation in the Arctic. The rapid onset of the rock avalanche following the Mw 6.5 Jan Mayen earthquake demonstrates how strong seismic shaking can destabilize glacier-adjacent slopes to generate infrasound and induce transient seismic velocity perturbations of shallow mechanical properties. The occurrence of multiple precursor failures prior to the earthquake further suggests that the slope had undergone progressive destabilization before failure. Although the modeled ground motion indicates that seismic ground shaking was sufficient to initiate slope failure, long-term Arctic warming and associated permafrost degradation may have progressively reduced slope stability. Combined with satellite imagery, local temperature, and seismological analysis, these signals highlight the value of multidisciplinary monitoring efforts to fully capture cascading interactions between tectonic hazards, cryosphere instability, and climate changes in polar areas.

## Methods

### Mainshock Location From Local Stations.

We estimated the epicenter of the Mw 6.5 mainshock using rectilinearity polarization analysis of the first-arriving Pg phases recorded at the three local Jan Mayen stations (JNE, JNW, JMI). We did not use the JMIC station due to high amplitude gain and saturated record of the mainshock. Particle-motion polarization was calculated following the Cesca (23, 24) approach, which measures the back-azimuth from rectilinearity coefficients in three components seismic data with high-quality linearity polarization in both the NS-EW and Vertical-Radial components (81). As the initial arrival time reference, we used the P and S phase picking time reported by the Norwegian National Seismic Network catalog (28). However, the P and S phases were inspected and manually repicked at each station using Seismic Analysis Code package (82). We applied a 0.5 to 10 Hz band-pass filter and a −0.05 to 0.4 s time window around the P arrival. Since the JMI is mostly of volcanic origin with basaltic rocks (8), a seismic velocity of Vp = 6.04 km/s and Vp/Vs = 1.851 was applied in the analysis (83). We defined a minimum threshold of 60% in horizontal and vertical rectilinearities. See more detail of the results in *SI Appendix*, *Supplementary Text S1*.

### Mainshock Depth From Regional Waveform Modeling.

We analyzed the focal depth of the mainshock of the 2025 Mw 6.5 Jan Mayen earthquake using a surface waveform modeling procedure. Jan Mayen is positioned geographically between North America, Greenland, and Europe, and hence it is azimuthally well-covered by regional seismic stations. We used broadband data from eight stations in Greenland (DAG, DBG, SCO), Faroe Islands (SOFL), Bear Island (BJO1), and Norway (HAMF, NSS, LOF) located 509 to 1152 km away from epicenter. Data were obtained from the EarthScope Consortium (https://ds.iris.edu/gmap/; https://service.iris.edu/fdsnws/). The large azimuthal coverage complements well the possibility of applying seismological analyses using regional records. We used the ISOLA package (25, 84) to derive the focal depth from the surface wave modeling of the three components. We applied data from the eight seismic stations between 509 and 1152 km away from the epicenter obtained by the single station location (*SI Appendix*, Fig. S1). Prior to the inversion, the data were corrected for instrumental response and bandpass filtered (0.01 to 0.02 Hz). The inversion was processed by fixing the strike/dip/rake angles of the focal mechanism reported by the GCMT (2, 3). A 1D velocity model of the Norwegian Sea obtained from CRUST1.0 (26) was used in the inversion (*SI Appendix*, Table S2), removing the water layer of 2.71 km (27). A total of 18 trial sources were created from 1.5 to 28.5 km depth, with a 1.5 km interval between them, to compare the results in inversion. See more details on *SI Appendix*, *Supplementary Text S1* and Figs. S2–S4.

### Aftershocks Relocation.

The four local seismic stations at Jan Mayen Island (JNE, JNW, JMI, JMIC) recorded the mainshock-aftershock seismic activity of the 2025 strike-slip earthquake. More than 1590 aftershocks are included in the Norwegian National Seismic Network (NNSN) catalog (28) occurring between 10 and 31 March 2025. Events were relocated using Bayesloc (https://gs.llnl.gov/nuclear-threat-reduction/nuclear-explosion-monitoring/bayesloc), a location tool based on a Bayesian technique that provides a rigorous framework. It thoroughly examines the posterior probability distribution of the earthquake hypocenters (29, 30) to obtain robust absolute locations. Bayesloc provides probabilistic new locations and origin times, using travel-time corrections and pick uncertainties calculated for local and regional phases (Pg, Sg, Pn, Sn) using a Markov Chain Monte Carlo inversion (MCMC) to sample the posterior analytical statistical distribution assuming Gaussian errors in arrival times (29). We applied the approach to relocate the NNSN catalog (28) of the aftershock activity using the four local stations at JMI, and regional stations (DBG, DAG, BJO1, BRBA, BRBR, KBS, NOR, KEV, HOPEN, KTK1, SPA0, and JETT; *SI Appendix*, Fig. S5) for 9 earthquakes with magnitude >3.5 of the NNSN catalog. The location was run using the NNSN/AK135 velocity model (85, 86), with a total of 20,000 iterations using five different depths (5, 10, 15, 20, 25 km) to see the best fit with shorter North–South (NS), East–West (EW), and depth uncertainties. See more detail of results on *SI Appendix*, *Supplementary Text S1*.

### Mainshock Rupture Behavior.

We used the spatial distribution of relocated aftershocks to constrain the rupture length of the Mw 6.5 mainshock, similarly to other oceanic transform fault earthquakes (87–89). The clustered relocated aftershock sequence defines a rupture length of 40 to 42 km (Fig. 2 and *SI Appendix*, Fig. S6). Aftershock migration indicates predominant eastward rupture propagation, with an estimated azimuth of ~110°, consistent with the ~110 to 114° strike of the JMTF from multibeam bathymetry (*SI Appendix*, Fig. S7) and the 111° strike of the GCMT focal mechanism. We assessed rupture directivity using the azimuthal distribution of apparent rupture durations (90). We used data from broadband stations of the Global Seismological Network (GSN) to assess the apparent rupture duration at teleseismic distances (2,000 to 6,000 km). Apparent durations are estimated from the length of the P wave pulse, after integrating displacement records and bandpass filtering in the range 0.01 to 0.5 Hz. Despite the uneven azimuthal distribution of seismic stations (*SI Appendix*, Fig. S8), those located toward the ESE show the shortest duration of ~10 to 12 s, while those in the opposite direction are much longer, on the order of ~20 s. This confirms the rupture plane orientation and rupture directivity toward the ESE. Considering the estimated rupture length, range of apparent durations is consistent with a standard rupture velocity of ~3 km/s (90).

### GNSS Surface Displacements.

The Norwegian Mapping Authorities operate two continuous GNSS stations on Jan Mayen Island, JMLI (71.027°N, 8.423°W) and JMUM (70.988°N, 8.293°W), which provide open-access data in RINEX format through the European GLASS GNSS-EPOS platform (91). We used the Precise Point Positioning (PPP) method of the Canadian Spatial Reference System (92) to extract time-series displacement in meters from RINEX data. GNSS readings were taken at 30 s intervals. Surface displacements were estimated throughout 5-h intervals before and after the 2025 Mw 6.5 earthquake (origin time 02:33:14 UTC, USGS), with slight temporal changes in the initial displacement identified at 02:33:30 to 02:34:00, which corresponds to the origin time measured by the local station (02:33:15.8). Since there is a large variation of increase or decrease in movement in the signal before and after the earthquake, we applied a moving mean trending line, where each mean is calculated over a sliding window of length K point across neighboring elements (window length K = 8) (https://www.mathworks.com/help/matlab/ref/movmean.html). We analyzed the data using MATLAB version 9.8 (93). Using this approach, we extracted the displacements presented in Results and Fig. 2.

### Seismic Velocity Changes From Local Noise Interferometry.

We used ambient seismic noise interferometry on continuous seismic recordings from the three local broadband stations (JNW, JNE, and JMI), which are within 10 to 12 km of the Kjerulf and Weyprecht Glaciers, and 20 to 32 km from the relocated epicenter of the 10 March 2025 Mw 6.5 Jan Mayen earthquake. We used the open-source *eseis* toolbox (94) to process the seismic noise data using autocorrelation in three components (HHZ, HHN, and HHE), applying a 0.2 to 0.5 Hz band-pass filter, 40 s lag time (±20 s), and 1-min windows (*SI Appendix*, *Supplementary Text S2*). Other filter frequency bands were tested, but with lower correlation coefficients (<0.8). To assess relative seismic velocity variations, we applied the stretching method (95), which finds the fractional time stretch that optimizes the similarity between reference and perturbed waveforms. The relative change is given by dv/v = –ε, where ε represents the best-fit time stretch factor.

### Infrasound Monitoring of the Rock Slope Failure.

We analyzed infrasound recordings from the I37N array (Bardufoss, Norway; ~1,030 km) of the IMS (International Miscellaneous Stations, International Federation of Digital Seismograph Networks) (96) and the BORG station (BorgarfjörÐur, Iceland; ~905 km) with a single infrasound sensor (*SI Appendix*, Fig. S11). I37NO is operated by NORSAR (39, 97) under the Comprehensive Nuclear-Test-Ban Treaty (CTBTO; vDEC, www.ctbto.org/specials/vdec/) monitoring network. BORG is part of the Global Seismographic Network managed by Scripps Institution of Oceanography (98), with open-access data via the EarthScope Consortium (https://service.iris.edu/fdsnws). Other nearby IMS arrays located in Greenland (I18H) and Bermuda (I51H) did not record the event. At the I37N array, the infrasound signals were detected 3,415 to 3,420 s after the earthquake origin time, with group velocity of ~0.303 km/s (*SI Appendix*, Fig. S12).

We estimated the source area of infrasound signals using the approach (7) which measures the back-azimuth and apparent velocity of acoustic signals using cross-correlation between pairs of infrasound sensors: I37N1, I37N2, and I37N3 (99, 100). Due to low signal-to-noise ratios (~0.82), the infrasound signals were filtered between 0.6 and 4.0 Hz, like methods applied in the analysis of the 2023 Greenland landslide (18). The uncertainties in back-azimuth were estimated from the covariance matrix of the slowness vector following standard array-processing approaches (100). Assuming normally distributed errors, 95% confidence intervals were derived from the diagonal elements of the covariance matrix. The back-azimuth uncertainty (Δθ) was further adapted into the spatial uncertainty of the infrasound source using ΔX=tan (Δθ)X, where X is the distance between the infrasound array and the inferred source region (7).

### Detection of the Rock Failure From Sentinel-2 Satellite Data.

To assess possible coseismic slope failures, we analyzed Sentinel-2 L2A satellite optical imagery with 10 m resolution which we obtained via the Copernicus Browser (4); Sentinel-2 L2A provides daily optical imagery of JMI. The USGS Ground Failure (12) product issued a near-real-time warning of potential landslides in northern Jan Mayen after the 10 March 2025 Mw 6.5 earthquake (*SI Appendix*, Fig. S10), prompting focused analysis of the Kjerulf and Weyprecht Glacier regions. We downloaded the frames in GEOTIFF file format geocoded with the WGS84 geographic coordinates system with 8 bits per pixel. Because of winter season, most of the satellite images taken in the November–March period are fully or mostly cloudy. The closest pre-earthquake images available with open sky were taken on 19 October 2024 (*SI Appendix*, Fig. S21), while postearthquake images with clear visibility were available on 24 April, 3 May, and 10 May 2025 (*SI Appendix*, Fig. S22). Comparing the last cloudless pre- and postearthquake images, we observed that the images revealed clear changes with dark patches on the south wall of Kjerulf Glacier (*SI Appendix*, Fig. S21) that are corroborated by local images (*SI Appendix*, Figs. S23 and S24), where the rock failure occurred with the slope failure depositing debris across the glacier surface and down to the sea margin. No comparable features were observed in seasonal images compared to the images taken since 2018, supporting the interpretation that this rock failure occurred immediately after the Mw 6.5 2025 earthquake. Only the photos that had the clearest sky were compared. The 10 m resolution limits detailed volume analysis, but the observed debris accumulation and altered surface patterns confirm significant postseismic landscape modification.

### High-Resolution MAXAR Observations and Measurement of the Rock Avalanche.

We used high-resolution MAXAR satellite images to estimate the volume of the rock avalanche source area at Kjerulf Glacier (52) (*SI Appendix*, *Supplementary Text S4*). Three panchromatic WorldView-2 images (0.58 m resolution, 8-band) acquired on 14 June 2024 and 14 July 2025 were analyzed in QGIS (101) to compare pre- and postearthquake topography (*SI Appendix*, Figs. S16–S18). Because of the regular Arctic cloud coverage and snowfall in March due to the winter, the images taken from prior years and the postearthquake photos from May and July of 2025 were chosen to guarantee a clear view of the location of the rock failure and the entire debris area covered after the rock avalanche. Extensive debris deposits were found along the Kjerulf Glacier surface in the 2025/07/14 image (*SI Appendix*, Figs. S21 and S22).

The MAXAR imagery was coregistered to the 2 m ArcticDEM with AROSICS (102) and refined with manually selected ground control points (*SI Appendix*, *Supplementary Text S5* and *SI Appendix*, Figs. S19 and S20). The resulting registration obtained a planimetric RMSEr of approximately 0.75 m, confirming accurate alignment between imagery and DEM. The coregistered scenes were then used with the Sloping Local Base-Level (SLBL) method (55) to estimate the prefailure rock volume, yielding likely 0.81 to 1.17 × 10^6^ m^3^. The same dataset was used to determine the source height (H), runout distances (L), debris deposit extent, and H/L ratios (*SI Appendix*, *Supplementary Text S5* and *SI Appendix*, Fig. S18).

### Observation of an Ice Calving in Weyprecht Glacier From Sentinel-1 and MAXAR Images.

We used Sentinel-1A C-band interferometric images (10 m ground resolution, dual HH/HV polarization, ascending and descending passes) acquired over Jan Mayen to verify possible occurrence of ground displacements postdating the main earthquake. We obtained data in GeoTIFF format and WGS84 geographical system from the Alaska Satellite Facility (https://search.asf.alaska.edu/). The interferometric synthetic aperture radar (InSAR) processing was attempted using GMTSAR (103), but low coherence due to the mostly snow-covered surface on 10 March prevented displacement retrieval. In addition, we visually compared Sentinel-1 SAR backscatter imagery from the descending frame 355 (path 67) from 26 February 2025 (07:07:24 UTC) and 10 March 2025 (07:07:42 UTC, ~4.5 h after the Mw 6.5 earthquake) from the Copernicus Open Access Hub (4) webpage (*SI Appendix*, Fig. S32), with images revealing a glacier-front collapse at Weyprecht Glacier, with high backscatter offshore indicating detached ice flowing into the sea. This was confirmed by comparison with MAXAR imagery acquired on 14 June 2024 and 14 July 2025 (*SI Appendix*, Fig. S33), and local pictures (*SI Appendix*, Figs. S34 and S35). See more detail on *SI Appendix*, *Supplementary Text S5*.

### Local Seasonal Air Temperature Variability (1970–2025) and Arctic Amplification at JMI.

We analyzed seasonal (March, June, September, December) mean, maximum, and minimum air temperature records from JMI spanning 1970–2025, using data of the local meteorological station (SN99950) installed on the island (70.939167° latitude, −8.668889° longitude). The temperature data are accessible to download on the Norwegian Centre for Climate Services webpage (https://klimaservicesenter.no/), and the mean, maximum, and minimum seasonal air temperatures can be extracted directly on webpage.

## Supplementary Material

Appendix 01 (PDF)

## Data Availability

The seismic data of the local stations installed at Jan Mayen Island (JNE, JNW, JMI, JMA) and used in this study are available for download from the webpage of the Norwegian National Seismic Network (28). The data of the eight regional broadband stations (BJO1, HAME, LOF, NSS, SOFL, SCO, DBG, DAG) located 509 to 1,152 km from the epicenter and used in waveform modeling can be downloaded at EarthScope Consortium Inc webpage (https://service.iris.edu/). The geodetic data of the two local sensors (JMLI and JMUM) used in displacement measurement are available to download on European GLASS GNSS-EPOS webpage (82). The infrasound acoustic data of the I37 array were made available by NORSAR (39, 97) and the CTBTO International Data Centre, Vienna, through the virtual Data Exploitation Centre (vDEC, www.ctbto.org/specials/vdec/). We analyzed and processed the infrasound data using MATLAB version 9.8 (93) with toolboxes (Control System; Signal Processing; Mapping; Antenna; Communications; Phased Array System) and other external codes obtained in MathWorks File Exchange (e.g., DEG2UTM; RDSAC) (104, 105). The Sentinel-1 SAR imagery (SLC) and Sentinel-2 optical images are freely available from the Copernicus Open Access Hub (4), with the SAR data also available at the Alaska Satellite Facility (https://search.asf.alaska.edu/). Commercial MAXAR optical satellite imagery (WorldView-2) of JMI, acquired on 14 June 2024 and 14 July 2025 were obtained under license from Maxar Technologies and is not publicly available. The Landsat satellite imagery used in *SI Appendix*, *Supplementary Text S7* is publicly available from the U.S. Geological Survey Landsat program and was accessed via EOSDA LandViewer (https://eos.com/landviewer). Figs. 1 and 2*B* uses bathymetry downloaded from the Global Multi‐Resolution Topography (https://www.gmrt.org/GMRTMapTool) (106) and the segmentation of the Mid‐Atlantic Ridge is from MAPRIDGES (1). Previously published data were used for this work (https://scihub.copernicus.eu; http://geoscope.ipgp.fr/index.php/en/catalog/earthquake-description?seis=us6000pxvx; https://earthquake.usgs.gov/earthquakes/eventpage/us6000pxvx/ground-failure/summary; https://nnsn.geo.uib.no).
