# Supplementary material for: Earthquake-triggered cascading hazards under Arctic amplification
Source: Proc Natl Acad Sci U S A. 2026 Sep 8;123(38):e2617210123. doi: 10.1073/pnas.2617210123 (PMC13598256; doi:10.1073/pnas.2617210123)
Supplement: Supplementary file 1 [file pnas.2617210123.sapp.pdf]

PNAS

Supporting Information for

Earthquake-triggered cascading hazards under Arctic amplification

Guilherme W. S. de Melo[1*], Reginald L. Hermanns[2], Jacob M. Bendle[2], Ingo Grevemeyer[1], Sylvain Fiolleau[2], Simone Cesca[3], Aderson F. do Nascimento[4], Lars Ottemöller[5], Gökhan Aslan[2], Quentin Brissaud[6], Volker Oye[6], Heidrun Kopp[1,7].

1-GEOMAR Helmholtz Centre for Ocean Research Kiel, Kiel, Germany
2-Geological Survey of Norway, Trondheim, Norway
3-GFZ Potsdam, Potsdam, Germany
4-Departamento de Geofisica, Federal University of Rio Grande do Norte, Natal, Brazil
5-Department of Earth Science, University of Bergen, Bergen, Norway
6-NORSAR, Kjeller, Norway
7-University of Kiel, Kiel, Germany

*Guilherme W. S. de Melo

Email: gdemelo@geomar.de

This PDF file includes:

Supporting text S1-S8
Figures S1 to S49
Tables S1 to S5
SI References

Text S1. Seismic analysis of the Mw 6.5 mainshock and the aftershock activity

(i) Epicenter location of the mainshock from the rectilinearity polarization

The epicenter location can be obtained with higher accuracy and lower uncertainty using the first P wave phase polarity [1,2]. Since the location of the earthquake source can directly influence the scattering of the seismic energy, the record quality of the seismic event will depend on the source location compared to the station location. From that, local stations placed close to the seismicity can reveal the epicenter location with good accuracy from the direct ray of the P wave (Pg phase; <50-70 km away). The Pg phase is fundamentally useful to provide the direction of the earthquake source from the good polarization provided by local records of the earthquake [3]. See Methodology for more details.

The results from three stations on Jan Mayen provide a NS-EW component rectilinearity (see Methodology) of 0.76 (JMI), 0.63 (JNE), and 0.72 (JNW). The Vertical-Radial components indicates a rectilinearity of 0.86 (JMI), 0.89 (JNE), and 0.82 (JNW). We fixed an initial earthquake depth of 10 km. The three stations presented three different final latitude and longitude coordinates in epicenter location, with a maximum distance of 3 km between them. To improve the accuracy, we ran the code again multiple times changing the depth in steps of 1 km to search for the minimum-misfit focal depth at which the three epicenters approach each other. The best solution is at 18 km depth and the epicenter is located at 71.19353º latitude and -8.19052º longitude (see Figure S1), which refers to the mean of the three final coordinates obtained by each station at 18 km depth. A horizontal error of 0.88 km was estimated calculating the maximum distance between the three epicentral coordinates obtained to each station.

(ii) Focal depth of the mainshock

Our results of the surface waveform model (see Methodology) presented the best synthetic/real waveforms (Figure S3) with a focal depth of 16 km with a condition number (CN) of 2.5 and varied reduction (VR) of 0.55 (Figure S2 and S4). Reference [4] consider ill-posed solutions when the results indicate the CN>10 and VR<0.4. This procedure showed a focal depth of 2 km shallower than the single station location (18 km). However, the discrepancy can be explained by the fact that the 2.7 km water layer was removed from the velocity model (Table S1) to run the waveform inversion to provide a common velocity structure for the local seismic stations occurring above sea level. This indicates that assuming sea level, the focal depth would be 18.7 km, matching the results obtained from the single station analysis. Therefore, these results support the previous focal depth using the single station technique, indicating that the source of the seismic energy was located 18-18.7 km below the sea level at epicentral location (71.19353º latitude, -8.19052º longitude). Comparison of the depth with the other global networks and their epicenter location are shown on Table S2.

(iii) Relocation of the aftershock activity from the NNSN catalog

The Bayesloc approach used in relocation (see Methodology) has been previously applied in relocating earthquakes in the Arctic Sea using both local Ocean Bottom Seismograph and regional land stations located in Greenland, Svalbard, Norway, and Jan Mayen Island (Figure S5) [5,6]. The results of the regional epicenter relocation indicates that a total of 1443 epicenters were relocated from the 1523 total aftershocks in the NNSN catalog [7] that occurred in the first 21 days (Figure S6). The results indicate horizontal uncertainties of <30 km. More than 500 events (35% of the total) have horizontal uncertainties <10 km (Figure S7). The depth varies from 5.5 to 21 km beneath the sea level, with a maximum uncertainty of 10 km. Larger uncertainties are prevalent in events relocated from regional stations. We did not carry out a more comprehensive examination of earthquake depths due to the space distribution of the 12 regional seismic stations and four local stations in relation to the seismicity. However, the 15 km trial depth yielded the best epicenter results when comparing the horizontal dispersion of the epicenters over the transform fault along the five original depths investigated.

(iv) Mainshock Intensity at Jan Mayen Island

The consequences or degree of damage caused by the ground shaking at specific locations on the surface of the Earth is referred to as the local intensity caused by the earthquake. With little physical impact on nearby building structures, the staff at the Norwegian Armed Forces station located at OLONKIN CITY (https://jan.mayen.no/about-jan-mayen/) on Jan Mayer Island, which is about 30 km from the epicenter, woke up by the shaking and felt the building sway seconds after the mainshock. The only obvious repercussions were minor: a pen from a notice board in the corridor, a toothbrush from a shelf onto a mirror, and a little box of underwear that had been resting on the edge of a counter all dropped and the door of a cupboard opened. Nevertheless, no serious damage was done to equipment, materials, or buildings. The reports indicate an intensity of IV (Mercali), agreeing with the USGS ShakeMap predicted value (Figure S9; https://earthquake.usgs.gov/earthquakes/eventpage/us6000pxvx/shakemap/intensity). However, the earthquake's strong ground motion led to a prediction of Intensity 8 in the most northern west region of the island, where the Kjerulf Glacier is situated. This region is situated only 5.4 km from the epicenter with the USGS Ground Failure indicating chance to have landslide occurred in the area (Figure S10; https://earthquake.usgs.gov/earthquakes/eventpage/us6000pxvx/map?shakeMapIntensity=false&ground-failure-landslide=true).

Text S2. Local seismic velocity decrease at Jan Mayen Island

Since the seismic sensors represents a virtual source, local ambient seismic noise correlation has become a standard technique that enables the recovery of the structural response between two receivers recording noise. In the equatorial Atlantic, these environmental perturbations and changes of the local seismic noise have been investigated as the effect of the local seismicity [8] and the change in weather station of the year [9]. Previous author [10] suggested using seismic noise auto-correlations to study the environmental impact, while other authors [11] suggested using cross-correlation by several stations to recover the reflection response. In seismic noise research, stationary waves and resonance effects offer information about subsurface parameters such as layer thickness and material stiffness after strong earthquakes such as the effect in subduction zones [12]. Resonance effects arise when ambient noise vibrations interact with geological features, particularly barriers with high impedance differences, to produce standing wave patterns at certain frequencies. These resonances can be directly identified along the variation is lag time applied in the cross-correlation process of the seismic noise interferometry monitoring:

$$C_{CC}(t) = \sum_{\tau=\tau_0}^{\tau_0+T} u_1\,(t+\tau)u_2(\tau)$$

where $u_1$and $u_2$are the two seismic noises traces, t is the time lag applied, and T is the time window duration. In case of autocorrelation for the same seismic station is considered $u_1 = u_2$ [13].

The local seismic noise on the Jan Mayen Island was analyzed to study if the strong Mw 6.5 earthquake issued any environmental effects of mass movements have caused seismic resonances in local structure. We analyzed seismic data of the three local stations located approximately 20–32 km away (JNW, JNE, and JMI) from the mainshock using auto-correlation for each one of the three components (HHZ, HHN, HHE). We used the open source *eseis* toolbolx (https://rdrr.io/cran/eseis/)[14]. We study data recorded between March 1 at 00:00:00 and March 21 at 00:00:00, applying a time lag of 40s (-20 to +20) and a band-pass filter of 0.2-0.5 Hz [8]. We used time windows of 1 minute. *eseis* also estimate the relative velocity changes (dv/v) over time using the stretching method [15]. It is based on finding the relative time stretch that maximizes the similarity between the two signals that provide the fractional time stretch (∈) from the best match. dv/v can be obtained from the negative value of the best match ($\frac{dv}{v} = -\in$), and was calculated with results obtained by both JNW and JNE seismic station.

The results show a rapid velocity decrease of up to 7% in both the vertical (HHZ), north–south (HHN), and east–west (HHE) components (Figure 3 in maintext). The velocity reduction roughly began ~3 minutes and 25 seconds (~02:37:00 UTM) after the ground motion caused by the Mw 6.5 earthquake, and the resonance effect persisted for approximately 50 minutes. Thereafter, the dv/v returned to background noise levels as

before the earthquake (Figure 3a,b,c in maintext). The observed short time reduction in seismic velocity is consistent with changes in dynamic elasticity in shallow crustal materials. Normally, the velocity reduction caused by an earthquake remains for months or years [16,17,12], including effects produced by an oceanic transform fault earthquake [8]. However, our identified dynamic velocity decrease occurred for less than one hour. Several physical mechanisms may explain this phenomenon: 1.) Seismic waves produced by earthquakes, particularly surface waves, can damage near-surface materials by opening microcracks and delaminating mineral grains in rocks due to rapid dynamic stress propagation [18]. This mechanism reduces the elastic modules, and therefore the seismic velocity. 2.) Dynamic shaking in volcanic and glaciated areas can lead to increased pore fluid pressure in fractures or beneath the glacier. This can reduce effective stress and elastic rigidity. This feature can cause a noticeable drop in dv/v [19]; 3.) Glacial or surface ice coupling effects: Because the seismic stations occur close to the Kjerulf and Weyprecht glaciers, seismic waves from the strong mainshock may have amplified surface wave energy or altered near-surface stress conditions due to the ice-rock interface and surface material motions like landslide [20], resulting in an intensified dv/v response. These findings demonstrate the effectiveness of ambient seismic noise interferometry in detecting rapid, localized, and reversible changes in subsurface material properties following strong seismic events or possible landslide in the Jan Mayen Island

Text S3. Earthquake ground motion modeling

Ground motions across the study area were estimated using a DEM-driven implementation of the OpenQuake ground-motion framework (hazardlib)[21]. Source-to-site distances were approximated for every DEM cell from the relocated hypocenter (71.1935° N, 8.1905° W; 18 km depth below sea level), using a simplified finite-rupture correction with length from Mw following [22] for oceanic strike-slip faulting. Ground motion (PGA, PGV and SA at 0.2 s) was computed as a weighted combination of the [23] backbone ground-motion model, as regionalized within the ESHM20 logic tree by [24], and the referenced empirical model of [25] for Hawaii, using the branch weighting (0.21/0.79) adopted in the USGS ShakeMap solution for this event. A uniform Vs30 of 760 m/s was assumed, matching the site condition used in the USGS solution. Topographic amplification was superimposed following the frequency-scaled curvature (FSC) method [26], which predicts amplification on convex terrain and de-amplification on concave terrain from DEM curvature smoothed over a frequency-dependent wavelength. Fig. S13-S15 show the results.

Text S4. Mapping of the rock slope failure in the Kjerulf Glacier using MAXAR satellite images

High-resolution satellite images were used to observe and determine the landslide volume comparing the visible topography before and after the event. We used three

MAXAR panchromatic images [27] taken by WorldView-2 satellite with spatial resolution of 0.58 m and 8-bands. The images were taken in 2025/07/14 and 2024/06/14, with incidence angles 21°, 23° along the orbital track at 12:04:00 and 12:04:46 UTC, parallelly. The images were provided in primary GEOTIFF file format geocoded with the UTM29N coordinates system with 12 bits per pixel. Because the Jan Mayen Island is geographically located in the Arctic, the area is generally covered by snow and cloudy most of the time between November and the second half of March. Therefore, we needed to choose images from previous years, and the current year in time after the earthquake.

We analyzed the panchromatic MAXAR images using the open-source QGIS geographical information platform [28]. We compared the headscarp area at Kjerulf Glacier observed with the Sentinel2 L2A satellite (see Methodology), using the high-resolution frame taken by WorldView-2 satellite in 2025/07/14 with the image taken in 2024/06/14. The 50cm resolution image of 2025/07/14 presented a clear view of the collapsed volcanic wall on the south side of the Kjerulf Glacier compared to the 2024/06/14 (Figure S16a,b). The sloped surface shows apparent striations and flow-like patterns, indicating glacial ice moving downstream. A massive debris mass deposit was caused by a rock slope failure that issued from the facing wall. The debris cascaded down the steep slope and scattered across the glacier. The jagged, fragmented topography indicates a steep, highly fractured, and unstable rock wall. The irregular surface texture and dark tones indicate substantial erosion and rock debris accumulation. It is evident that there is a downward path from the wall to the glacier with the material fanning out and becoming more dispersed as it proceeds with more lengthy cracks and long ice breaking until it reaches the ice cliff along the shoreline. The effect is more clearly visible in a more recent MAXAR image taken on 2025-07-14 after the increase of the ice melting and higher visibility of the debris deposited in glacier surface (Figure S17a,b).

The highest position of the source area of the rock slope failure had a previous height elevation (H) of ~ 803 m above sea level from an 2 m ArcticDEM (https://livingatlas2.arcgis.com/arcticdemexplorer/) mosaic. The panchromatic MAXAR image of 2025-07-14 shows that the deposits of debris extend for a total area of ~0.88 km^2 across the glacier surface with a total perimeter of ~6.2 km, and a maximum straight ground length (L) of 2159 m away from the source, whereas the curvilinear runout distance following the topography is approximately 2265 m. Using a source elevation H (Fig. S18; Table S3), and the curvilinear runout distance yields a minimum H/L ratio of ~0.35 (reach angle ~19.5°), while the straight-line runout gives an H/L ratio of ~0.37 (reach angle ~20.4°). The most distant area covered by the debris deposit is located in the vicinity of the coast at an elevation of 0-20 m (https://topojanmayen.npolar.no/), indicating that the material moved downwards for ~800 m in elevation as a rock avalanche.

Text S5. Volume estimation of the material distributed by rock avalanche

We applied a DEM-based approach to estimate the rock volume. We used a co-registering 0.5 m WorldView (MAXAR) imagery to a 2 m ArcticDEM mosaic, which allowed a source area polygon to be mapped accurately on the DEM. We obtained a scene-wide translation (seed geometry) using AROSICS [29] by matching the DEM hillshade (rendered with illumination parameters matching the July 2025 WorldView image viewing geometry) to the WorldView scene image intensity within a stable-terrain mask (snow/ice, shadows, active slopes excluded). This produced a georeferencing-only shift with no resampling. We then refined local distortions by manually placing ground control points (GCPs) on stable, high-contrast features in QGIS *Georeferencer* [28] and applying a thin-plate spline (TPS) warp (cubic resampling), targeting local fidelity in the main area of interest (AOI).

The accuracy of the TPS-warped WorldView sub-scene was evaluated with AROSICS using the same stable-terrain mask and AOI constraint. From 30 raw tie-points, we applied quality gates (structural similarity index measure (SSIM) ≥ 0.10, absolute shift ≤ 8 m) and robust median-absolute-deviation (MAD) filtering (k = 3.5), retaining n = 12 high-quality, well-distributed tie-points. The resulting radial (planimetric) root mean square error $RMSE_r$ was 0.75 m (95% National Standard for Spatial Data Accuracy (NSSDA) = 1.29 m; bootstrap 95% confidence intervals (CIs) of $RMSE_r$ = 0.65–0.84 m), with small residual bias of (dx) 0.05 and (dy) 0.52 m. A more permissive (less strict) filter (SSIM ≥ 0.03, absolute shift ≤ 15 m, MAD k = 8) yielded n = 22 tie-points and a RMSEr of 0.76 m (95% NSSDA = 1.31 m, bootstrap 95% CIs = 0.67–0.86 m), confirming the metrics are robust to reasonable changes in the degree of tie-point filtering.

Subsequently, we estimated the source area volume using the Sloping Local Base Level (SLBL) method [30], with the 2 m ArcticDEM (https://livingatlas2.arcgis.com/arcticdemexplorer/) mosaic and the mapped source area as input. The method iteratively constructs a smooth basal surface beneath a delimiting polygon, with lowering limited to cells above a locally defined surface (base level') and integrates the thickness values (DEM minus modelled surface) to obtain a volume. The degree of basal curvature – and hence volume – is strongly controlled by the tolerance (C). We first estimated C automatically using pySLBL (https://github.com/ngu/pySLBL) from a centerline profile oriented along the mean slope direction:

$$C = 2 \cdot \left(1 - \sqrt{2}\right) \cdot \Delta z \cdot \left(\frac{x^2}{l^2}\right)$$

where Δz is the relief along the profile, l is its horizontal length, and x is the DEM cell size. We then modelled surfaces using bracketing tolerances and retained those that were geologically plausible based on visual inspection of 3D scenes and along topographic profiles (Figure S19) and ArcticDEM hillshade mosaic from 2 m used (Figure S20) to report a likely volume range for the failed source area (Table S4).

Text S6. Evaluation of potential tsunami generation following the rock avalanche

Because the rock avalanche reached the coastline at the Kjerulf Glacier, we investigated whether it generated a tsunami. We analyzed relative sea-level records from the Ittoqqortoormiit tide gauge instrument located in Scoresbysund, East Greenland (70.483º latitude and -21.967º longitude; https://www.ioc-sealevelmonitoring.org/station.php?code=scor), closest sea-level station to Jan Mayen Island (~800 km away). We examined the time series comparing from 7 days pre- to 7 days post-earthquake (28/02/2025-21/03/2025). Our goal to observe whether anomalous oscillations occur in first hours after the earthquake origin time (10/03/2025 at 02:33:15.8 UTC). We observed regular daily tidal variability of 0.3-0.5 m amplitude, without any abrupt perturbations or high-frequency wave trains over the 7 days both before and 7 days after the earthquake (Fig. S25-S27). The first 10 hours after the earthquake there is a maximum oscillation of 0.10-0.15 m, sea level oscillation. Therefore, this absence of a clear signal indicates that no regional tsunami was generated.

Text S7. Pre-earthquake rock failures at Kjerulf Glacier

Searching the satellite data revealed a small number of small rock failures predating the Mw 6.5 earthquake of 2025. Events occurred in October 2017, September 2019, October 2023 and October 2024 at Kjerulf Glacier, including some mass movements on the rock wall. In Figures S28-S31 we show these events in Sentinel-2 images.

The first one occurred in October 2017 at ~670 m south (71.11931 latitude, -8.13982 longitude) of the 2025 large rock failure, identified by the Sentinel-2 imagery took in October 19 (Figure S28). The visibility of debris is difficult but its possible to see the material cascading in south wall of the glacier. Even the resolution of the imagery, its possible to infer the very small size of the failure.

The second failure occurred in September, 2019 (Figure S29) at 71.120602 latitude and -8.141614 longitude, ~580 m south of the large rock failure that occurred in 2025, a few dozen meter north of the 2017 failure. From the image date, the failure appears to have occurred between September 9 to September 29 with a clear visibility of the small failure with imagery from September 29 (Figure S29). Neither the NSNN, GCMT, or WILBER3 catalogue showed any earthquake occurring at the time of the 2017 and 2019 failures.

A third event occurred in October 2023 again on the south side wall of the Kjerulf Glacier (~71.13104 latitude and -8.15345 longitude; Figure S30), ~400 m north of the large rock failure that occurred in 2025. From the images available, the failure appears to have occurred after May 2023, with a clearer visibility in the later image of 02 October 2023 (Figure S30). Neither the NSNN, GCMT, or WILBER3 catalogues show earthquakes

with magnitudes greater than 4 in 2023 near the Kjerulf area. These three examples imply that they might have originated from a consistent supply of rock.

Another small rock-slope failure occurred on the south wall of Kjerulf Glacier in October 2024, preceding the larger rock failure triggered by the earthquake on March 10, 2025 by a few months. This small rock-slope failure can be identified through Sentinel-2 optical imagery, with the October 19 and 20, 2024 (Figure S31a) images showing a newly exposed rock face and the debris on the south of the Kjerulf Glacier, located at 71.12712ºN and 8.147069ºW and, hence, just north of the 2025 landslide and thus spatially related to the rock slope failure in March 2025. During the following winter months, Sentinel-2 optical data were unavailable due to polar night and persistent snow cover. When optical conditions improved, the next available Sentinel-2 optical imagery were from February 8, 2025 and February 18 (Figure S31b) with new deposits on the glacier being absent compared to the previous observations in October 20. The Sentinel-2 image showed that although the glacier remained largely snow-covered, large boulders (~7 m) were visible on the surface, corresponding to the October 2024 event. Since the Sentinel-2 or other optical satellite were not cloud free between February 18 and March 10, we cannot assess the volume of this pre-earthquake failure precisely, but the source area suggests a slide volume smaller than the March 2025 failure (Figure S31b). Neither the NSNN, GCMT, or WILBER3 catalogues show earthquakes near the Kjerulf area in October 2024 to February 2025 period.

Rarely do intact rock slopes failure abruptly; instead, they undergo gradual deformation over time, primarily driven by gravitational forces acting on the rock mass [31]. Their evolution is governed by both static conditioning factors (e.g. lithology, structural discontinuities, and slope geometry) and dynamic factors that vary over time (e.g. erosion, groundwater fluctuations, weathering, and permafrost degradation) [32,33]. These dynamic processes progressively reduce shear strength and increase internal strain, conditioning the slope toward failure which is called progressive rock slope failure [34]. Such a slope that has favourable conditions for failure and has weakened progressively in time is more susceptible to failure during external extreme events. In the case of Kjerulf Glacier, long-term weakening due to erosion and permafrost degradation likely preconditioned the basaltic stratified volcanic slope on the south wall and the March 10, 2025 Mw 6.5 earthquake acted as the ultimate trigger that released the remaining unstable rock mass. The slope is unlikely to be in equilibrium today, and additional failures may be expected. Slope parallel depressions filled with snow suggest new development of fractures.

Text S8. Observation of an ice calving in Weyprecht Glacier from InSAR Sentinel 1 and MAXAR images

Comparing the Sentinel-1 descending frame 355 in path 67, taken on February 26, 2025, at 07:07:24 and March 10, 2025, at 07:07:42 (approximately 4 and a half hours

after the Mw 6.5 earthquake), we observed a glacier calving event at the edge of the Weyprecht Glacier (Figure S32). We identified high scattering material offshore on a Sentinel-1 picture taken on March 10, consistent with detached glacial ice that is flowing into the sea. To confirm, we compared the high-resolution MAXAR optical image [27] acquired on July 14, 2025, with the June 14, 2024 (Figure S33). The MAXAR images demonstrate a significant recession of the Weyprecht Glacier front edge, exhibiting structural instability and exposed crevasse fields indicative of recent ice separation that fell into the sea after the Mw 6.5 earthquake from the Sentinel-1 images taken on March 10. Figure S34-S35 show a clear observation pre- and post-earthquake from local pictures took in front of the glacier. We tried to process the InSAR coherence between the two interferometric frames using the GMTSAR package [35], but the coherence was insufficient for displacement measurements because the whole island was covered by snow on March 10. Nevertheless, the enhanced offshore radar backscatter observed in the post-earthquake Sentinel-1 image is consistent with detached ice blocks produced by glacier calving occurred at Weyprecht Glacier, with our interpretation independently confirmed by the post-earthquake MAXAR imagery and local pictures.

Text S9. Relative relocation of three other earthquakes that occurred close to Kjerulf Glacier and comparison with Landsat satellite imagens

To determine whether large-scale rock failures like the 2025 Mw 6.5 earthquake occurred close to Kjerulf Glacier, we evaluated three previous earthquakes (1997-2004) that occurred less than 10 kilometers from the collapsed area at Kjerulf. We then compared them to Landsat satellite pictures taken before and after the earthquake to see if other rock failures had occurred in the past.

We used a relative relocation approach [36] to improve the epicentral positions of three additional earthquakes that occurred near Kjerulf Glacier on December 13, 1997, at 07:02:03 (UTC) with Mw 5.6 (GCMT), May 21, 2000, at 19:58:47 Mw 6.0 (GCMT), and April 14, 2004, at 23:07:39 Mw 5.9 (GCMT). The mainshock epicenter was fixed to the previously determined 2025 Mw 6.5 location using the single-station approach, while the epicenter coordinates of the relocated events were obtained from the USGS (https://earthquake.usgs.gov/) catalog, as these were the closest to the single-station location and provided the most consistent reference frame. Differential arrival times were calculated by cross-correlating Rayleigh waves filtered between 0.02-0.04 Hz to reduce dispersion and improve signal-to-noise. Every target earthquake was linked to an empirical Green's function (EGF) event, with relative moments calculated as the ratio of the cross-correlation peak to the EGF autocorrelation at zero lag. We maintained only high-quality correlations ($r>0.7$) and used an L1-norm cosine fit to determine relative azimuth and distance, assuming a group velocity of 3.7 km s^{-1}, which is consistent in young oceanic lithosphere [37]. Uncertainties were measured using bootstrap resampling with a nominal 1 s error [38]. When the reference EGF proved too far, the method was

repeated with a closer event. This method uses waveform similarity to obtain kilometer-scale precision in relative locations and resolve the spatial organization of recurring seismicity.

We obtained result with good fitting in the observed/synthetic waveform relation of the long-period Rayleigh wave on the long period (LHZ) vertical component in all three relocated events (Figure S36-S38). The results indicated relative epicenters placed 2.9-29.6 km away from the mainshock, and 6.9-9.0 km away from the rock slope failure location at the south side of the Kjerulf Glacier (Figure S39-S41), with one of them with epicenter located even closer than the epicenter of the 2025 Mw 6.5 that triggered the rock slope failure. Relocated coordinates are presented on Table S5.

We analyzed six Landsat satellite images [39] captured between 1996 and 2004 to observe if other rock failures occurred with the visible topography demands comparing imagens pre- and post-earthquake for the three events that occurred in 1997, 2000, and 2004. For the 1997 Mw 5.6 earthquake, we used two Landsat-5 imagens taken in 1996/09/11 and 1998/08/02 with incidence angle of 23º and 37º and spatial resolution of 30 m. We used four pictures of the Landsat-7 satellite to check the other two earthquakes, being two imagens taken in 1999/09/21 (20º incidence angle) and 2001/10/21 (8º incidence angle) to the 2000 Mw 6.0 earthquake, while we used to the 2004 Mw 5.9 event two pictures of 2002/09/04 and 2004/08/10, with 26º and 34º incidence angles. Six images were provided on EOS LandViewer (https://eos.com/landviewer) in primary GEOTIFF file format geocoded with the WGS84 coordinates system with 8 bits per pixel.

Comparison between pre- and post-event Landsat imagery from the 13 December 1997 (Mw 5.6; Figure S42-S43), 21 May 2000 (Mw 6.0; Figure S44-S45), and 14 April 2004 (Mw 5.9; Figure S46-S47) earthquakes found no signs of large-scale rock avalanches or widespread debris accumulations equivalent to those caused by the 2025 Mw 6.5 event. We did not get teleseismic seismic data to relocate another older earthquake that occurred at 04:01:49 UTC on 13 December 1988 Mw 6.0 earthquake. However, Landsat imagery do not show any debris deposit comparing pre- and post-earthquake (Figure S48-S49). Although the previous earthquakes presented weaker magnitudes, two had epicentral distances similar or 2 km shorter than the 2025 event, meaning that they might have caused similar levels of ground shaking. Despite the gap in spatial resolution between Landsat (30 m) and MAXAR (0.5 m) satellite images, the lack of identifiable large debris deposits in previous records suggests that no analogous rock failures happened previously with the same or similar debris material distributed over the glacier surface. These findings indicate that no earthquake in the last four decades, at least, has caused at the Kjerulf Glacier on the scale of the rock avalanche created by the 2025 Mw 6.5 event.

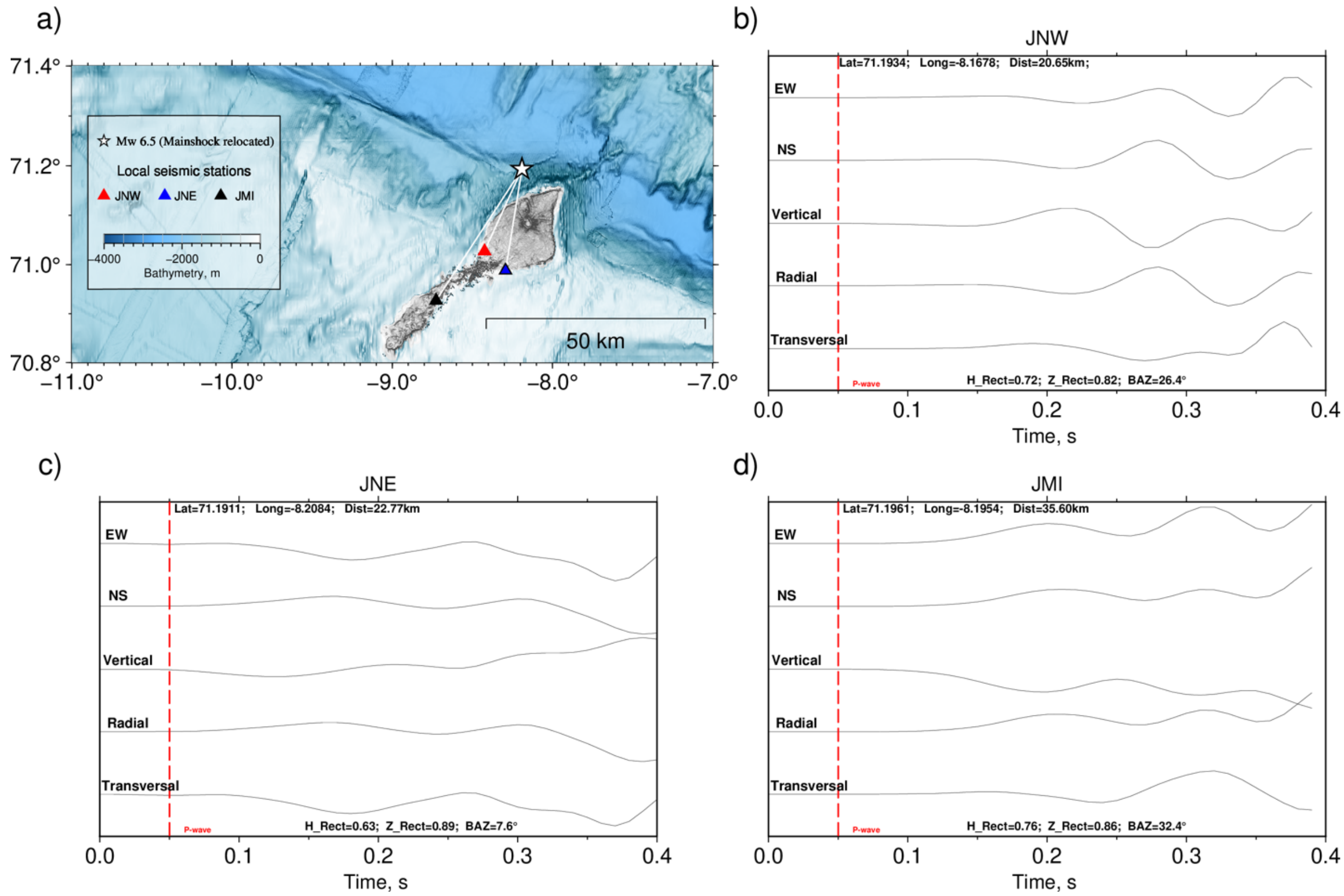

Figure S1 – Epicenter location of the mainshock from the P wave rectilinearity approach. a) Bathymetric/topographic map of the Jan Mayen Island area. Red/Blue/Black triangles indicate the location of the JNW/JNE/JMI seismic stations. White star refer to the relocated epicenter. b) P wave seismogram of the East-West (EW), North-South (NS), Vertical, Radial, and Transversal components referent to the JNW seismic station. The values of the horizontal (H_rect) and vertical (V_rect) rectilinearity are shown in the bottom side of the figure, together the back-azimuth (BAZ) direction obtained. On upper side: latitude and longitude of the individual location obtained by that station, and the distance between the station and the obtained epicenter location. c) and d) indicate the same information provided by b), but referent to the JNE and JMI local seismic stations.

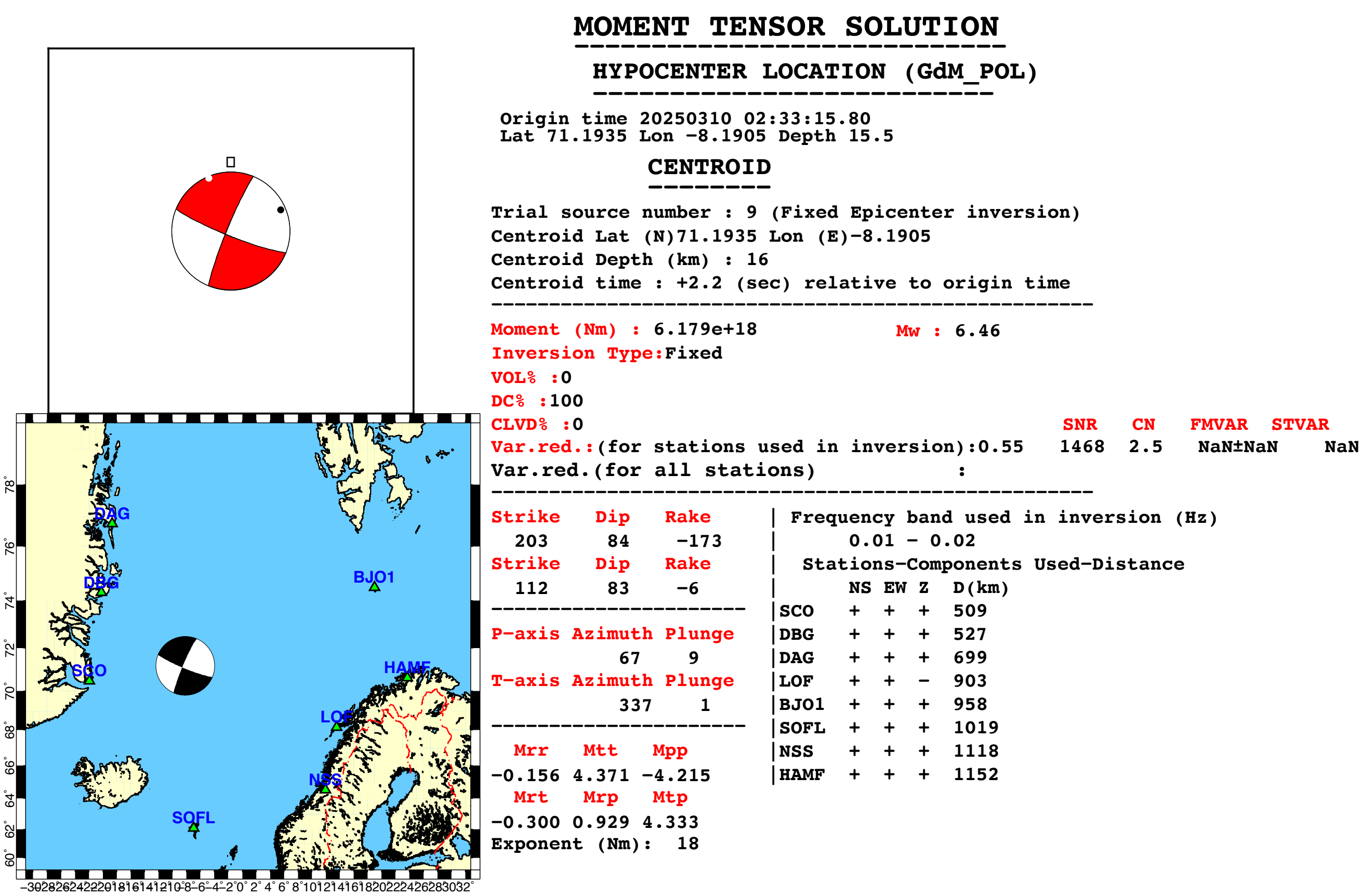

Figure S2 – Moment tensor solution results obtained by ISOLA. Focal mechanism fixed in inversion from GCMT solution is presented on upper west side plot, with the map showing the regional station locations presented on map in bottom west side.

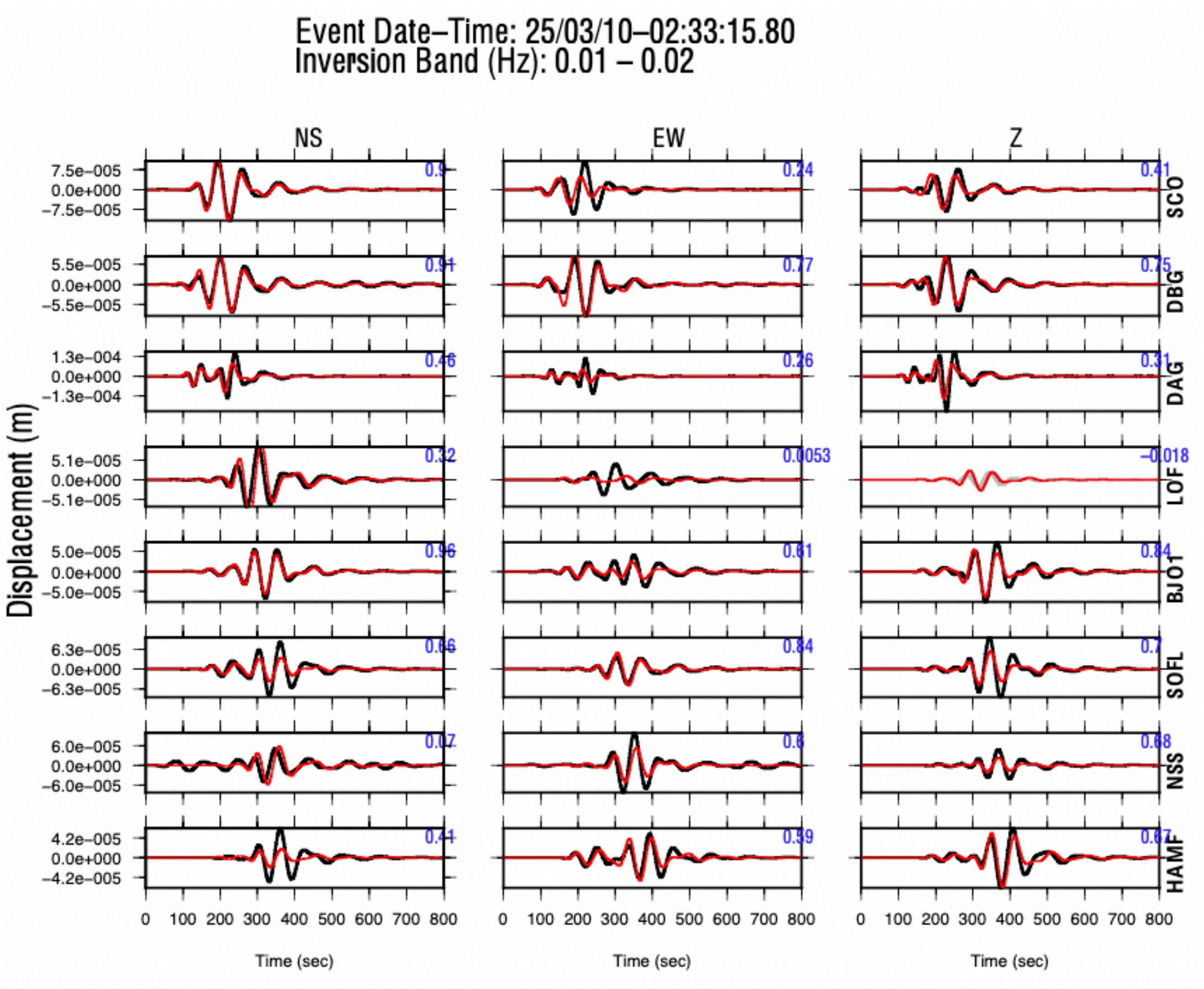

Figure S3 - Waveforms (black) and their synthetics (red) obtained with the best-fitting source position in the 10 March 2025 Jan Mayen earthquake analyzed on ISOLA. The modeled solution has a 16 km depth, varied reduction = 0.55, and condition number 2.5. Data plotted in gray on the vertical component of the LOF station were not used in the inversion due to bad fitness. Numbers at the top-right corner of each panel are the variance reduction for a particular component.

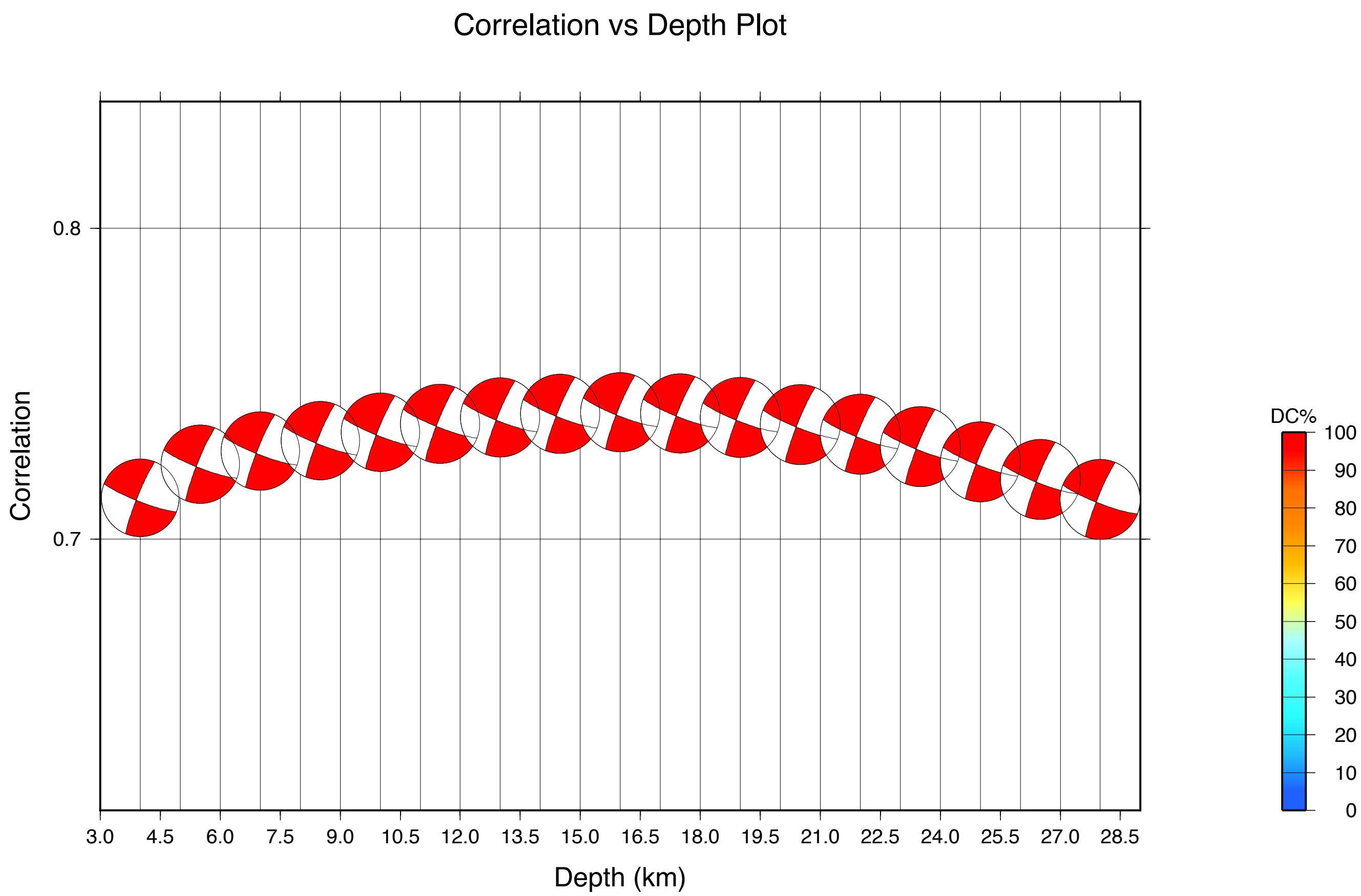

Figure S4 - Correlation vs Depth for earthquake 10 March 2025 Mw 6.5 event, using frequency range 0.01-0.02 Hz. Higher correlation is located at 16 km depth, with a steeper decrease of the correlation in following and previous depths.

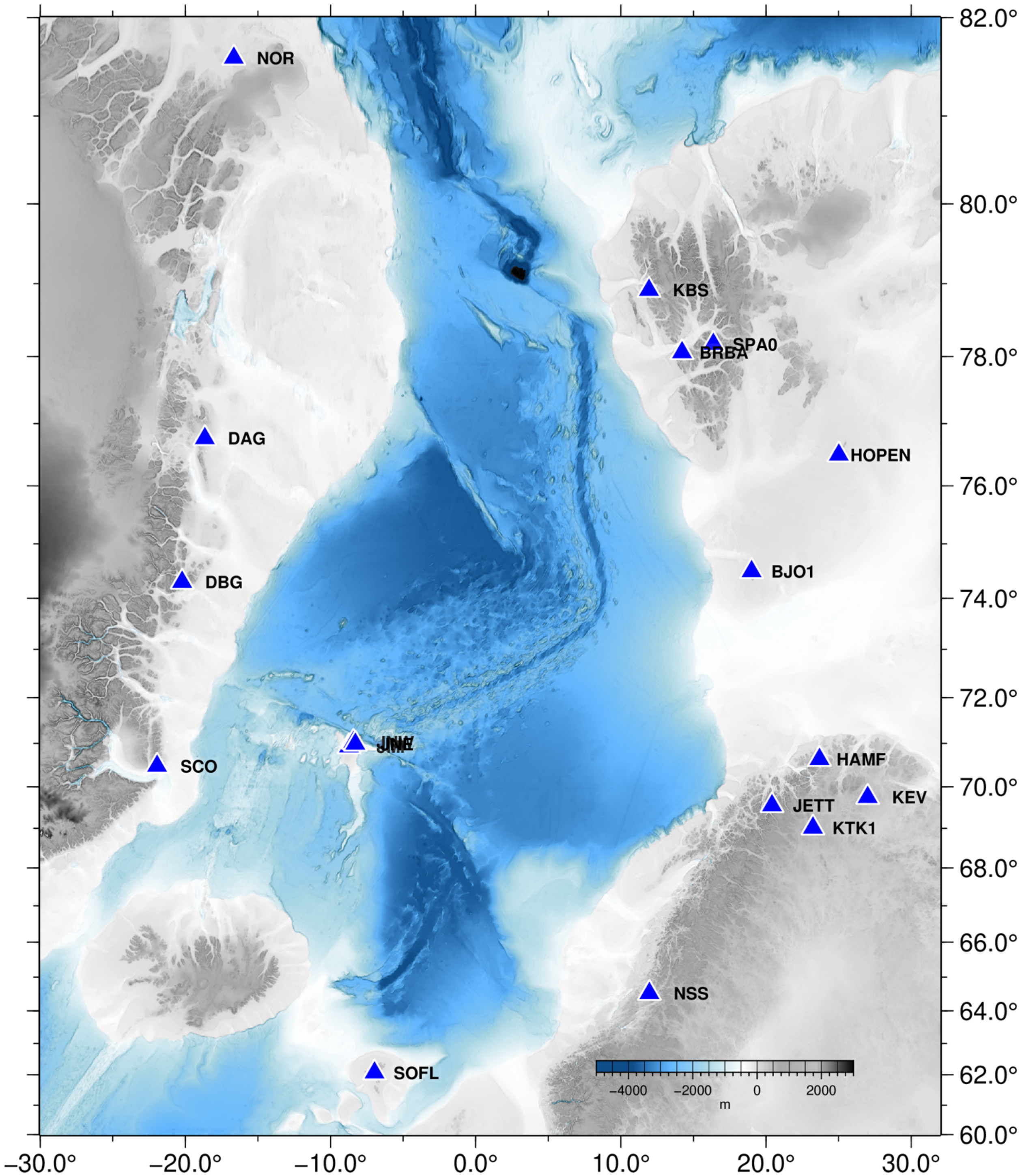

Figure S5 – Geographical map with the regional and local seismic stations (blue triangle) used in epicenter relocation of the aftershock seismicity.

a)

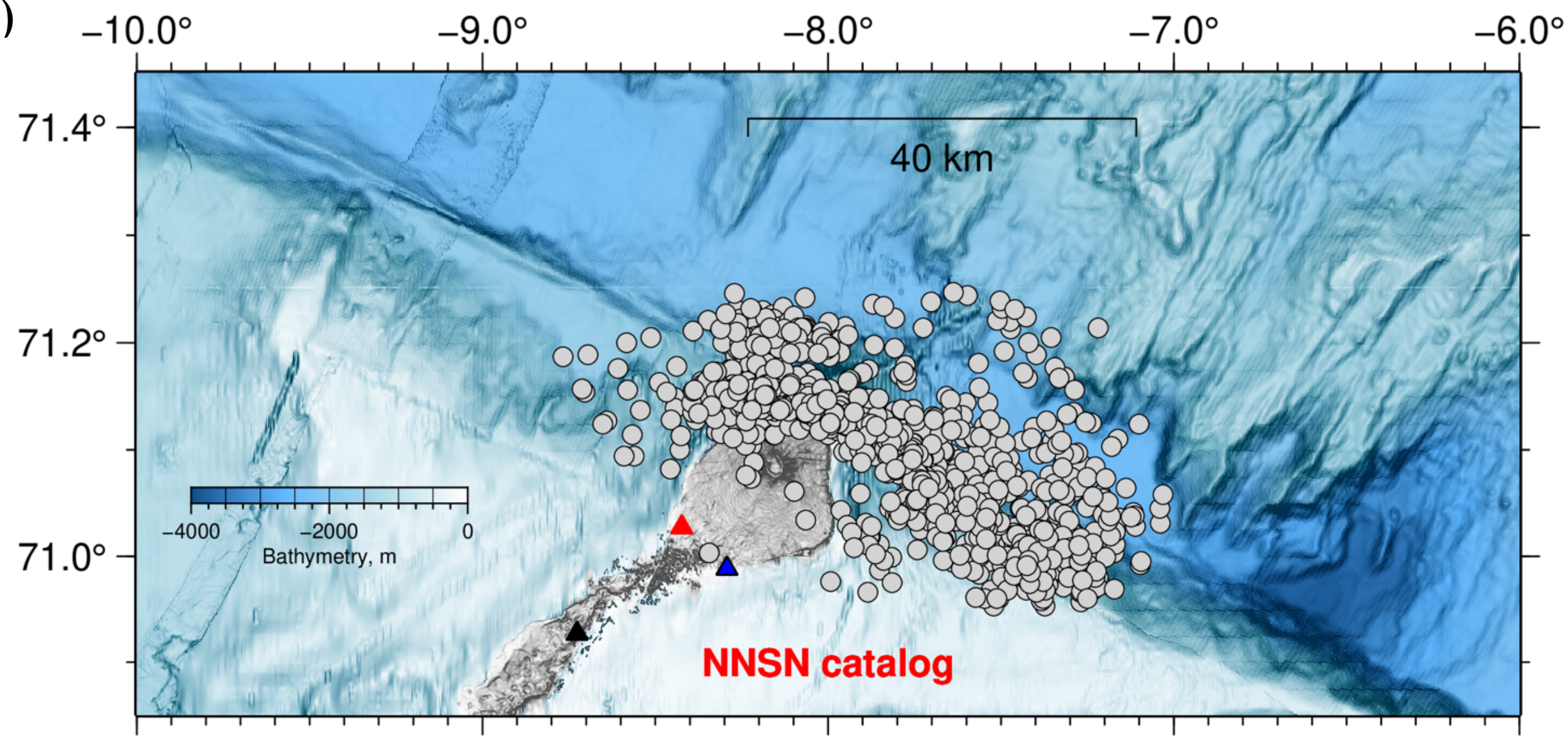

b)

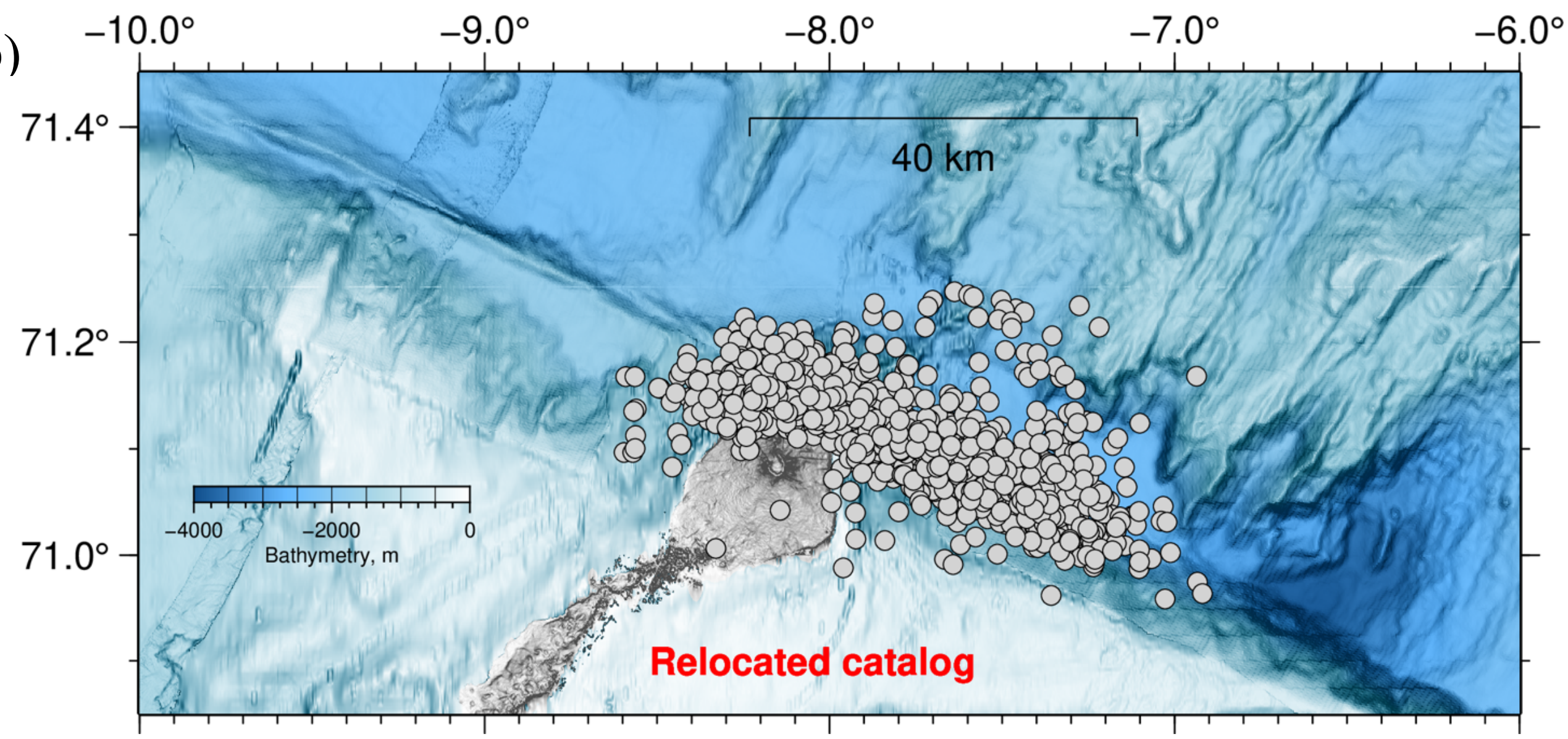

Figure S6 – Epicenter location of the aftershock seismicity around the Jan Mayen Island. Comparison of the initial epicenter location presented by gray circles in a), with the relocated epicenters distribution in b).

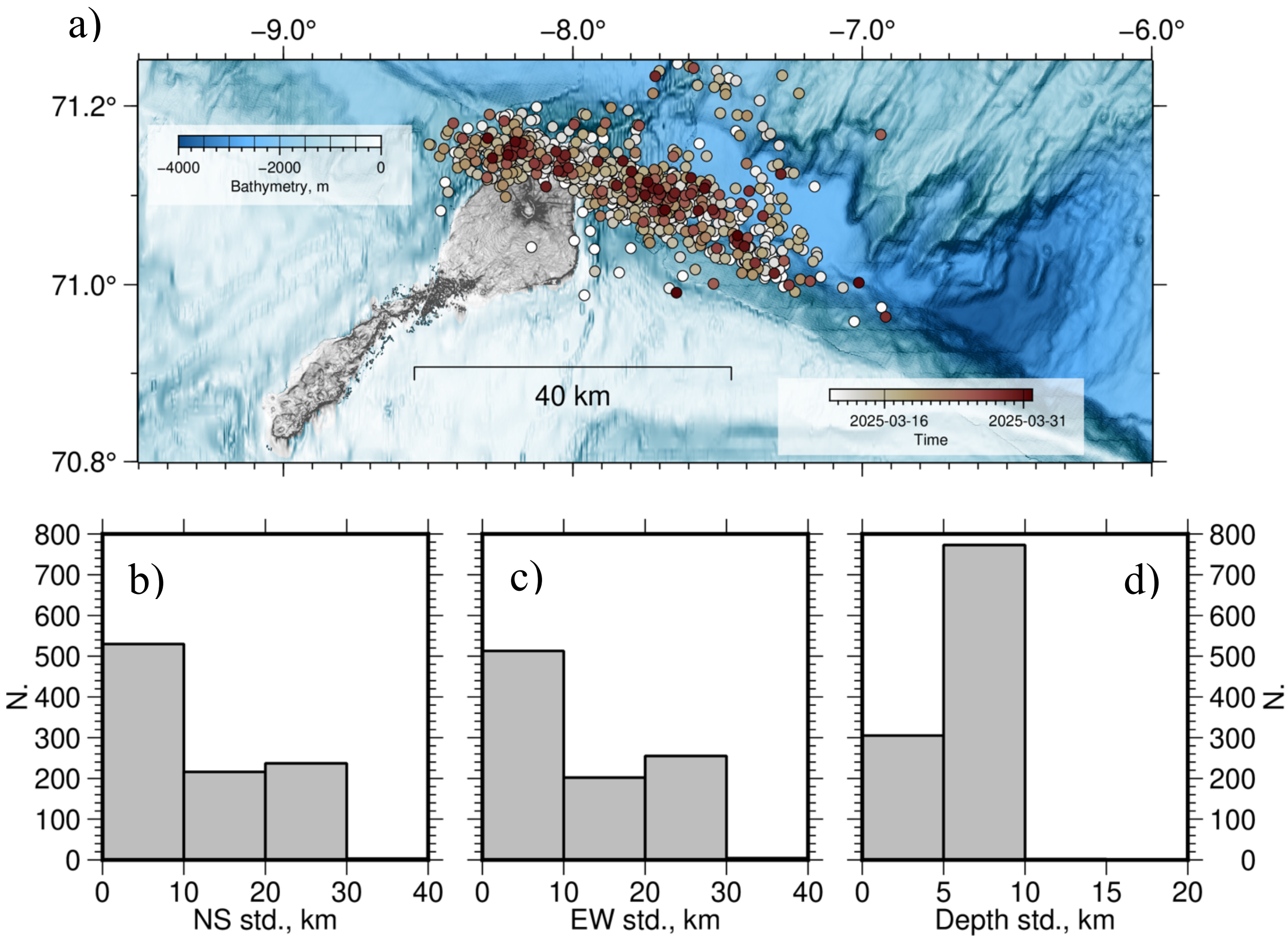

Figure S7 – Spatial-temporal epicenter distribution of the relocated aftershock seismicity. a) Map of the spatial distribution of the epicenter over the Jan Mayen Island and Jan Mayen Transform Fault. b/c/d shows the distribution of the standard deviation with respect to the relocations on North-South/East-West/Depth directions, consecutively.

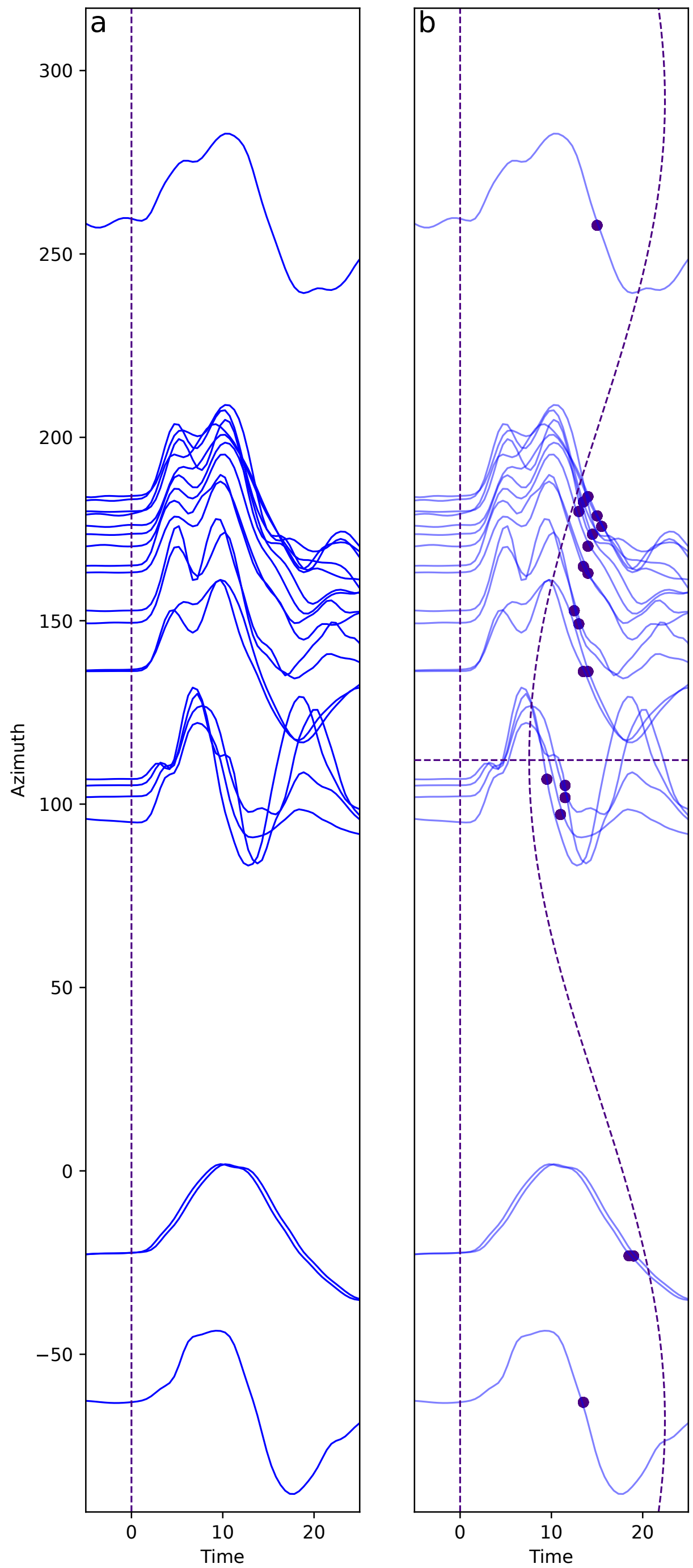

Figure S8 – a) Normalized, integrated displacement waveforms of selected P wave pulses, aligned to their onsets (indigo dashed line). b) The apparent rupture durations (dots) are estimated from P pulses. The first, vertical dashed line indicates the rupture onset, and the second, curved one the modeled azimuthal pattern of apparent rupture durations, assuming a pure unilateral rupture towards the inferred azimuth (112°, horizontal dashed line), a rupture velocity of 3 km/s, a rupture length of 45 km and P wave velocity of 6.04 km/s [40].

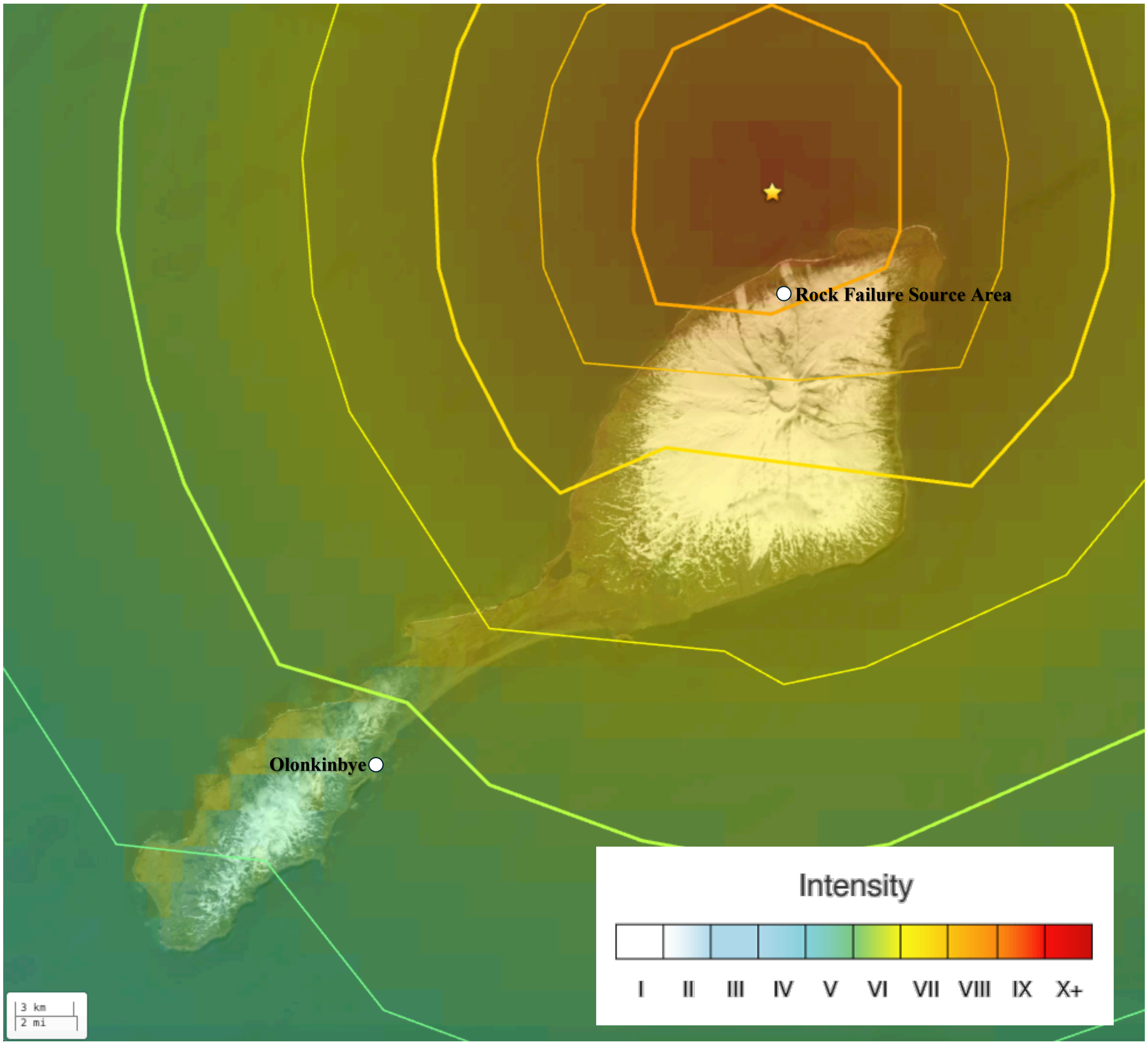

Figure S9 - USGS Ground Shaking Intensity Distribution from the 2025 Mw 6.5 Jan Mayen mainshock [us600pxvx] – The map illustrates the spatial extent of the intensity values derived from seismic data presented in color lines. The higher intensity with level VIII is presented in red zone around the epicenter (orange star) and the rock failure source area in north of the Jan Mayen Island, while the less predicted impact on local infrastructure of the Norwegian Armed Forces camp basin located in Olonkinbye.

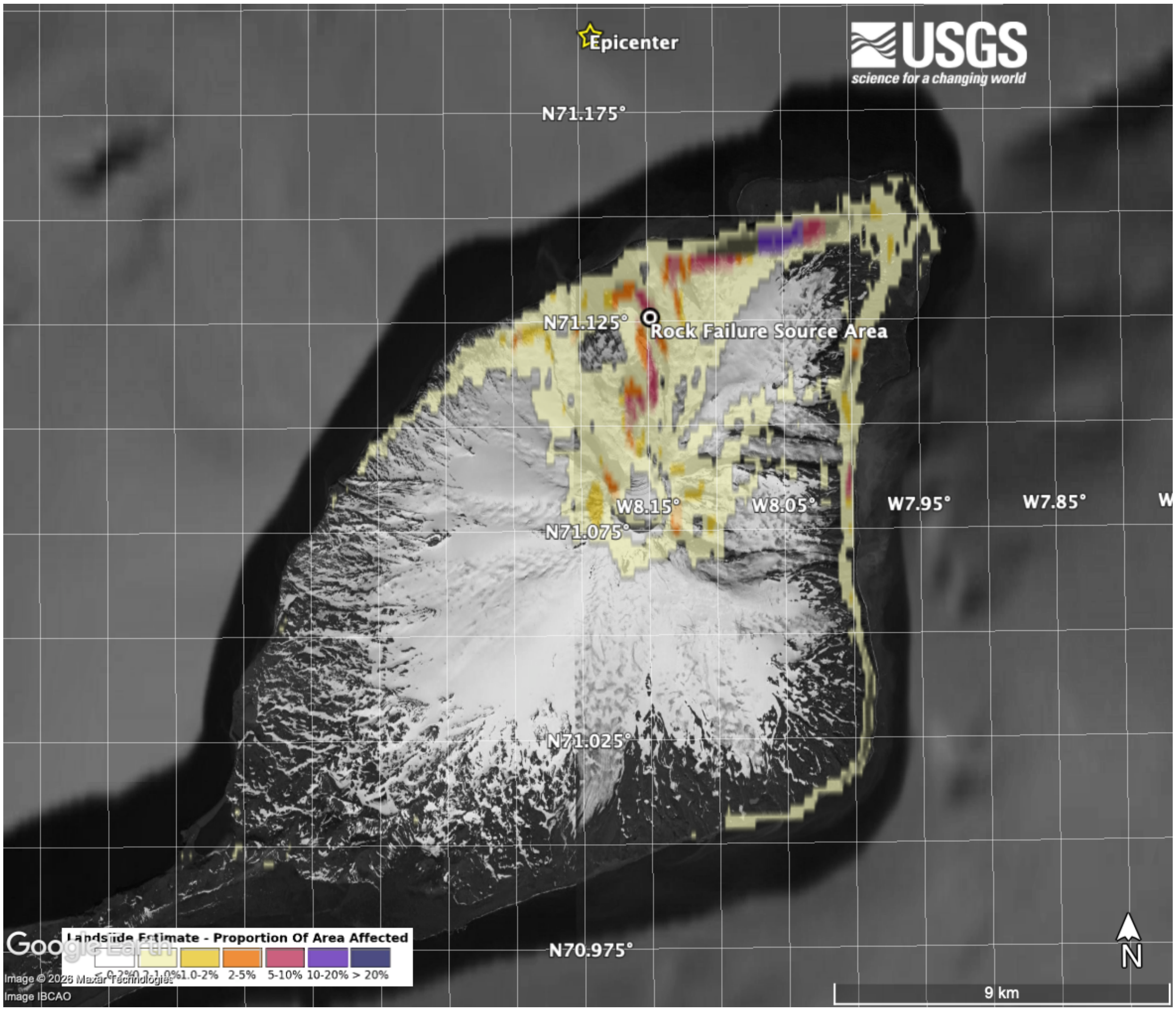

Figure S10 - USGS Predicted Ground Failure and Landslide Map for [us600pxvx] – Colors indicate the estimated proportion of area affected by landsliding. The Kjerulf Glacier rock-failure source area is presented by white circle, with the prediction indicating a chance of landslide form 5-10%. Earthquake epicenter is presented by the yellow symbol.

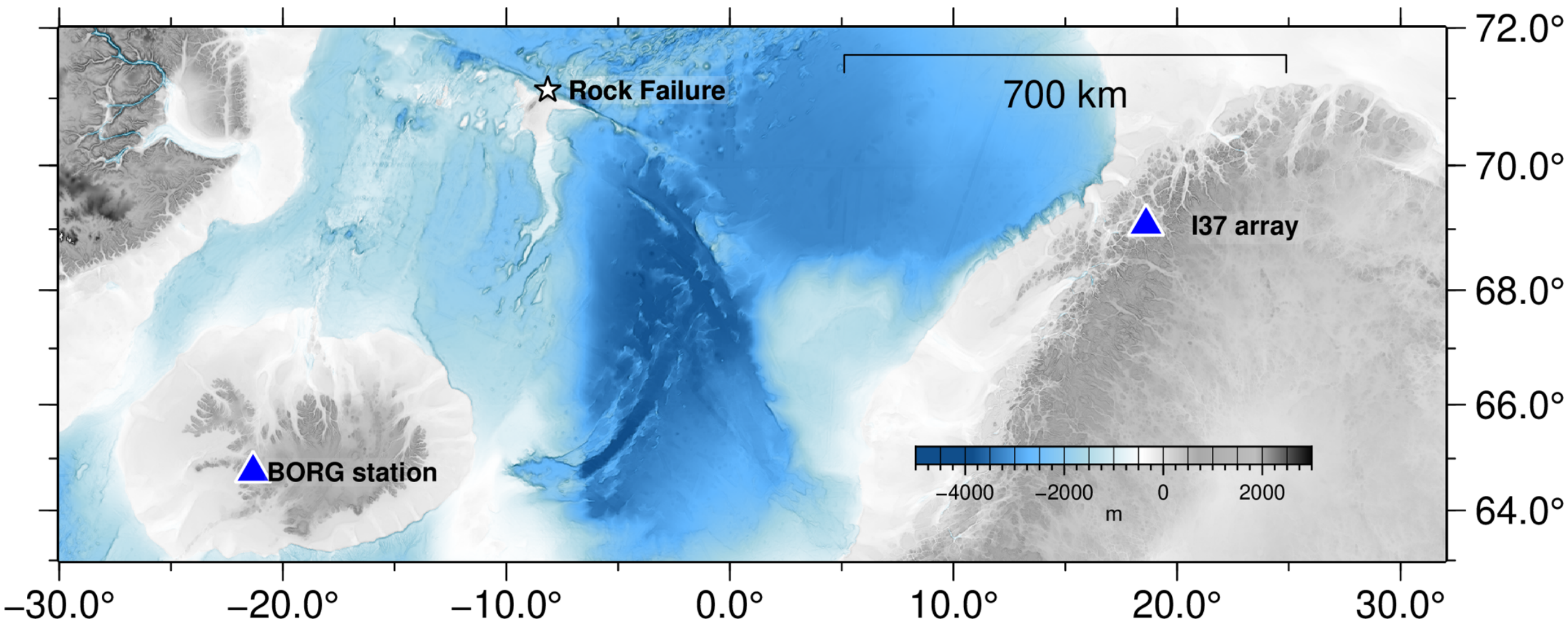

Figure S11 – Overview showing the location of the Kjerulf Glacier rock failure (black star), together with the I37 infrasound array in Norway, and the BORG single infrasound sensor located in Iceland. Both recorded the atmospheric acoustic signal generated by the rock failure.

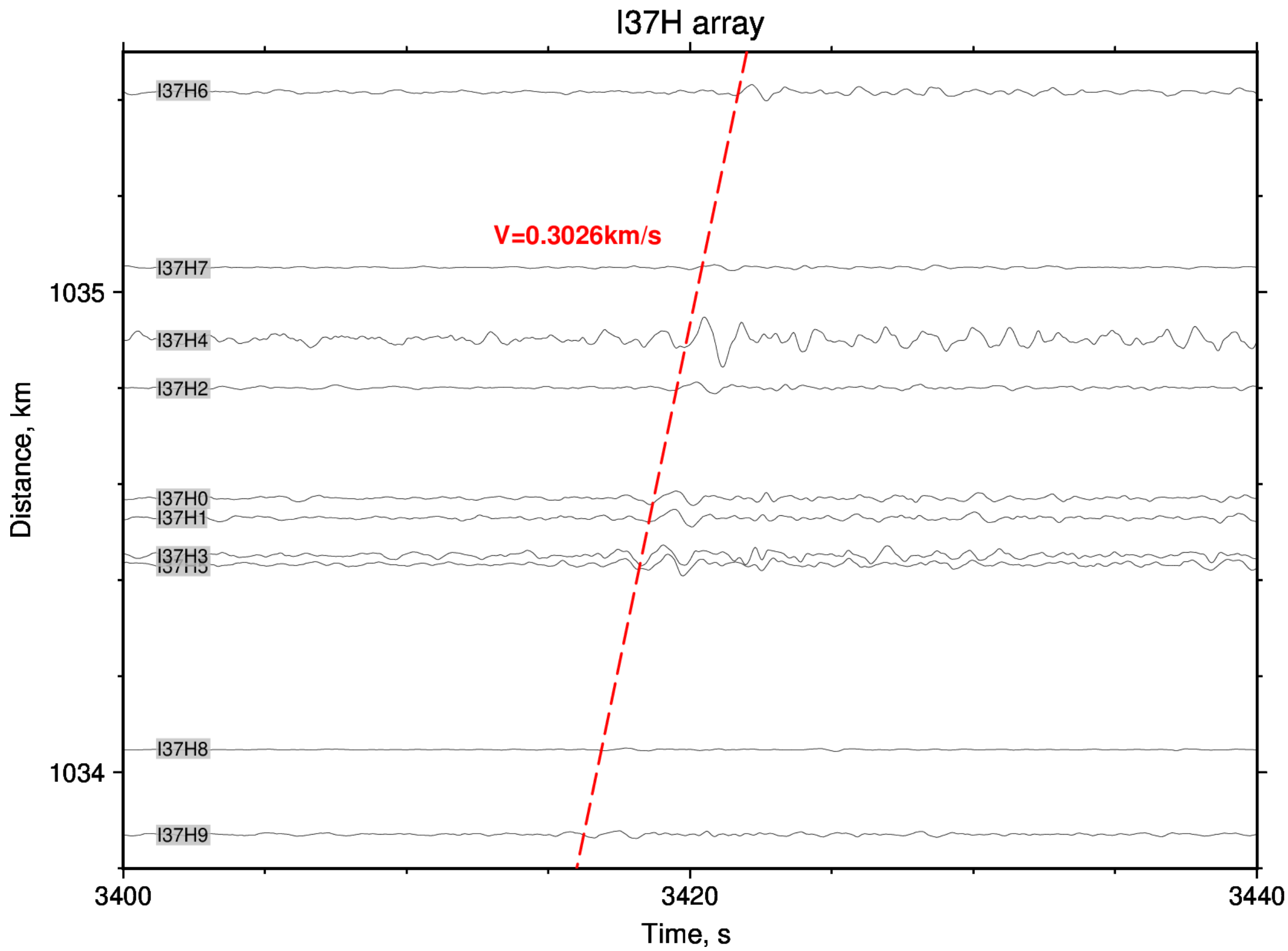

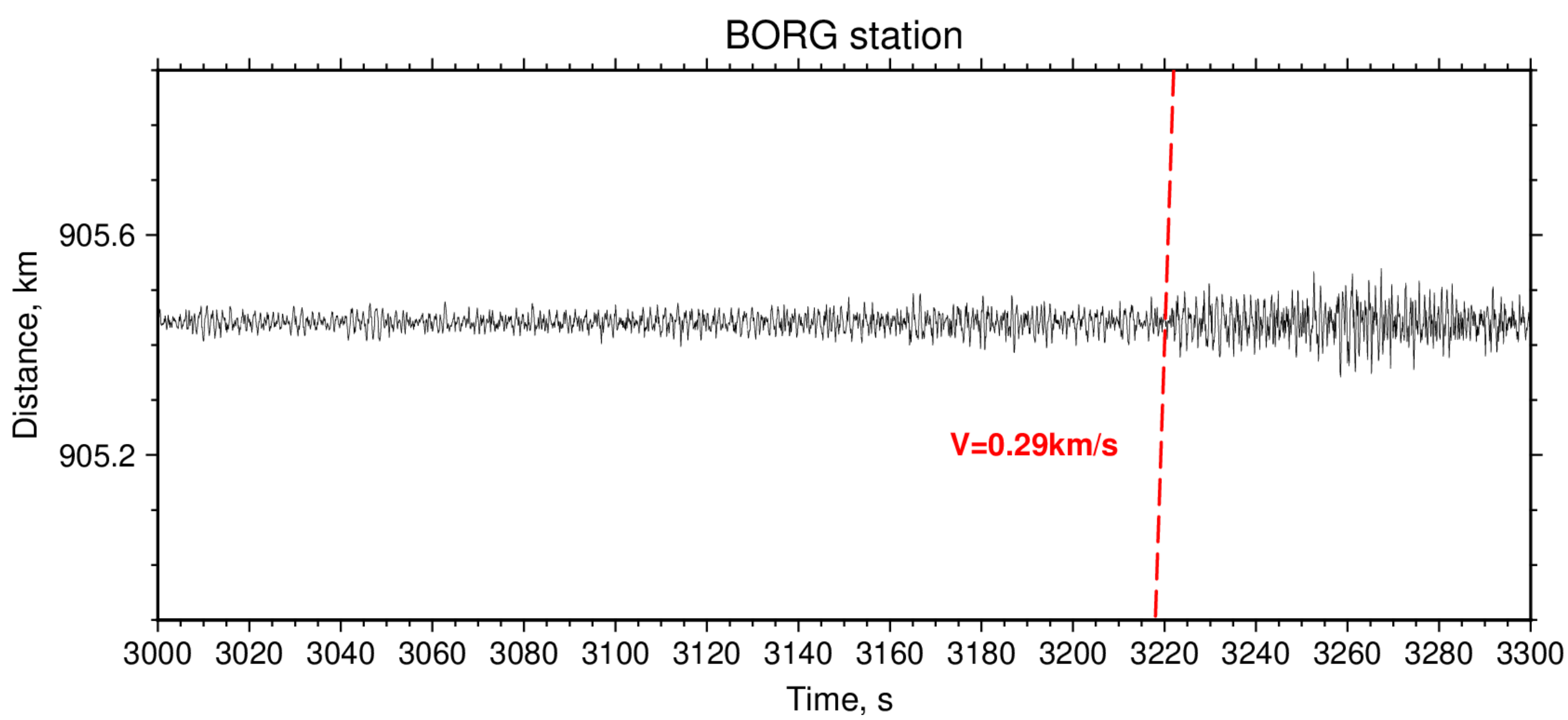

Figure S12 – Epicentral distance versus travel time of the infrasound signals plotted from the 0.6-4.0 Hz bandpass filter applied. Upper plot: record section of the I37H array located in Norway. Red dashed line mark identifiable first arrivals of the rock slope failure generated signals, with the velocity calculated from the complete stacking. Bottom plot: like previous one, but referent to the single BORG infrasound sensor in Iceland.

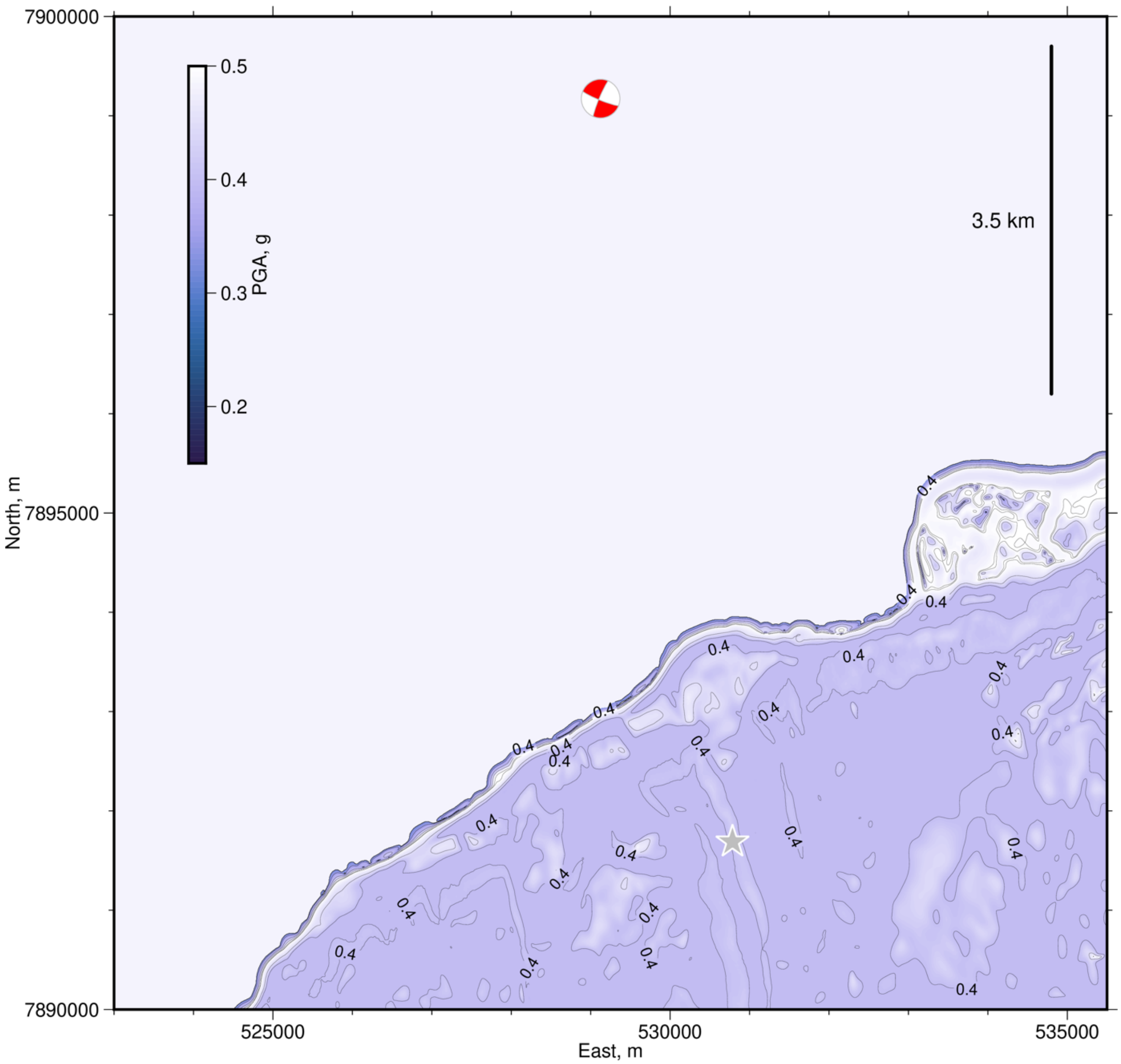

Figure S13 - Modeled peak ground acceleration (PGA) for the 10 March 2025 Mw 6.5 Jan Mayen earthquake. Contours and colors represent our modeled PGA (g). The focal mechanism (red beach ball) marks the mainshock epicenter, and the gray star indicates the Kjerulf Glacier rock failure source area. The modeled PGA at the rock-failure source is approximately 0.45 g.

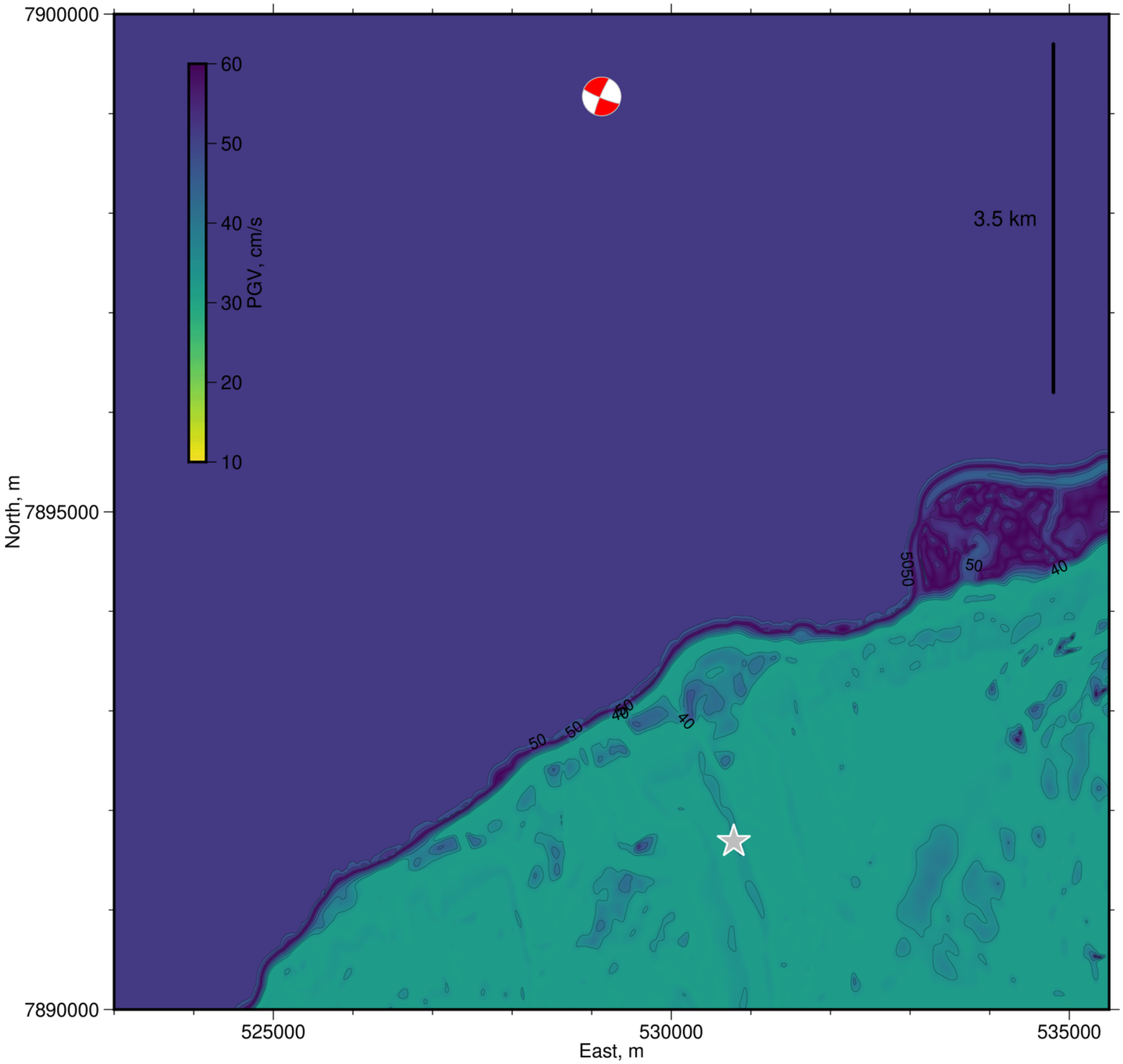

Figure S14 - Modeled peak ground velocity (PGV) for the 10 March 2025 Mw 6.5 Jan Mayen earthquake. Contours and colors represent our modeled PGV (g). The focal mechanism (red beach ball) marks the mainshock epicenter, and the gray star indicates the Kjerulf Glacier rock failure source area. The modeled PGV at the rock-failure source is approximately 42 cm/s.

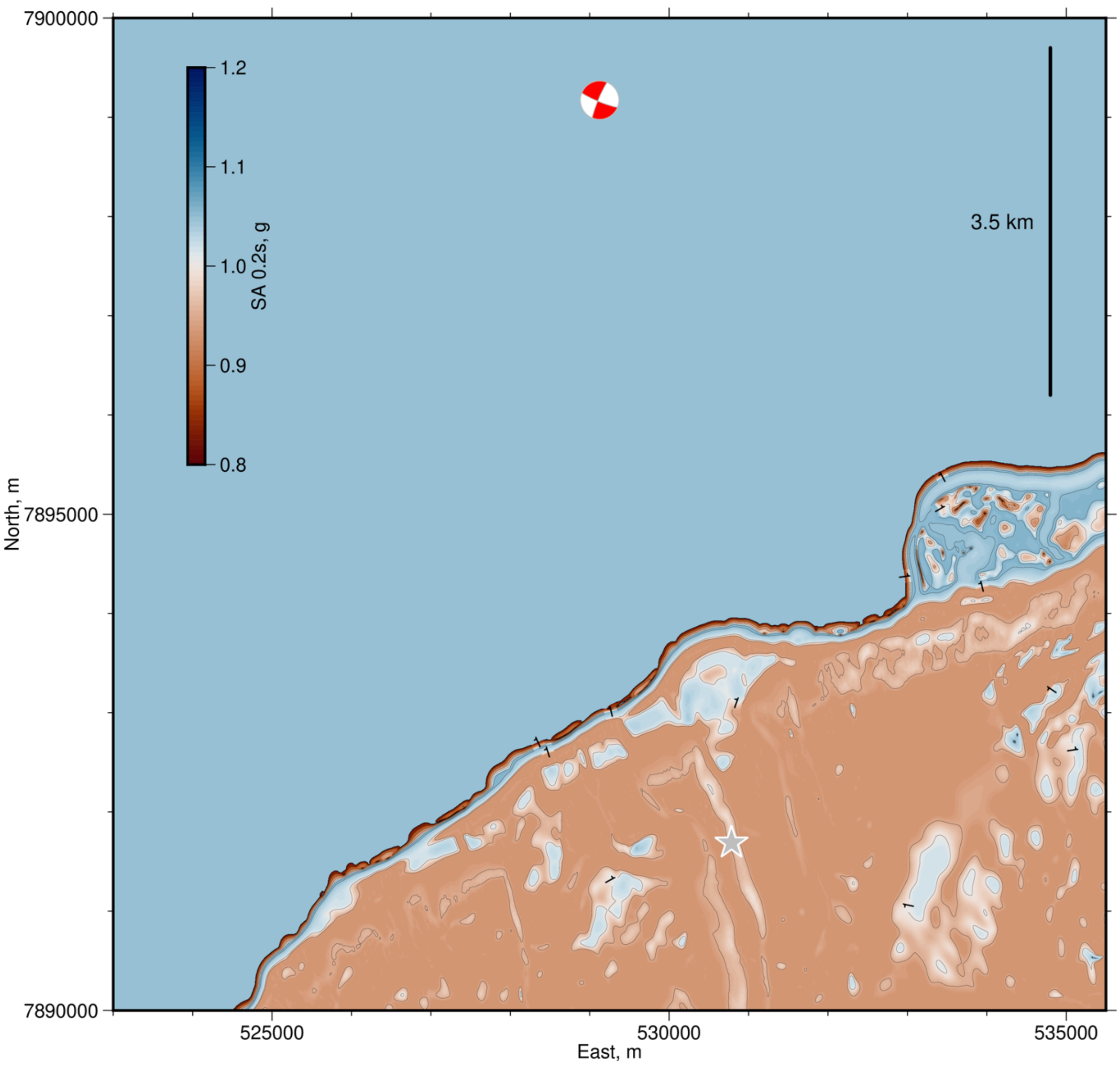

Figure S15 - Modeled 0.2-s spectral acceleration (SA0.2) for the 10 March 2025 Mw 6.5 Jan Mayen earthquake. Colors and contours represent modeled spectral acceleration (g). The focal mechanism (red beach ball) marks the mainshock epicenter, and the gray star indicates the Kjerulf Glacier rock failure source area. The modeled SA0.2 at the rock failure source is approximately 1.0 g.

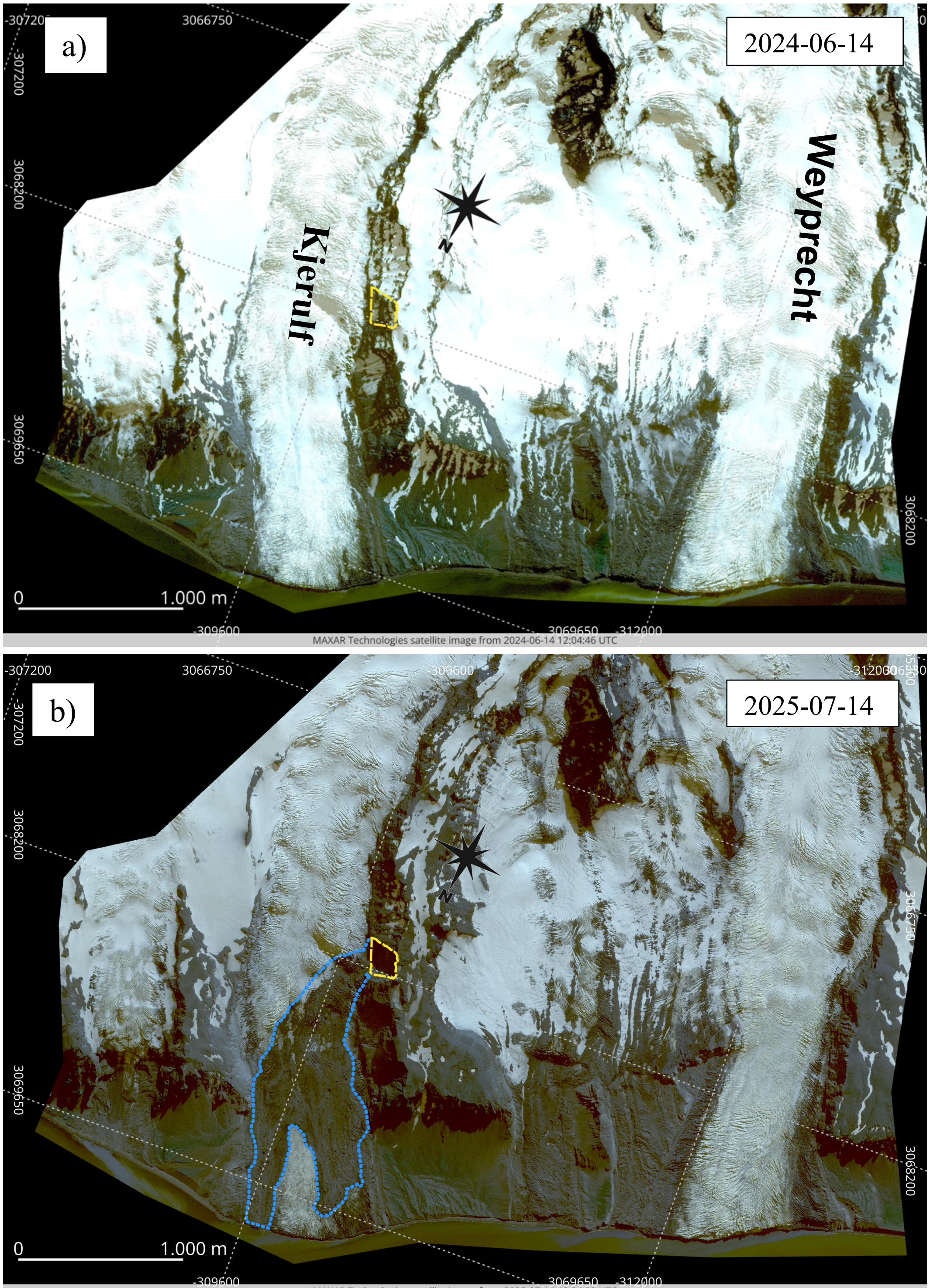

Figure S16 – High-resolution satellite view of the northwest most area of the Jan Mayen Island with the location of the Weyprecht and Kjerulf Glacier from MAXAR photochromatic image (Satellite image © 2025 Maxar Technologies Provided by European Space Imaging) taken on 14 June 2024 (a) and 14 July 2025 (b). The images document the major geomorphic changes following the 10 March 2025 Mw 6.5 earthquake, with the rock avalanche source area at Kjerulf Glacier outlined by the yellow dashed polygon in both a) and b), while the debris deposit post rock avalanche is outlined by the blue dashed polygon in b).

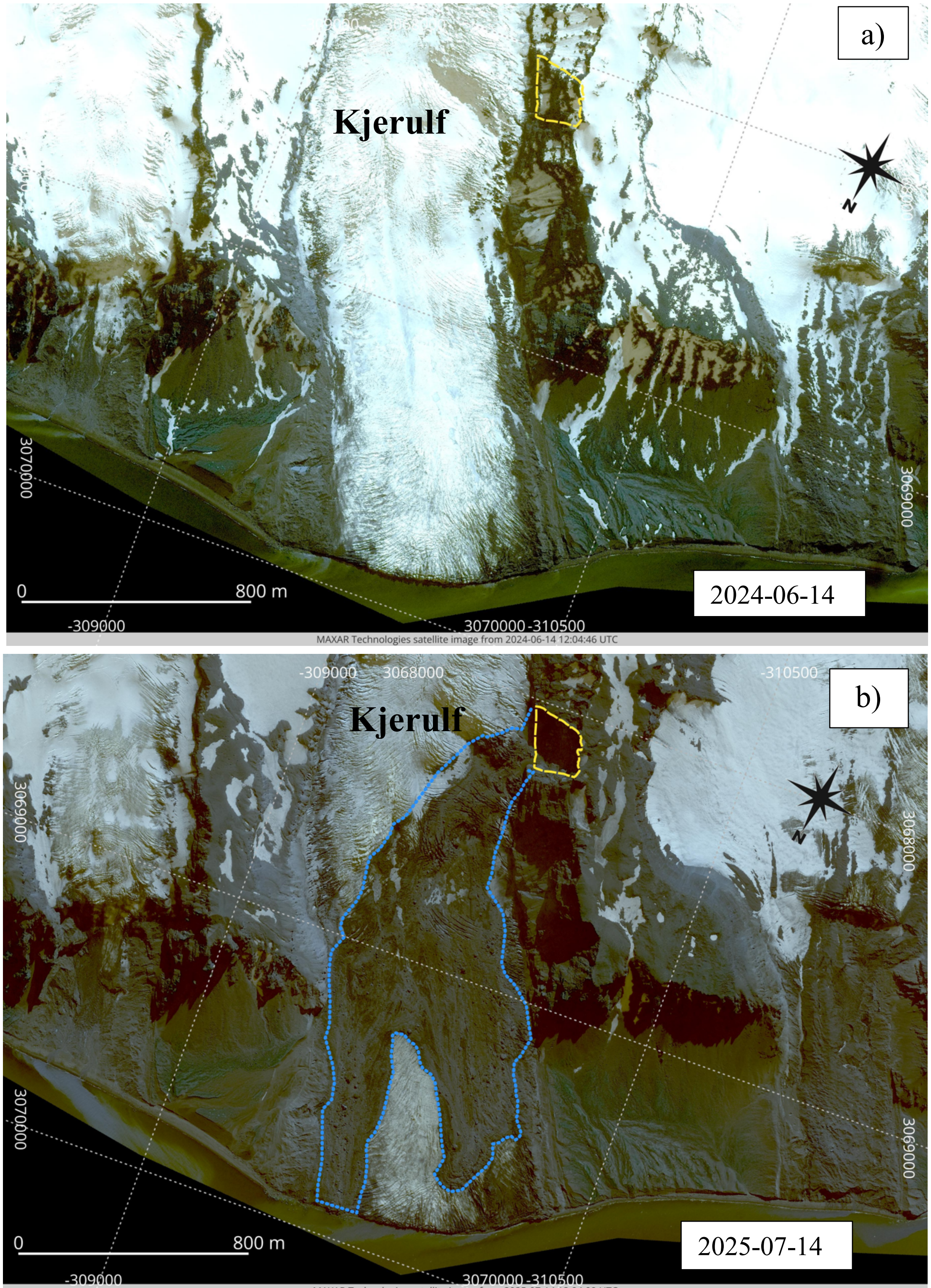

Figure S17 – a) and b) shows a pre- and post-earthquake view of the Kjerulf Glacier of MAXAR image taken on 14 June 2024 and 14 July 2025 (Satellite image © 2025 Maxar Technologies Provided by European Space Imaging). Rock avalanche source area at Kjerulf Glacier is outlined by the yellow dashed polygon in both a) and b), while the debris deposit post rock avalanche is outlined by the blue dashed polygon in b).

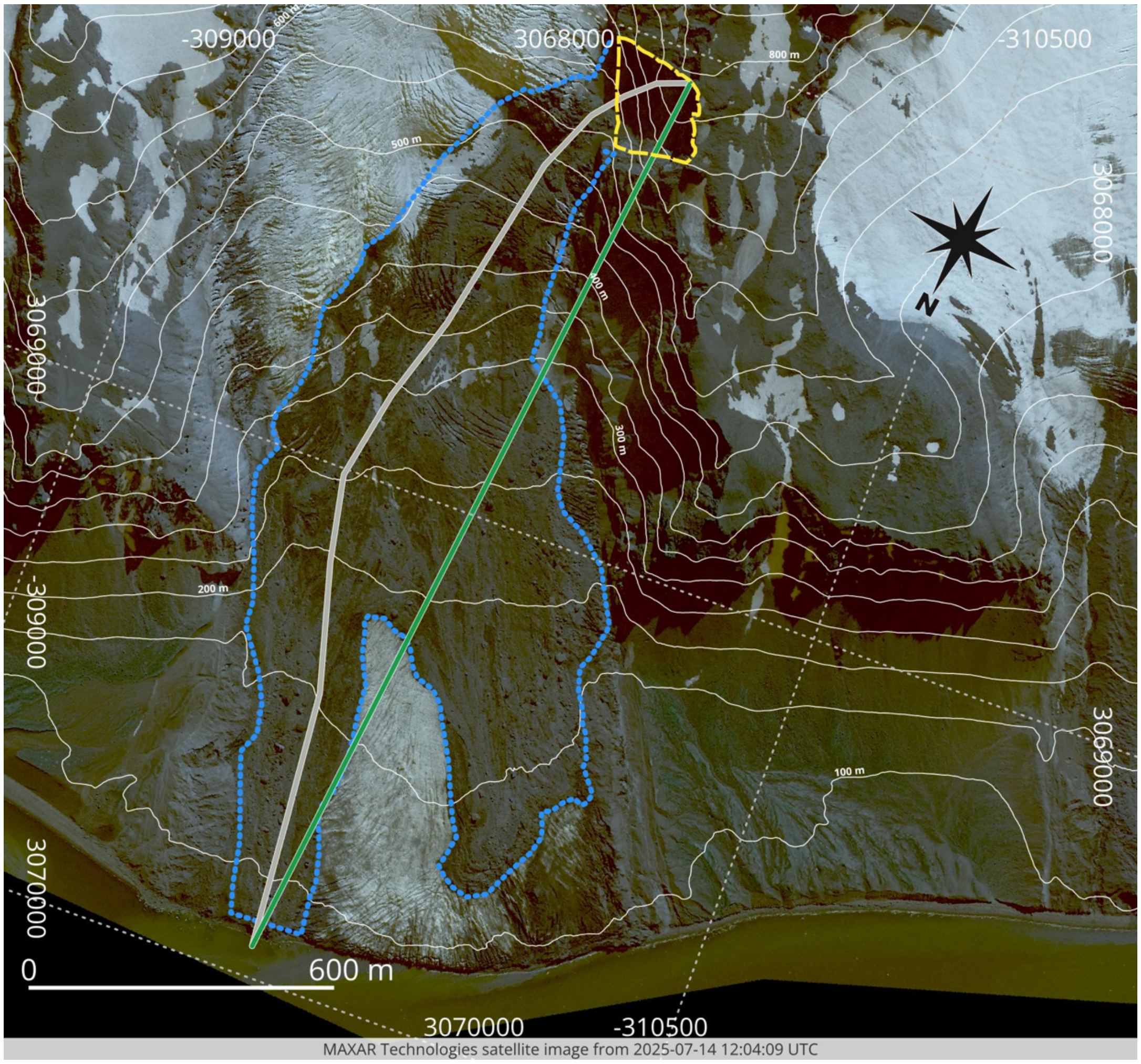

Figure S18 – Post-earthquake view of the Kjerulf Glacier of MAXAR image taken on 14 June 2024 and 14 July 2025 (Satellite image © 2025 Maxar Technologies Provided by European Space Imaging). Rock avalanche source area at Kjerulf Glacier is outlined by the yellow dashed polygon, while the debris deposit post rock avalanche is outlined by the blue dashed polygon. White contour lines shows the 2 m ArcticDEM topography mosaic.

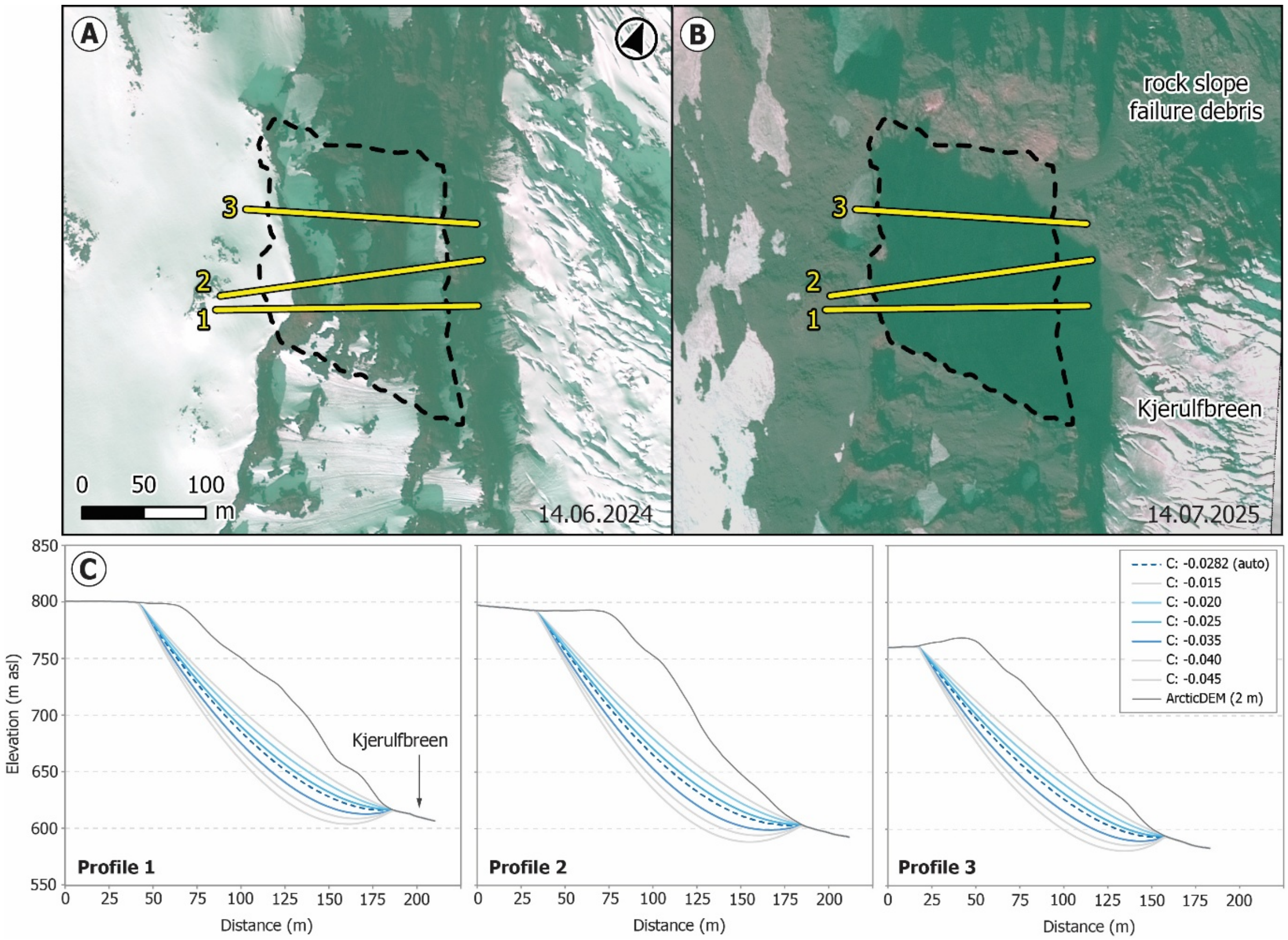

Figure S19 – Rock avalanche source volume estimation from pre- and post-earthquake MAXAR colour-infrared satellite imagery (Satellite image © 2025 Maxar Technologies Provided by European Space Imaging) using Sloping Local Base-Level (SLBL) method. Source area and elevation profiles are shown on color-infrared rendering of (A) pre-event (14.06.2024) and (B) post-event (14.07.2025) WorldView images. (C) Computed basal failure surfaces shown along three representative profiles, generated using different SLBL tolerance (*C*) values. *C* controls the depth and smoothness of the reconstructed basal surface by defining the allowable deviation from the surrounding topography, where increasingly negative values produce deeper and smoother basal profiles. Geologically plausible basal surfaces (shown blue) define the likely source area volume range.

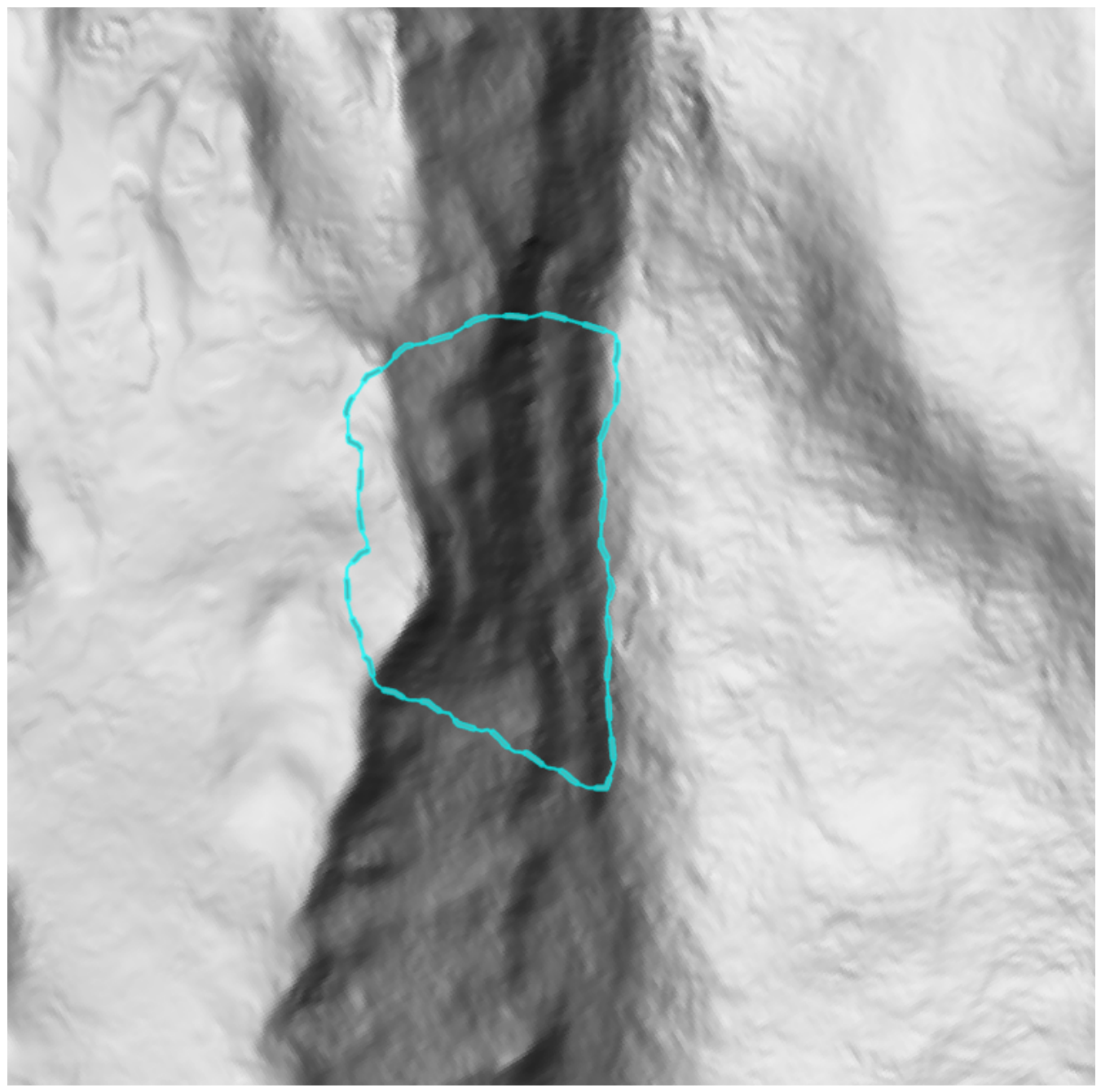

Figure S20 – Multidirectional hillshade mosaic from 2 m cell size ArcticDEM (https://arcticdem.apps.pgc.umn.edu/). The source area of the rock slope failure (blue polygon) was digitized through interpretation of co-registered MAXAR panchromatic imagery from 14 July 2025 (Satellite image © 2025 Maxar Technologies Provided by European Space Imaging) and used for volume calculation.

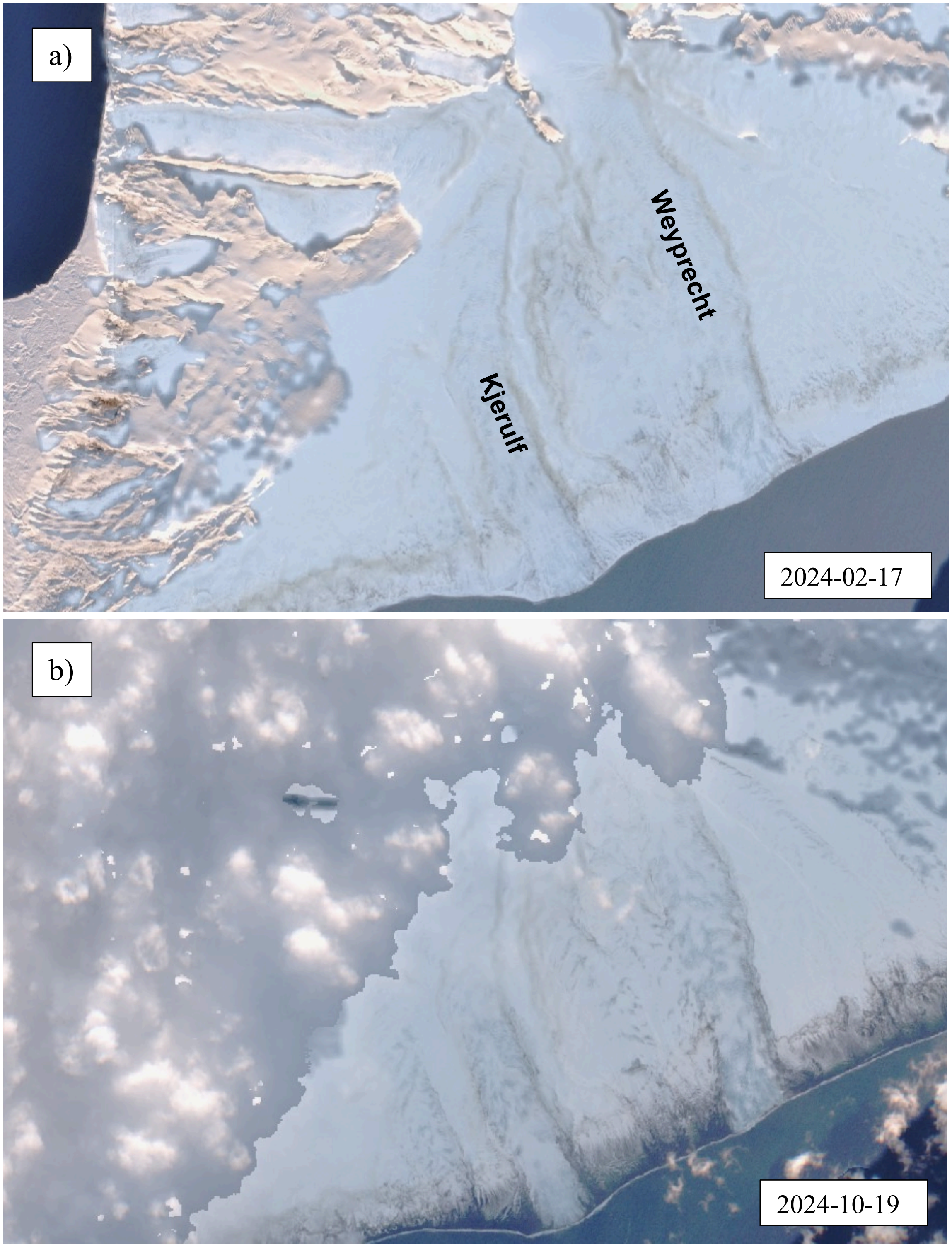

Figure S21 – Kjerulf and Weyprecht Glaciers pre-earthquake observations from Sentinel 2 satellite images. a) Picture taken on 17 February 2024 is shown in upper side, while picture taken on 19 October 2024 is shown on b).

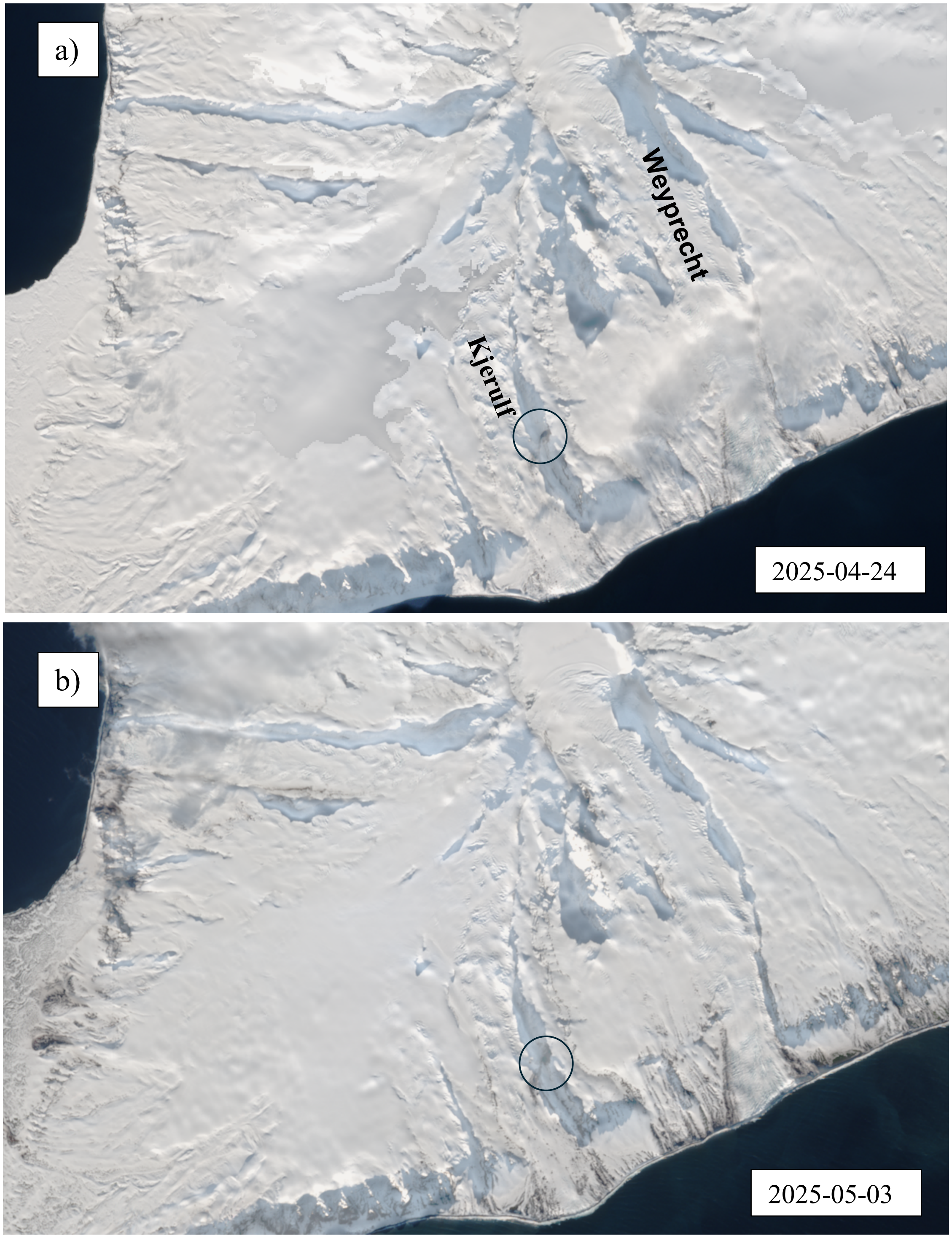

Figure S22 – Kjerulf and Weyprecht Glaciers post-earthquake observations from Sentinel 2 satellite images. a) Picture taken on 24 April, while picture taken on 03 May 2025 is shown on b). Black circus line indicates the area where rock slope failure occurred after the mainshock.

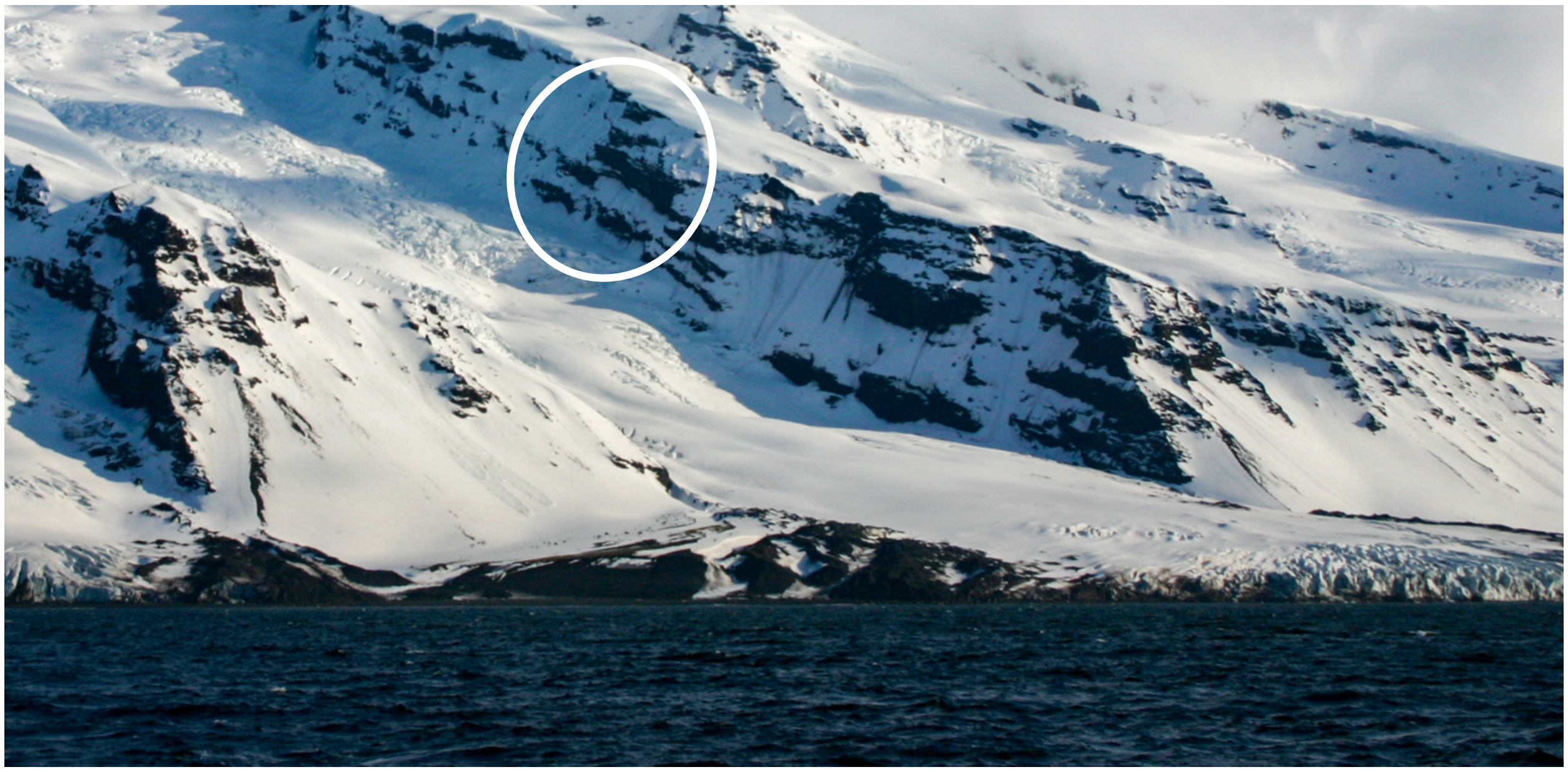

Figure S23 – Pre-earthquake oblique view of the Kjerulf Glacier front and adjacent rock slopes on Jan Mayen Island, showing the steep glacier-adjacent rock wall that later failed after the earthquake. Image taken on 8 June 2005 by Rolf Stange (used with permission).

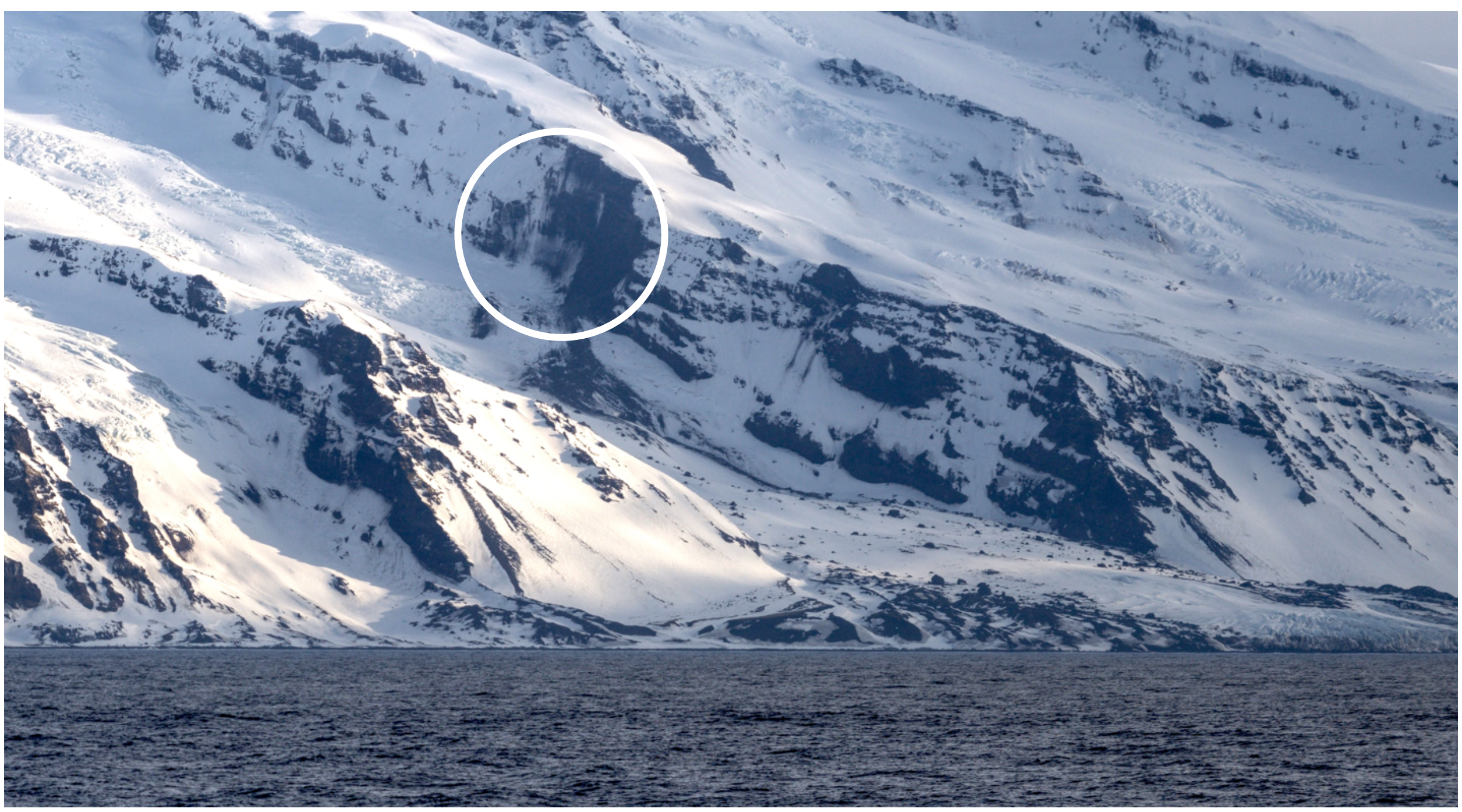

Figure S24 – Post-earthquake oblique view of the Kjerulf Glacier, showing the headscarp (dark area on adjacent rock wall), the downslope runout path, and debris deposition onto the glacier surface extending to the coastline distributed by the rock avalanche. Image taken on 5 May 2025 by Rong Wong (used with permission).

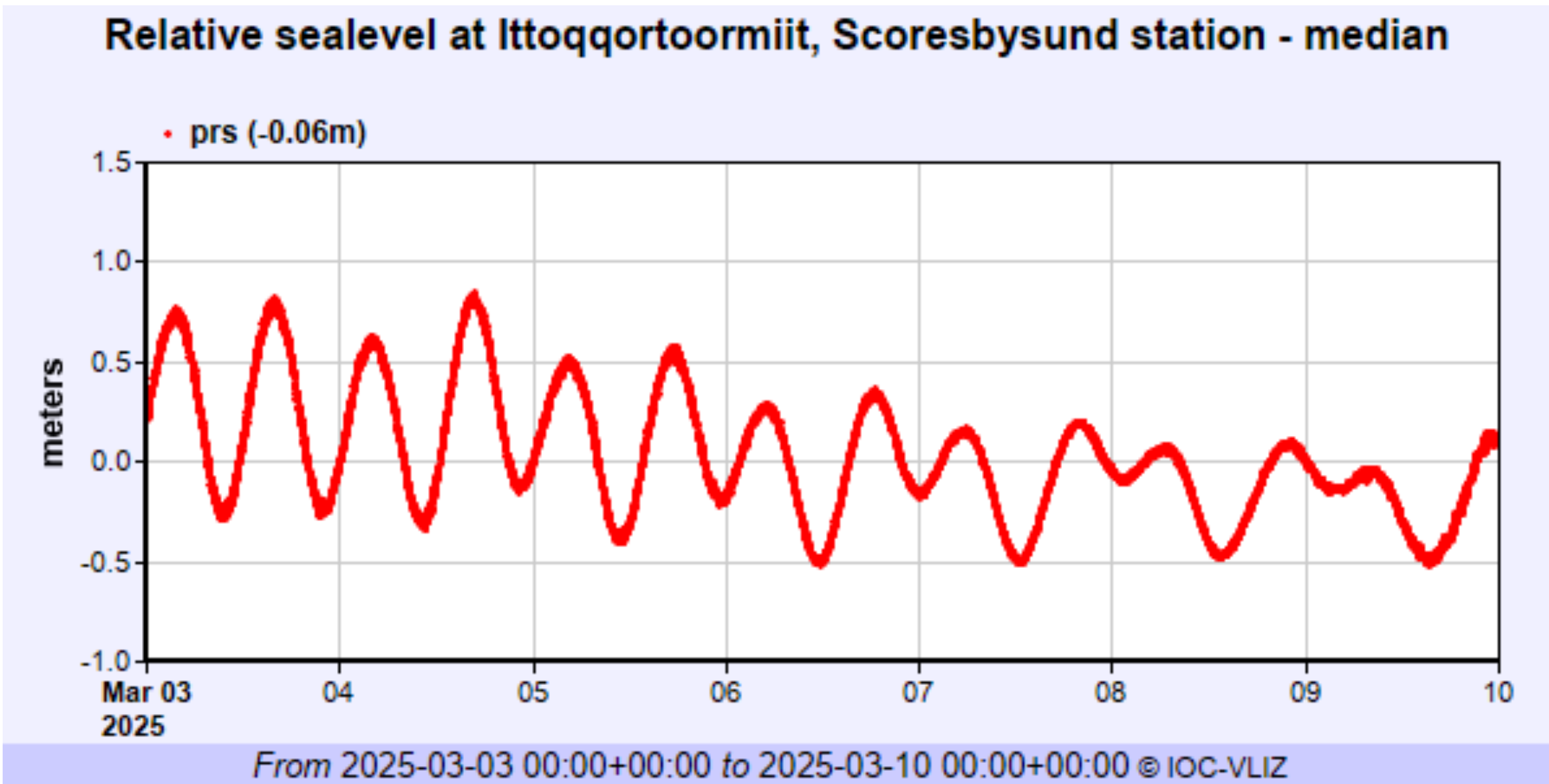

Figure S25 – Relative sea-level record at the Ittoqqortoormiit (Scoresbysund, East Greenland) tide-gauge station from 3 March to 10 March 2025. The time series indicates a regular tidal variability (~0.3-0.5 m amplitude) and shows no anomalous.

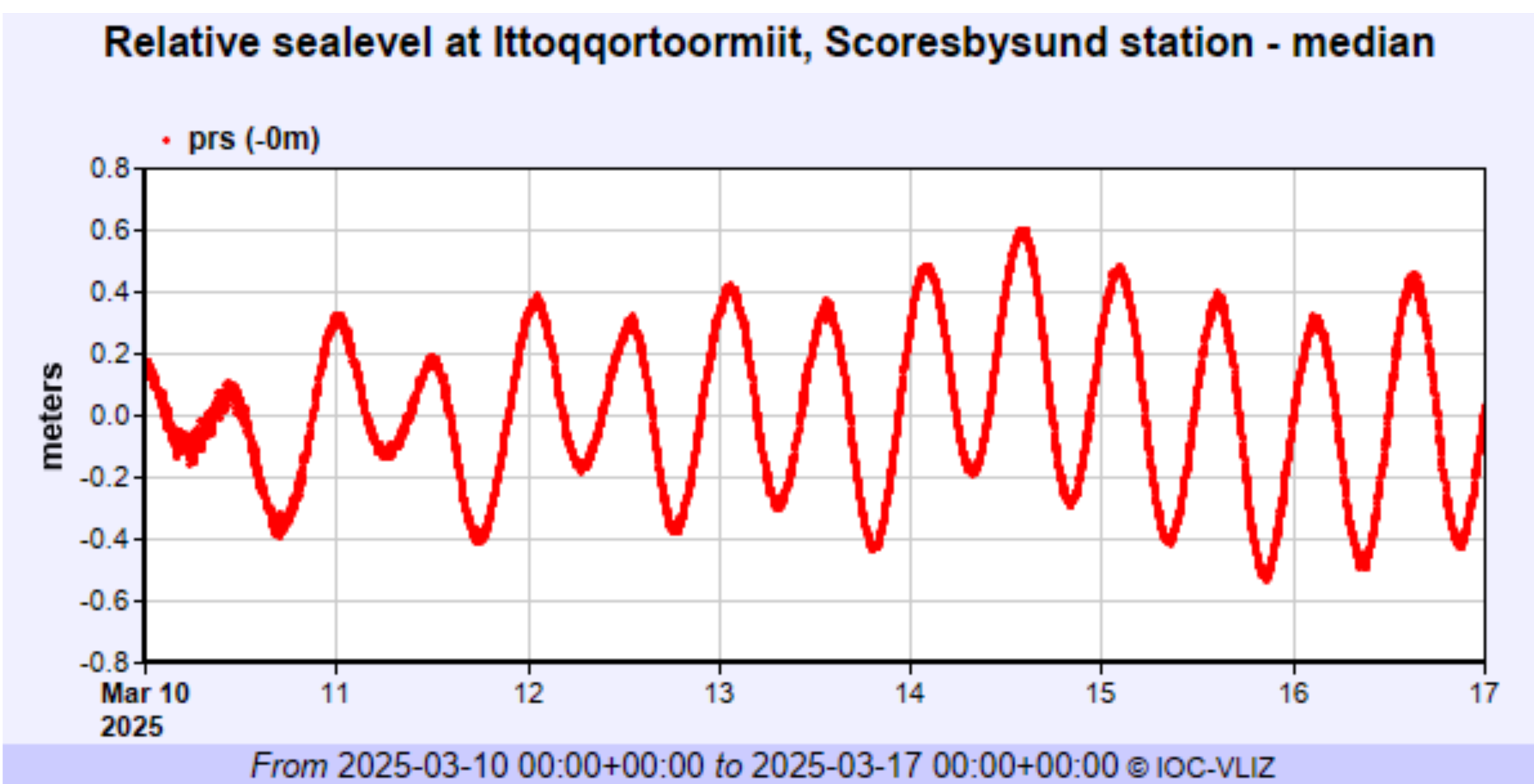

Figure S26 – Relative sea-level record at the Ittoqqortoormiit (Scoresbysund, East Greenland) tide-gauge station from 10 March to 17 March 2025. The time series indicates a regular tidal variability (~0.3-0.6 m amplitude) and shows no anomalous.

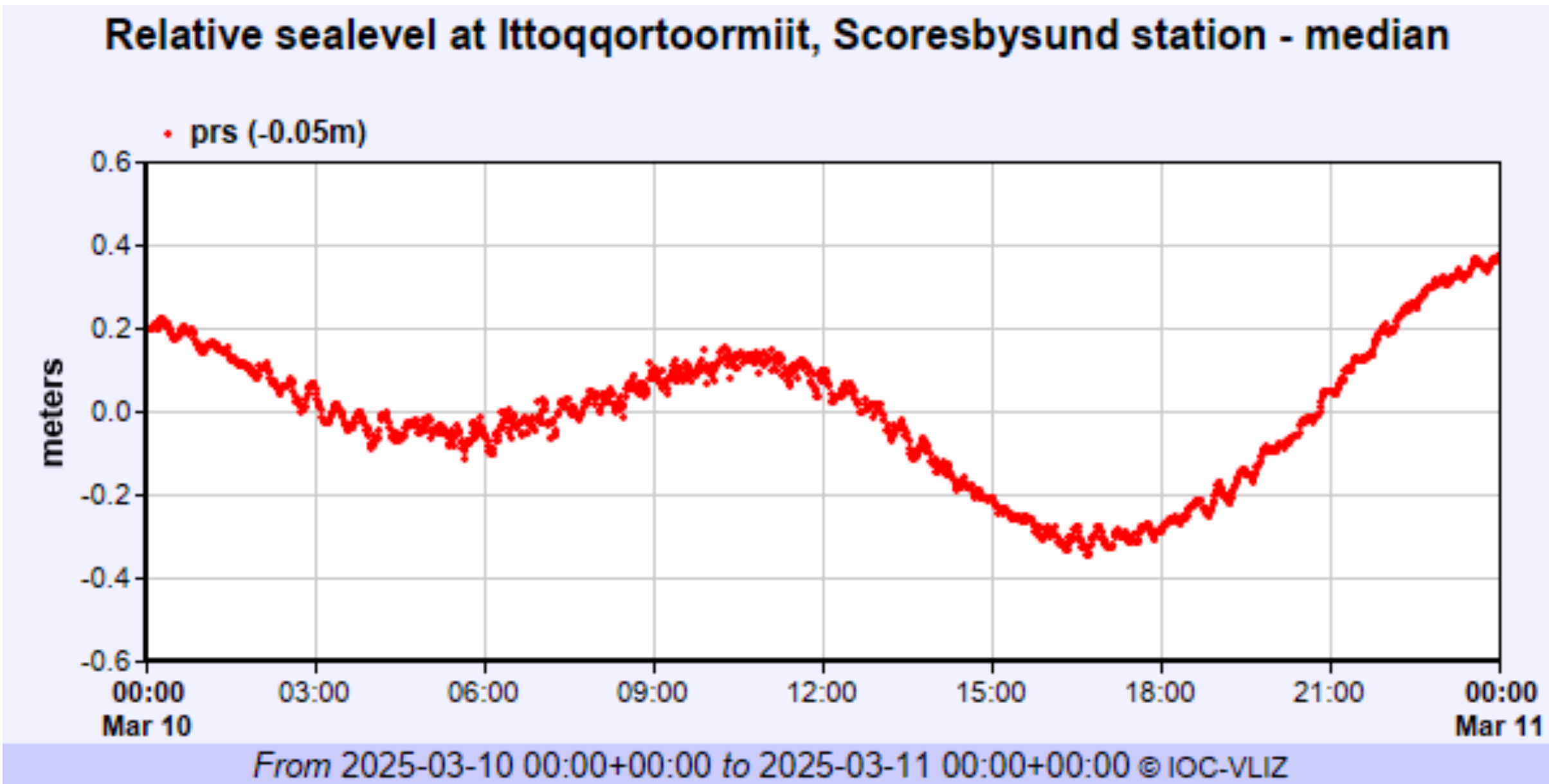

Figure S27 – Relative sea-level record at the Ittoqqortoormiit (Scoresbysund, East Greenland) tide-gauge station on 10 March 2025. The time series prese a smooth tidal variability with maximum variance of 0.10-0.15m, without any abrupt perturbations or high-frequency oscillations in the first 24 hours post-earthquake, indicating no detectable tsunami signal.

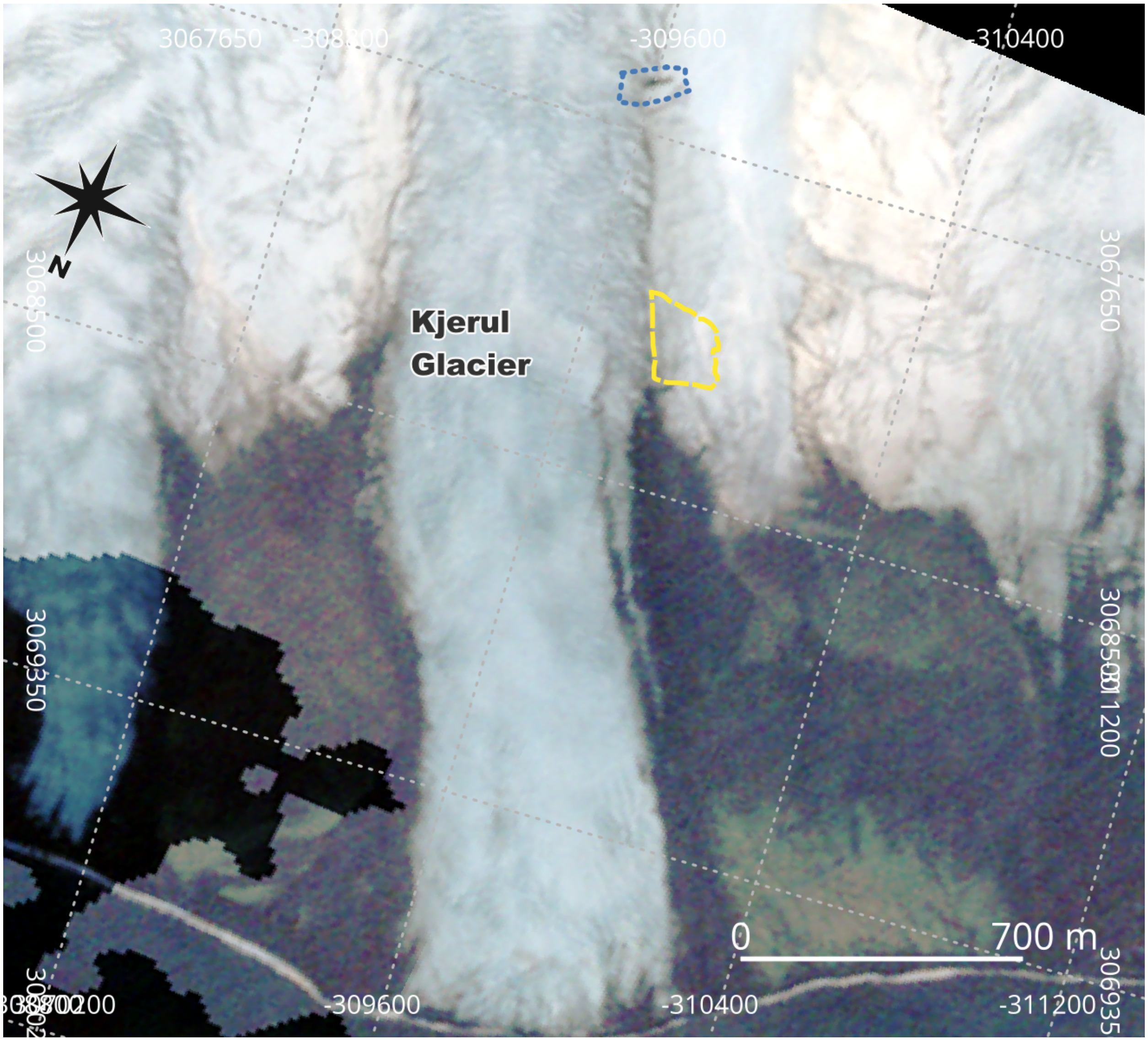

Figure S28 – Sentinel-2 imagery of 19 October 2017. Blue dashed represent the contour of the small rock failure occurred in October 2017, while the yellow dashed line shows the total area failure after the Mw 6.5 2025 earthquake.

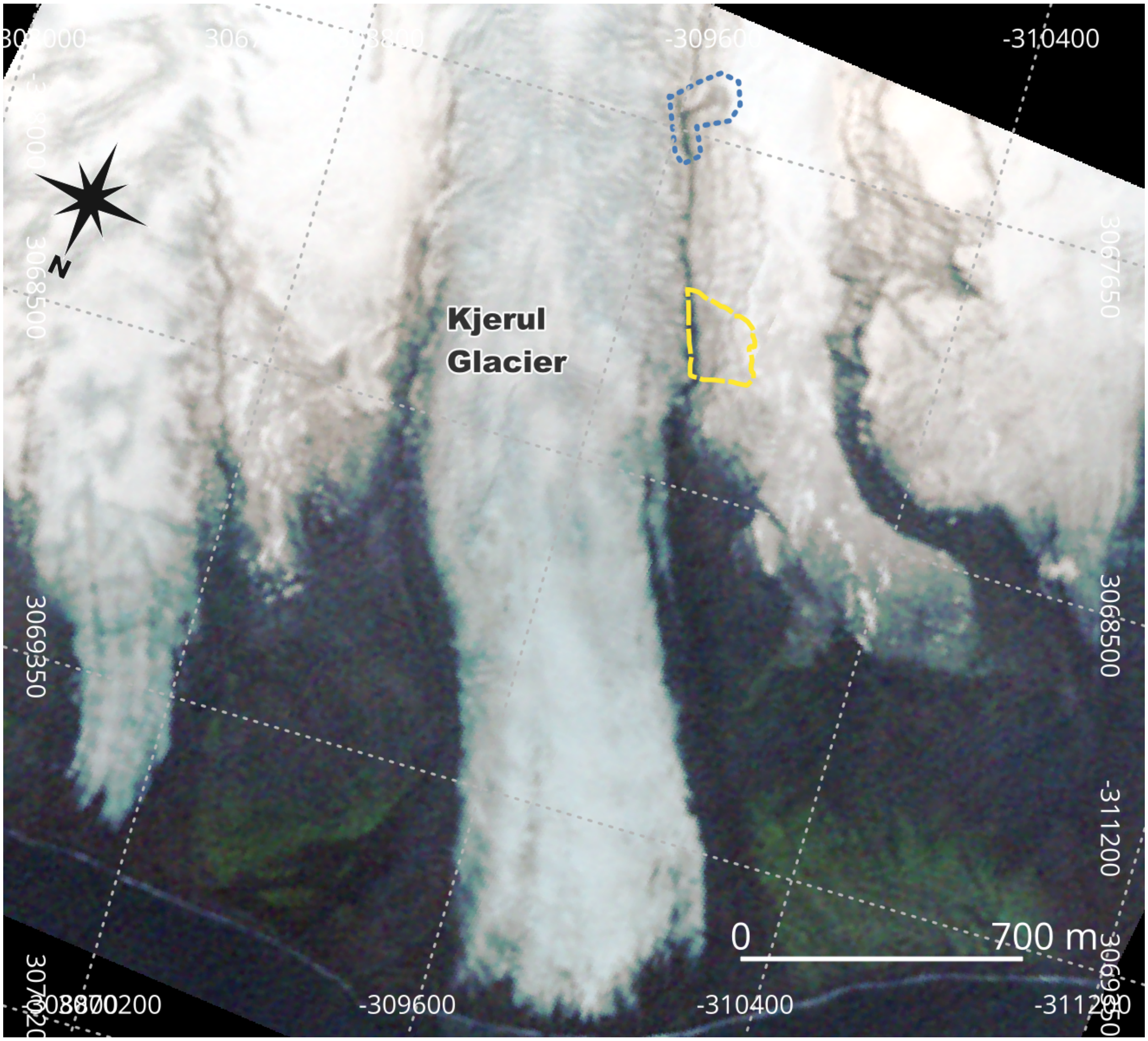

Figure S29 – Sentinel-2 imagery of 29 September 2019. Blue dashed represent the contour of the small rock failure occurred in the end of September 2019, while the yellow dashed line shows the total area failure after the Mw 6.5 2025 earthquake.

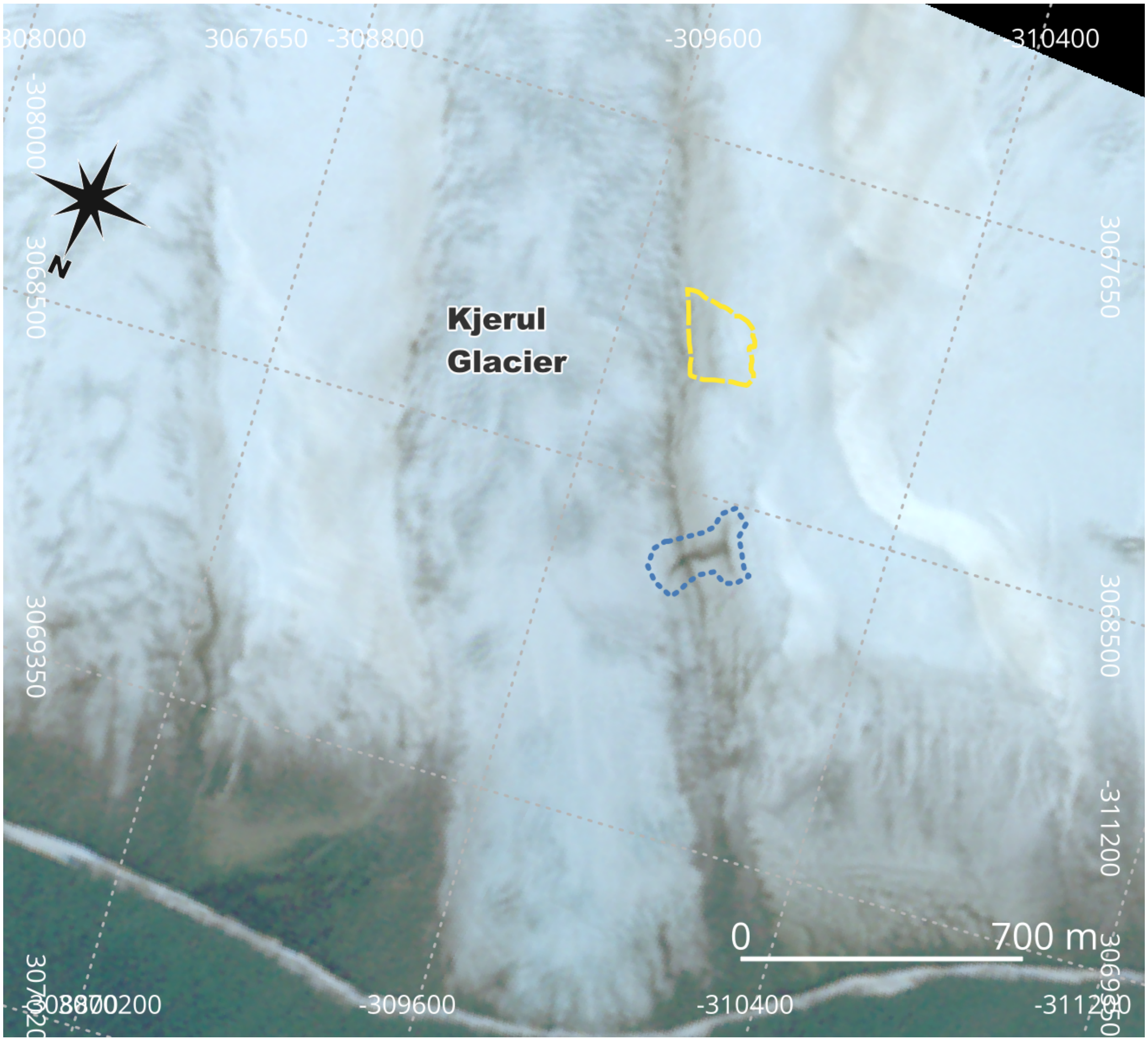

Figure S30 – Sentinel-2 imagery of 2 October 2023. Blue dashed represent the contour of the small rock failure occurred in October 2023, while the yellow dashed line shows the total area failure after the Mw 6.5 2025 earthquake.

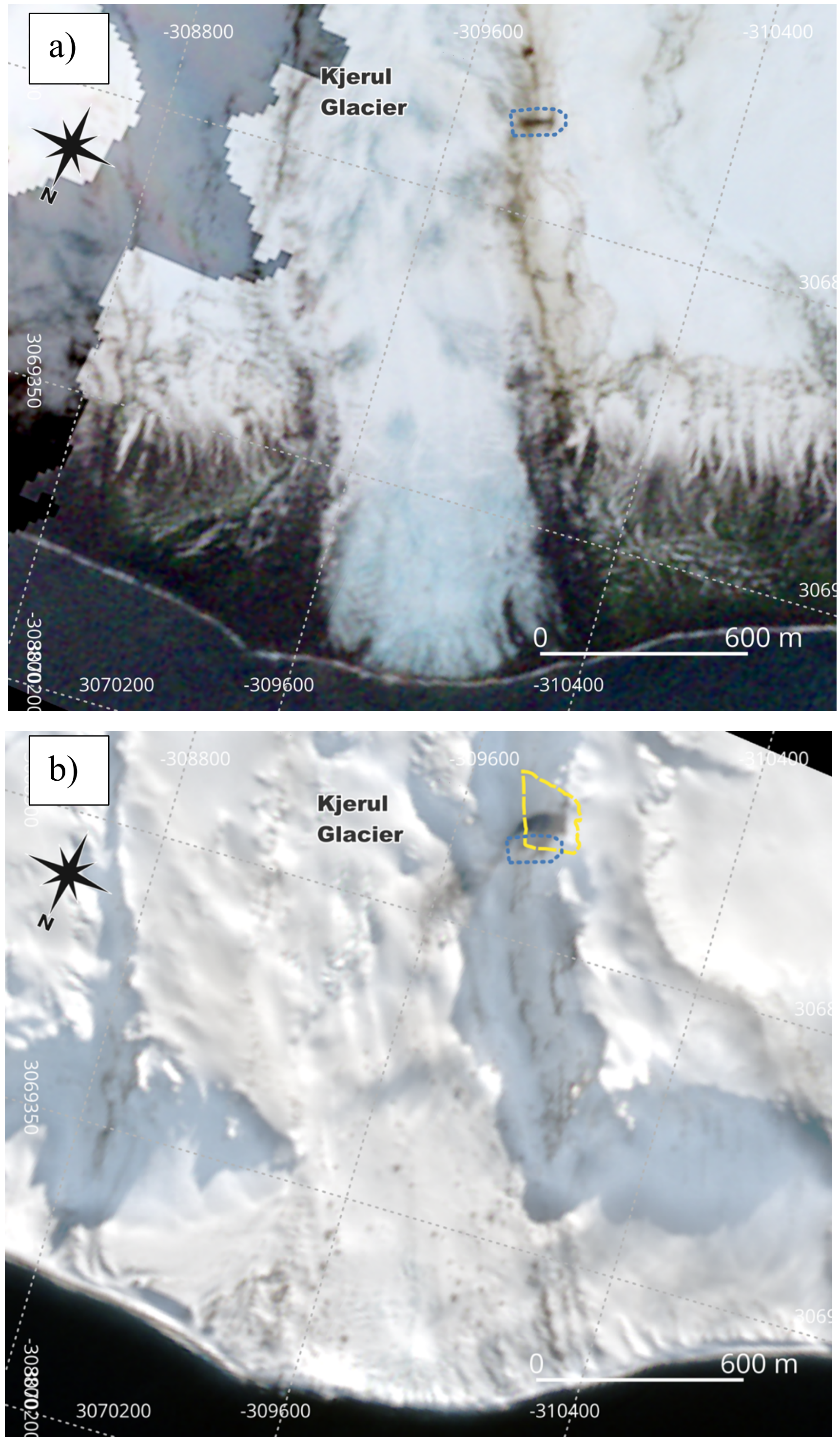

Figure S31 – a) Sentinel-2 imagery of 20 October 2024. Blue dashed represent the contour of the small rock failure occurred in October 2024. b) Post-earthquake view of 24 April 2025, with part of the large heard generated by the failure after the Mw 6.5 2025 earthquake visible in area indicated by the yellow dashed line. The whole area of the 2025 heard scarp was completely visible only after the snow melting, such as the July image presented in Figure S19b and Figure S20b.

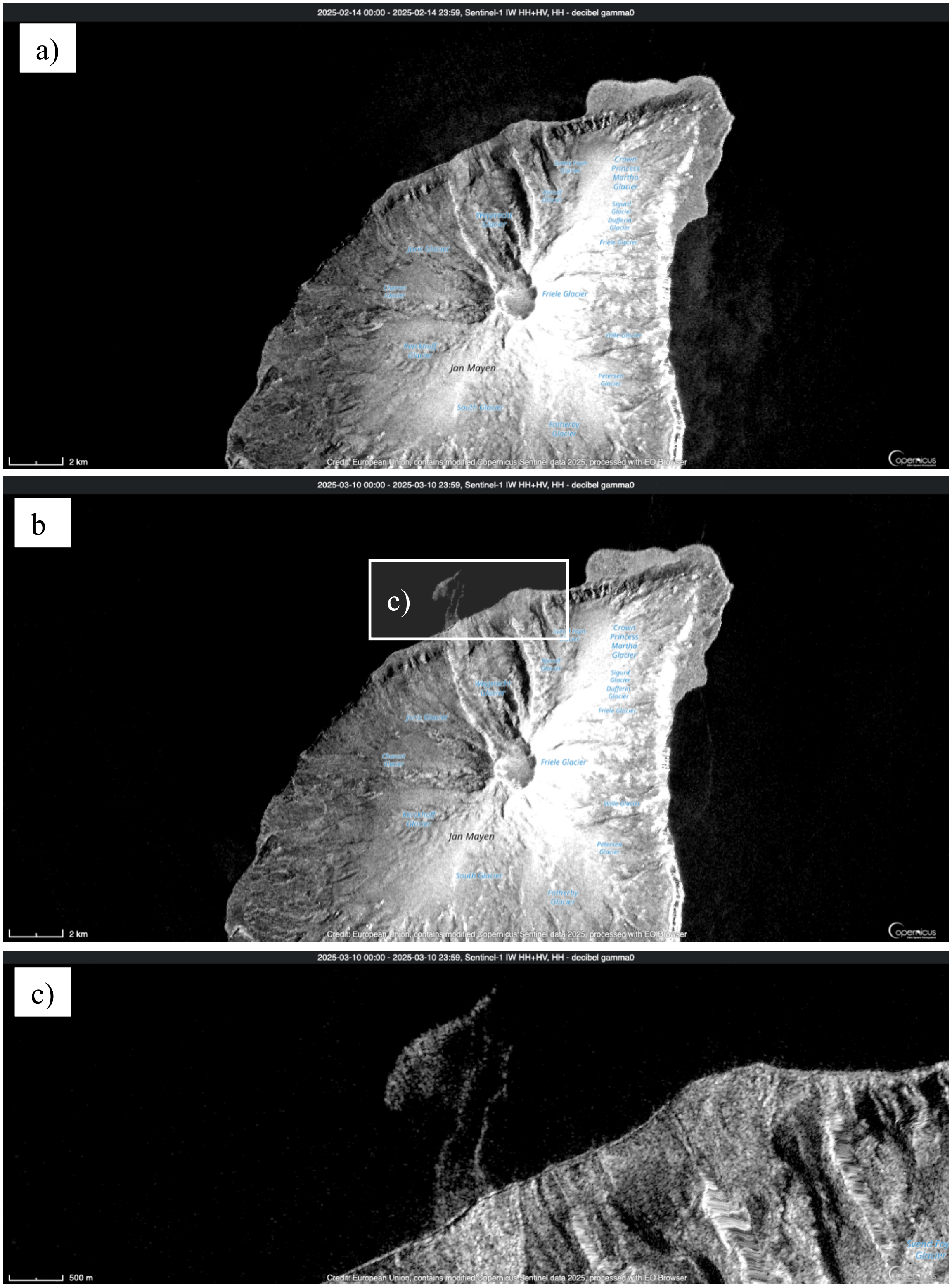

Figure S32 – Sentinel-1 SAR satellite interferometric frames pre- and post-earthquake. a) Orthorectified gamma0 image from 16 days (2025/02/14) before the Mw 6.5 earthquake. b) The same as a) taken at four and a half hours after the earthquake (2025/03/10). c) Zoom-in with high reflectance in ice deposit over sea water in front of the Weyprecht Glacier.

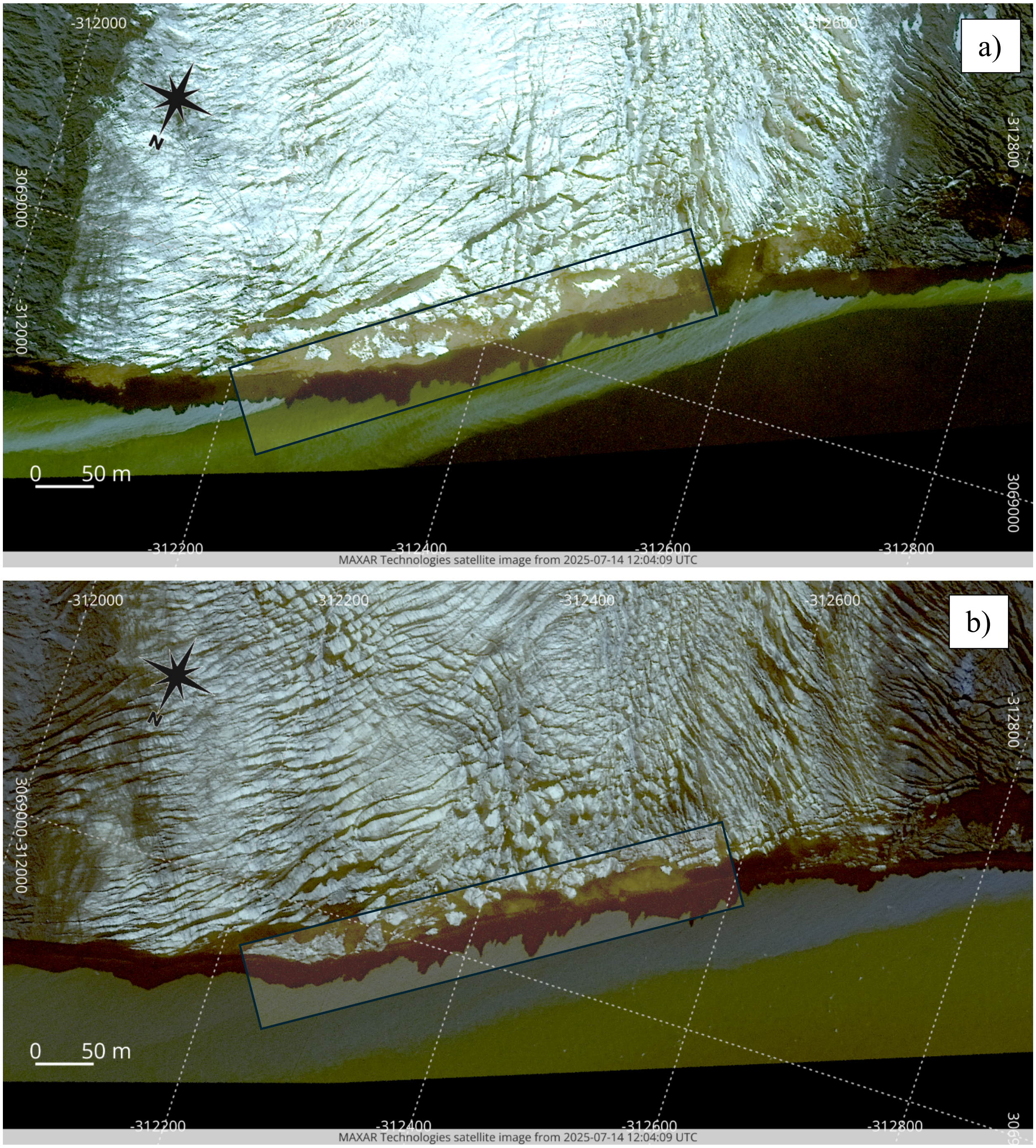

Figure S33 – Observation of the glacier calving occurred at Weyprecht Glacier comparing pre- and post-earthquake MAXAR photochromatic image of 14 June 2024 (a) and 14 July 2025 (b) (Satellite image © 2025 Maxar Technologies Provided by European Space Imaging).

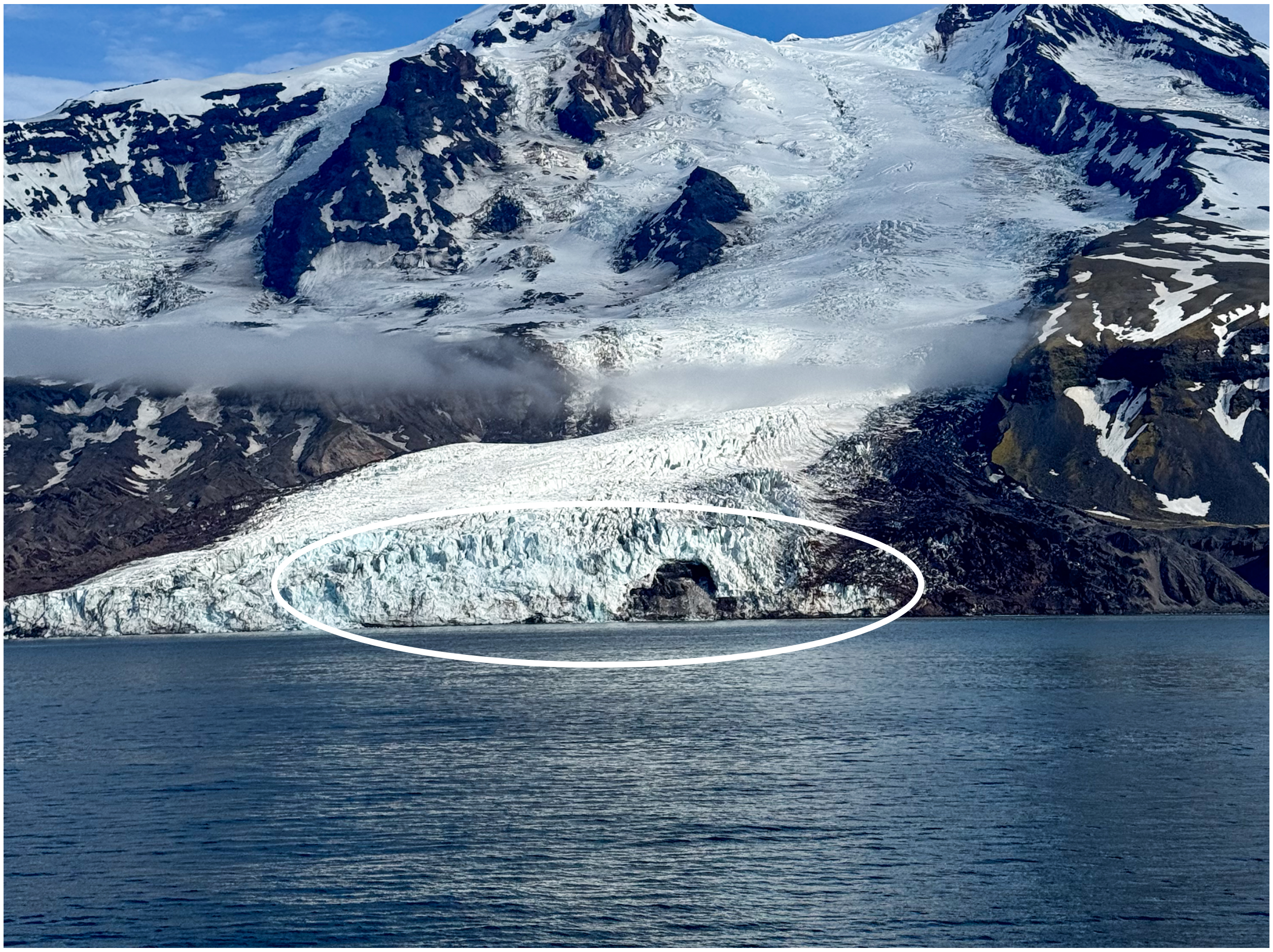

Figure S34 – Pre-earthquake observation of the front area of the Weyprecht Glacier. White line indicates the ice area deposited over sea water by the glacier calving after the earthquake. Image taken on 27 June 2024 by Robert Michael Poole (used with permission).

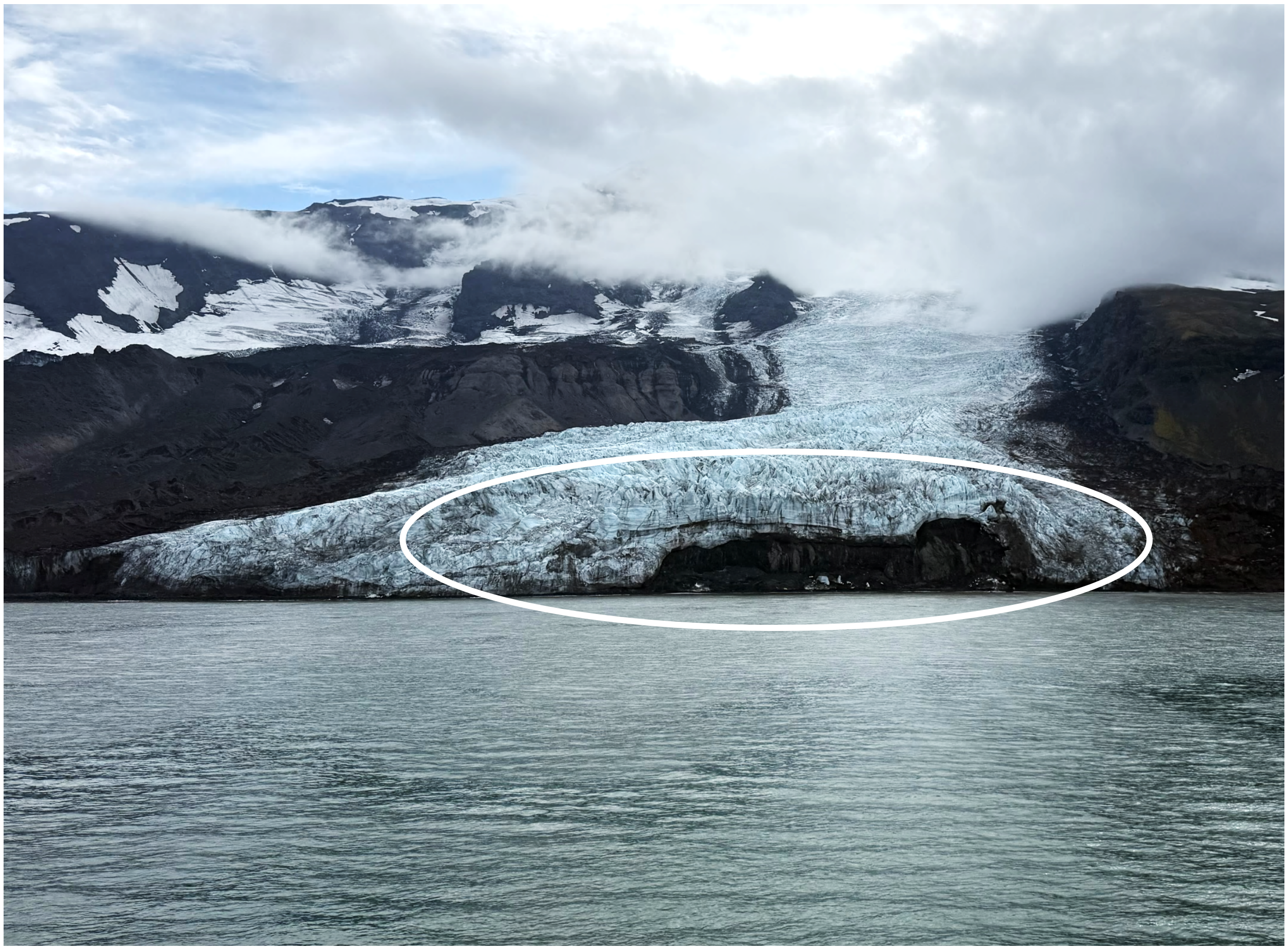

Figure S35 – Post-earthquake observation of the front area of the Weyprecht Glacier. White line indicate the ice area deposited over sea water by the glacier calving. Image taken on 2 August 2025 by Carla Marie Manly (used with permission).

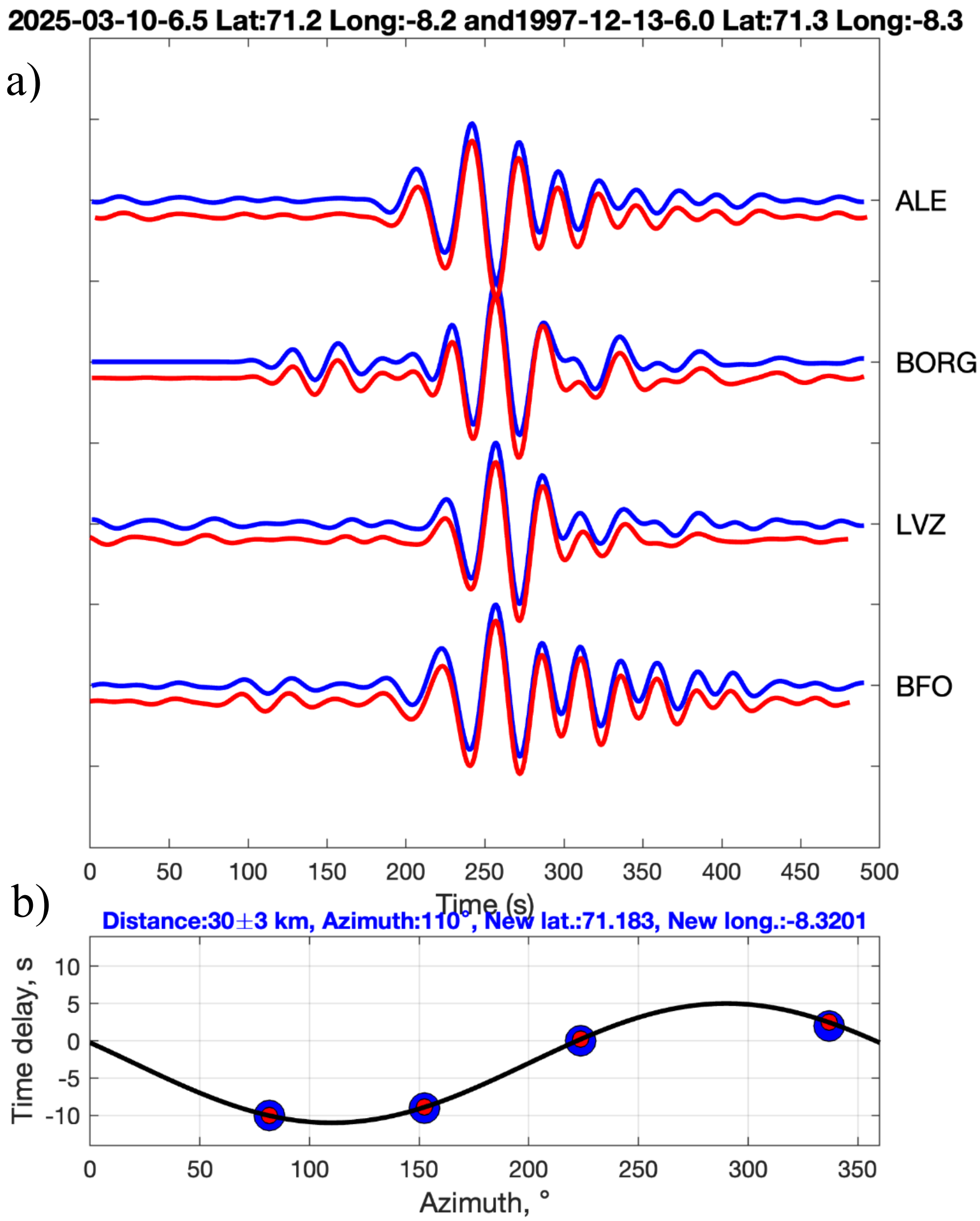

Figure S36 - Observed (blue) and synthetic (red) long-period Rayleigh-wave vertical-component seismograms recorded at ALE, BORG, LVX, and BFO stations in response to the 1997 Mw 5.6 relative relocation is shown in a). b) shows travel-time residuals (in seconds) as a function of azimuth (degrees), with the best-fitting travel-time curve shown.

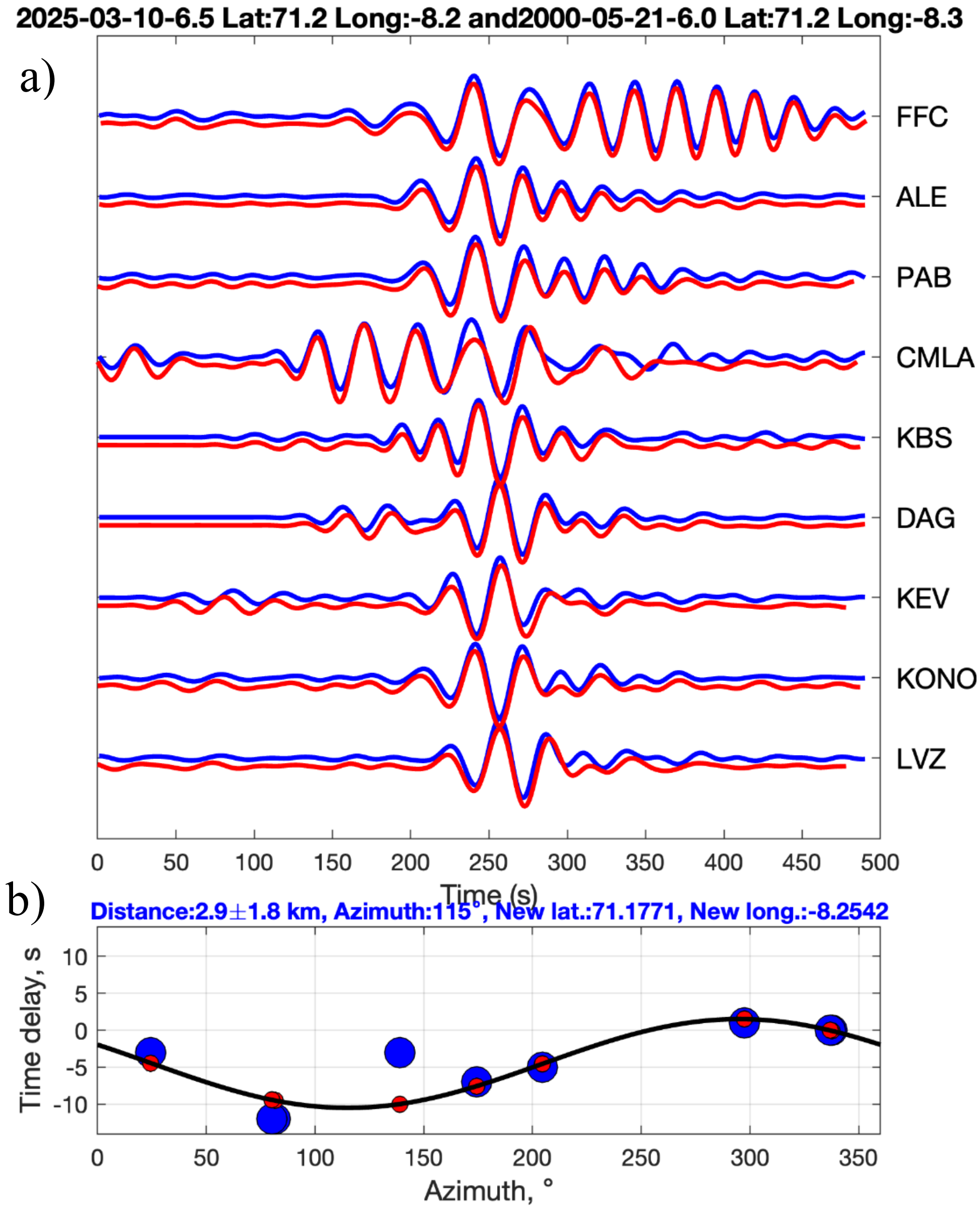

Figure S37 - Observed (blue) and synthetic (red) long-period Rayleigh-wave vertical-component seismograms recorded at FFC, ALE, OAB, CMLA, KBS, DAG, VEK, KONO, and LVZ stations in response to the 2000 Mw 6.0 relative relocation is shown in a). b) shows travel-time residuals (in seconds) as a function of azimuth (degrees), with the best-fitting travel-time curve shown.

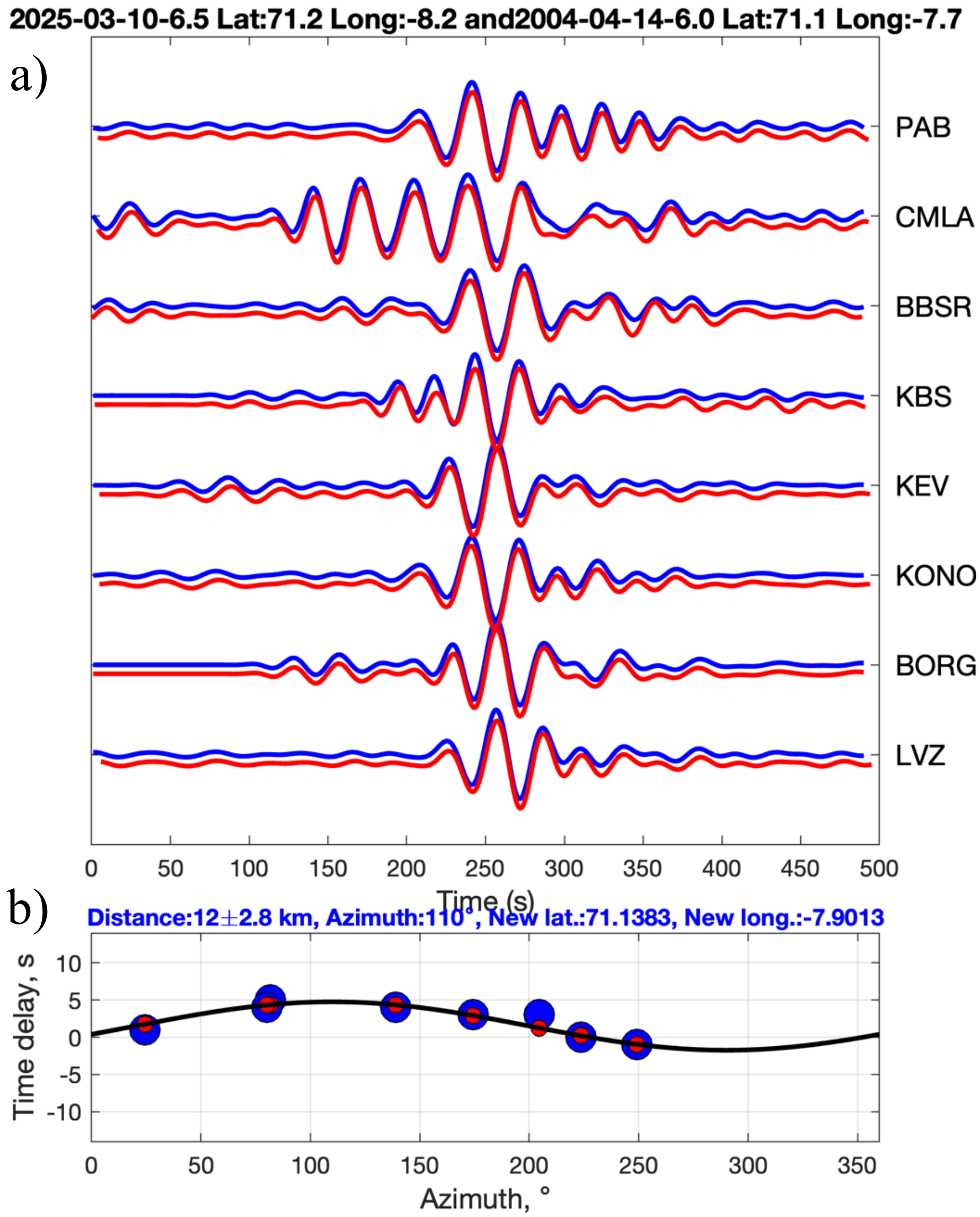

Figure S38 - Observed (blue) and synthetic (red) long-period Rayleigh-wave vertical-component seismograms recorded at PAB, CMLA, BBSR, KBS, KEV, KONO, BORG, and LVZ stations in response to the 2004 Mw 5.9 relative relocation is shown in a). b) shows travel-time residuals (in seconds) as a function of azimuth (degrees), with the best-fitting travel-time curve shown.

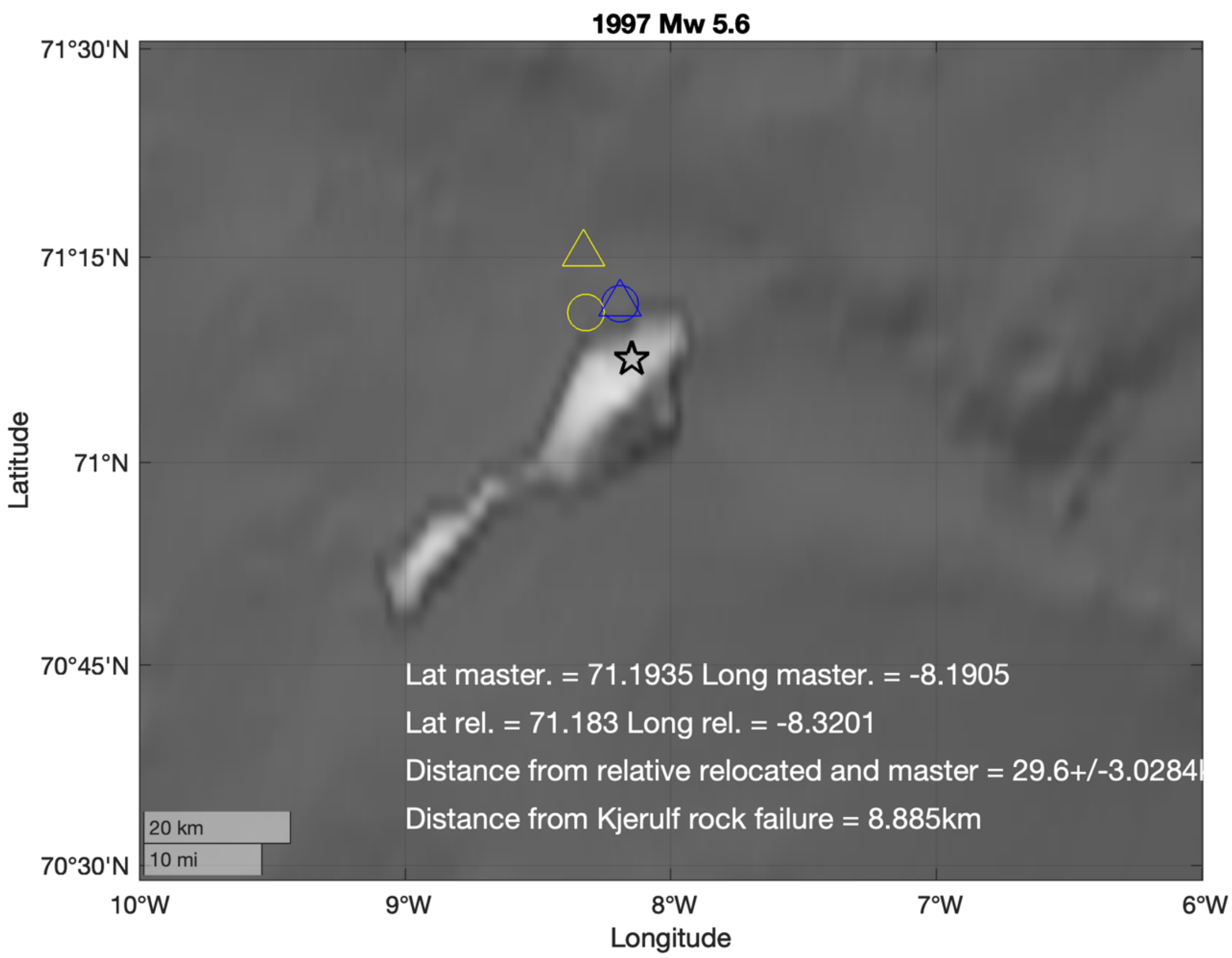

Figure S39 – Epicenter relocation of the 1997 Mw 5.6 earthquake. Blue triangle/circle represent the epicenter of the 2025 Mw 6.5, while the yellow triangle shows the USGS epicenter of the relocated event. Yellow circle indicates the relocated epicenter, while the black start indicates the rock failure area observed in MAXAR satellite images.

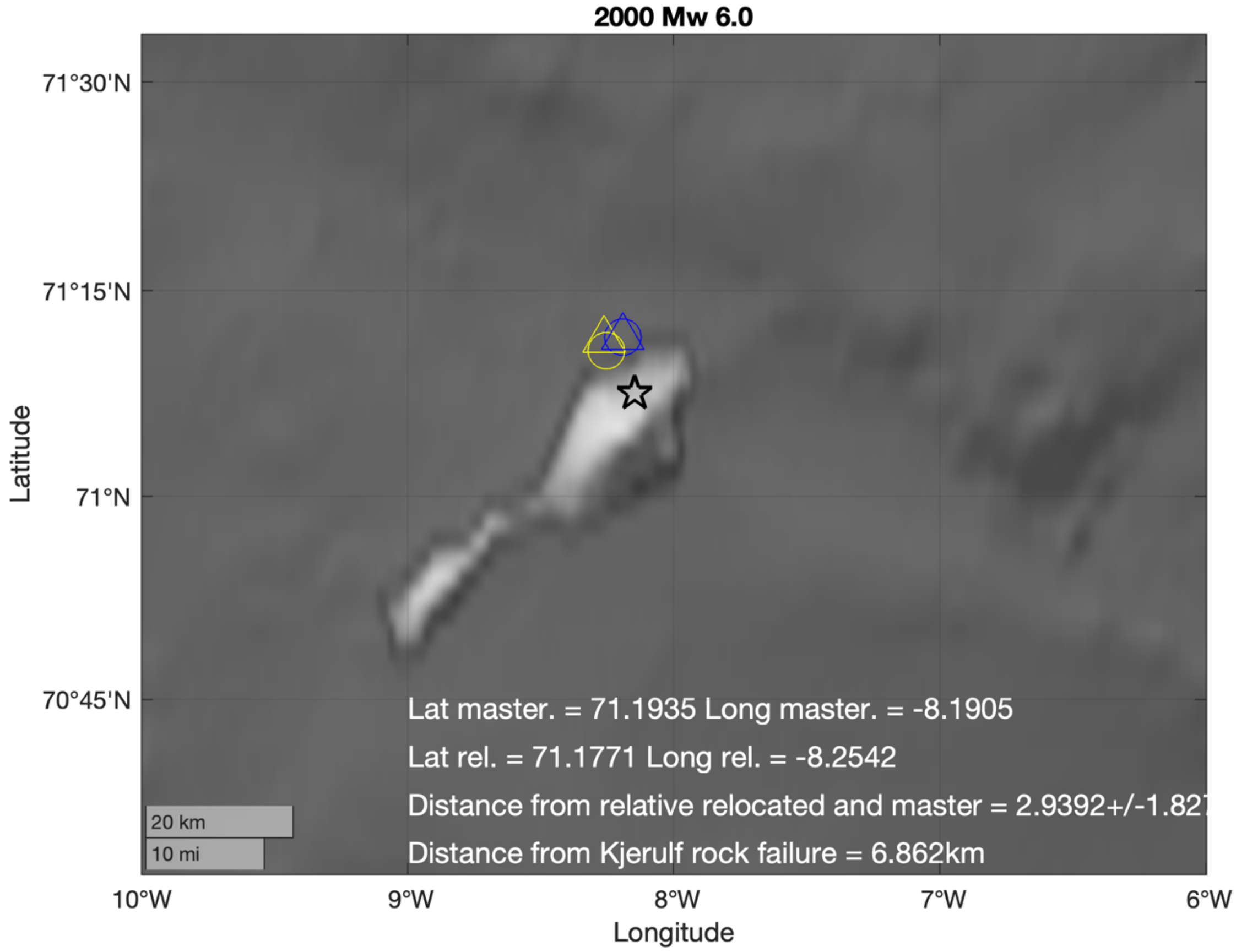

Figure S40 – Epicenter relocation of the 2000 Mw 6.0 earthquake. Blue triangle/circle represent the epicenter of the 2025 Mw 6.5, while the yellow triangle shows the USGS epicenter of the relocated event. Yellow circle indicates the relocated epicenter, while the black start indicates the rock failure area observed in MAXAR satellite images.

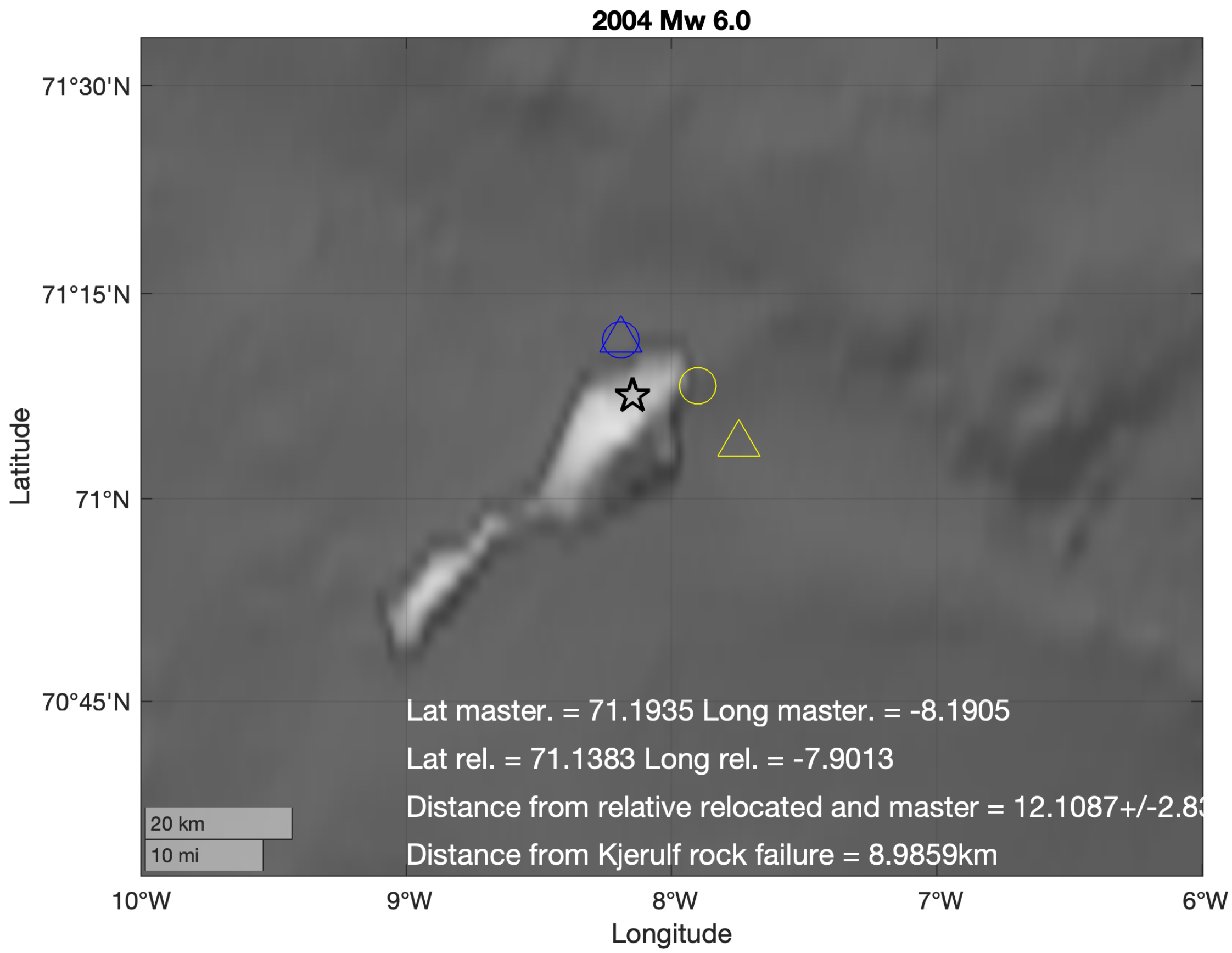

Figure S41 – Epicenter relocation of the 2004 Mw 5.9 earthquake. Blue triangle/circle represent the epicenter of the 2025 Mw 6.5, while the yellow triangle shows the USGS epicenter of the relocated event. Yellow circle indicates the relocated epicenter, while the black start indicates the rock failure area observed in MAXAR satellite images.

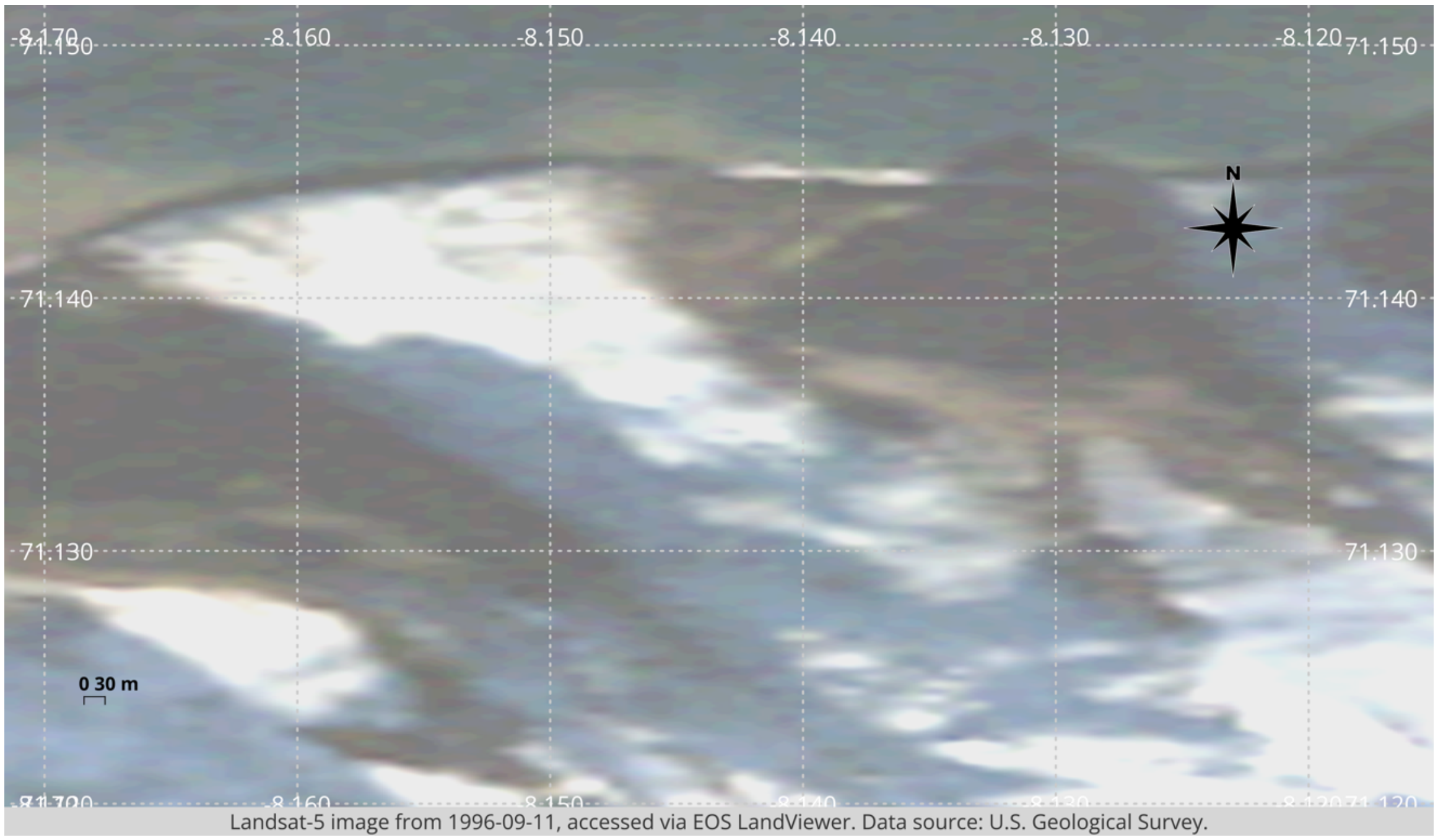

Figure S42 – Pre- 1997 Mw 5.6 earthquake Landsat=5 satellite image of Kjerulf Glacier from 1996-09-11. Image courtesy of U.S. Geological Survey, accessed via EOSDA LandViewer (https://eos.com/landviewer).

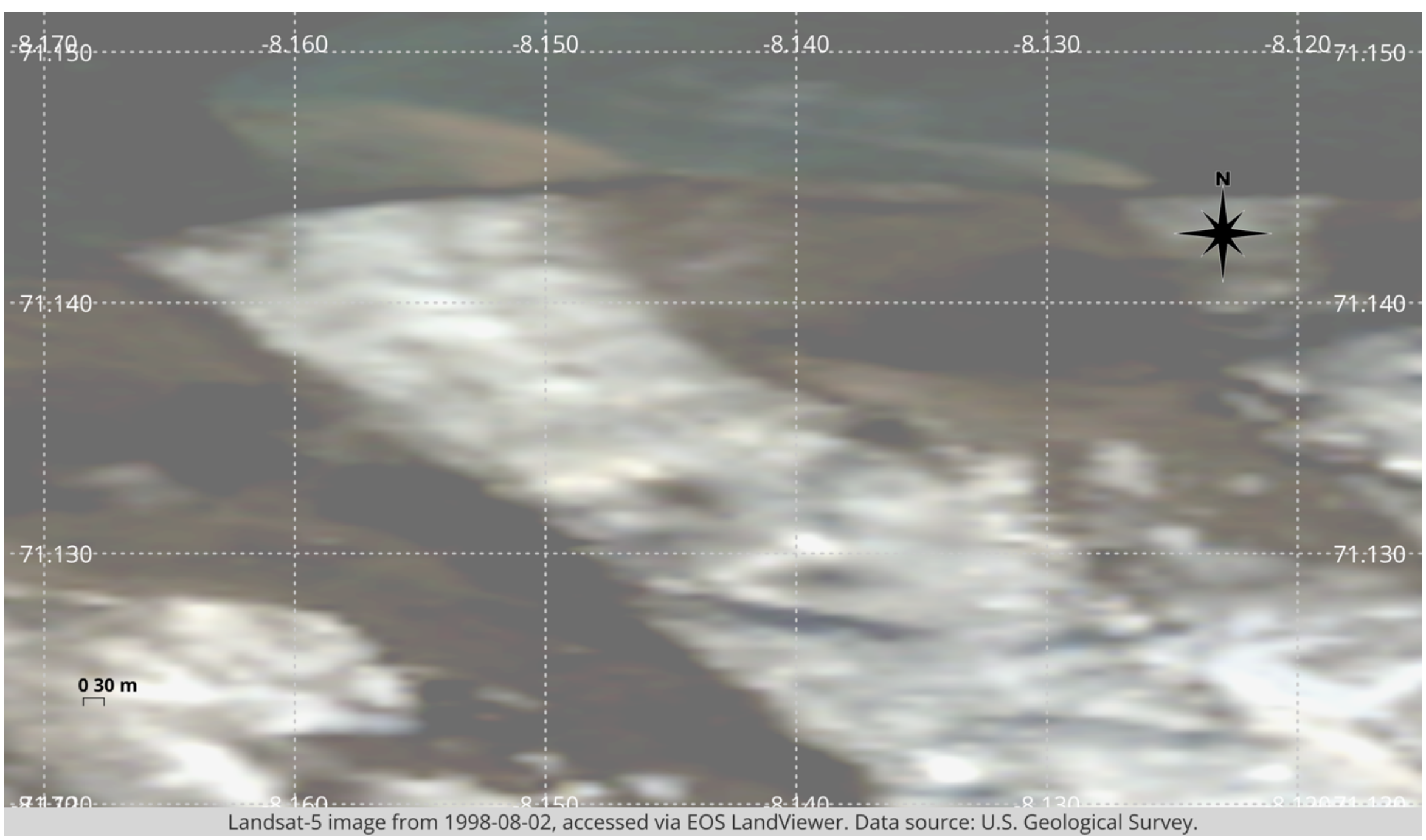

Figure S43 – Post- 1997 Mw 5.6 earthquake Landsat-5 satellite image of Kjerulf Glacier from 1998-08-02. Image courtesy of U.S. Geological Survey, accessed via EOSDA LandViewer (https://eos.com/landviewer).

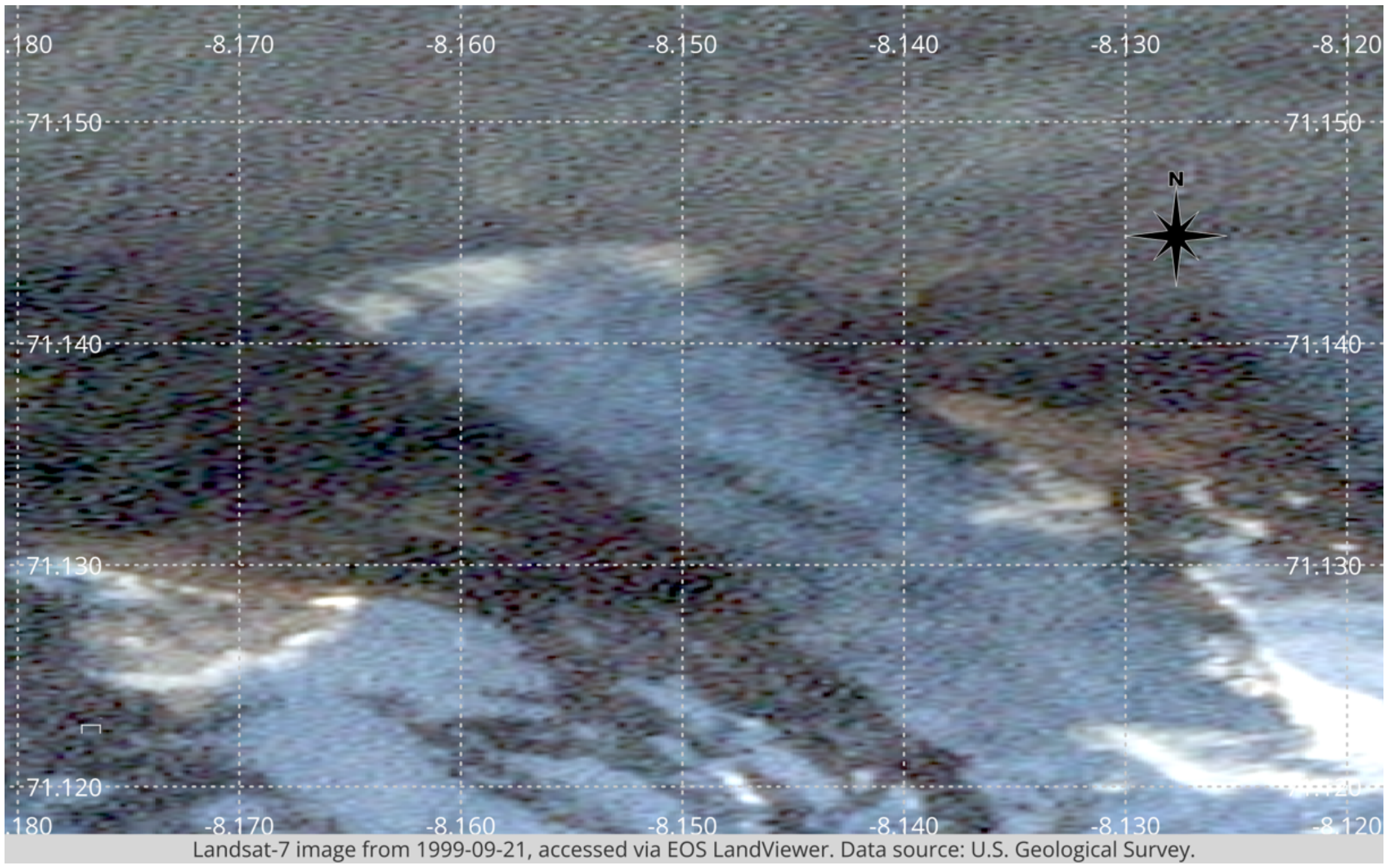

Figure S44 – Pre- 2000 Mw 6.0 earthquake Landsat-7 satellite image of Kjerulf Glacier from 1999-09-21. Image courtesy of U.S. Geological Survey, accessed via EOSDA LandViewer (https://eos.com/landviewer).

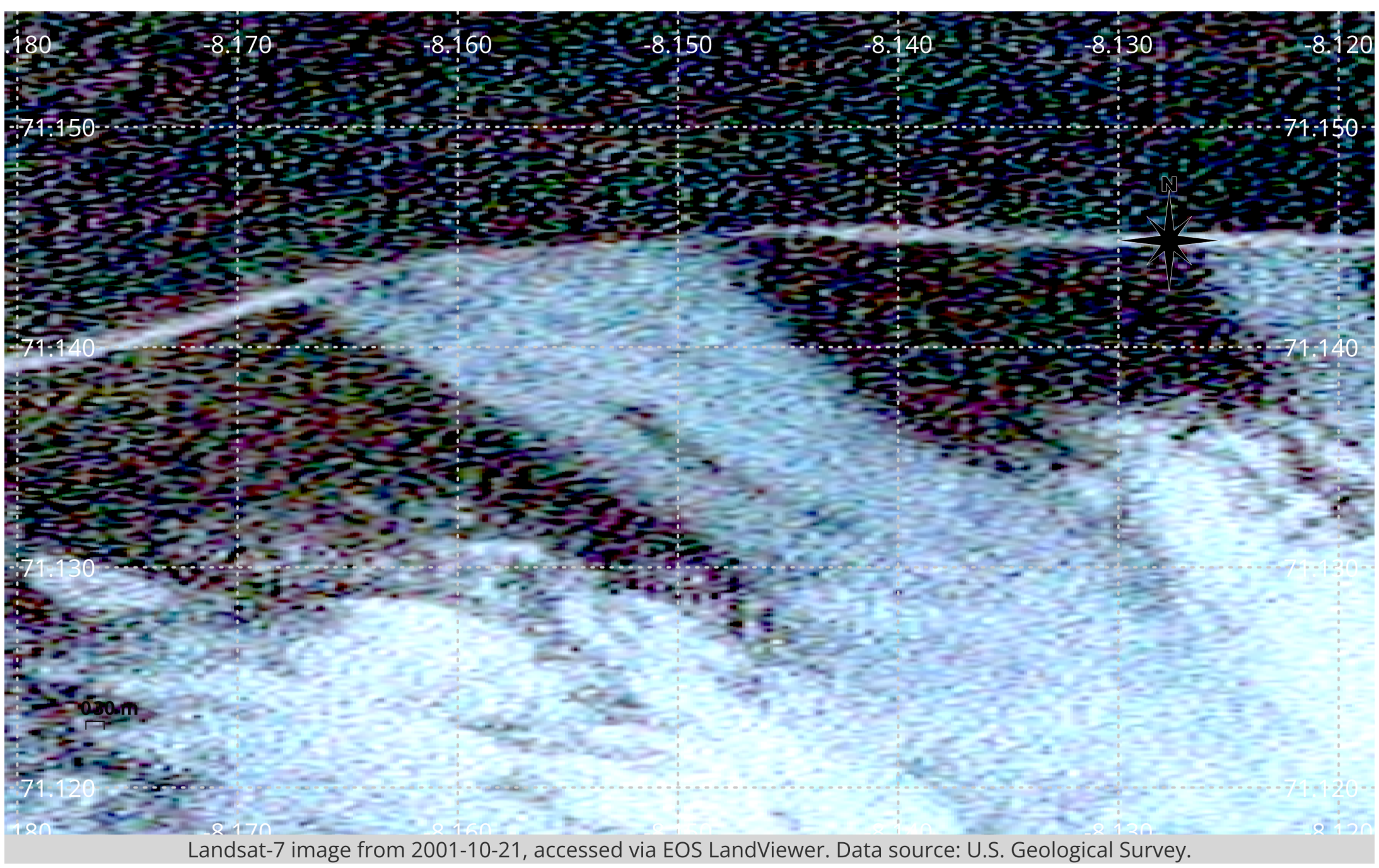

Figure S45 – Post- 2000 Mw 6.0 earthquake Landsat-7 satellite image of Kjerulf Glacier from 2001-10-21. Image courtesy of U.S. Geological Survey, accessed via EOSDA LandViewer (https://eos.com/landviewer).

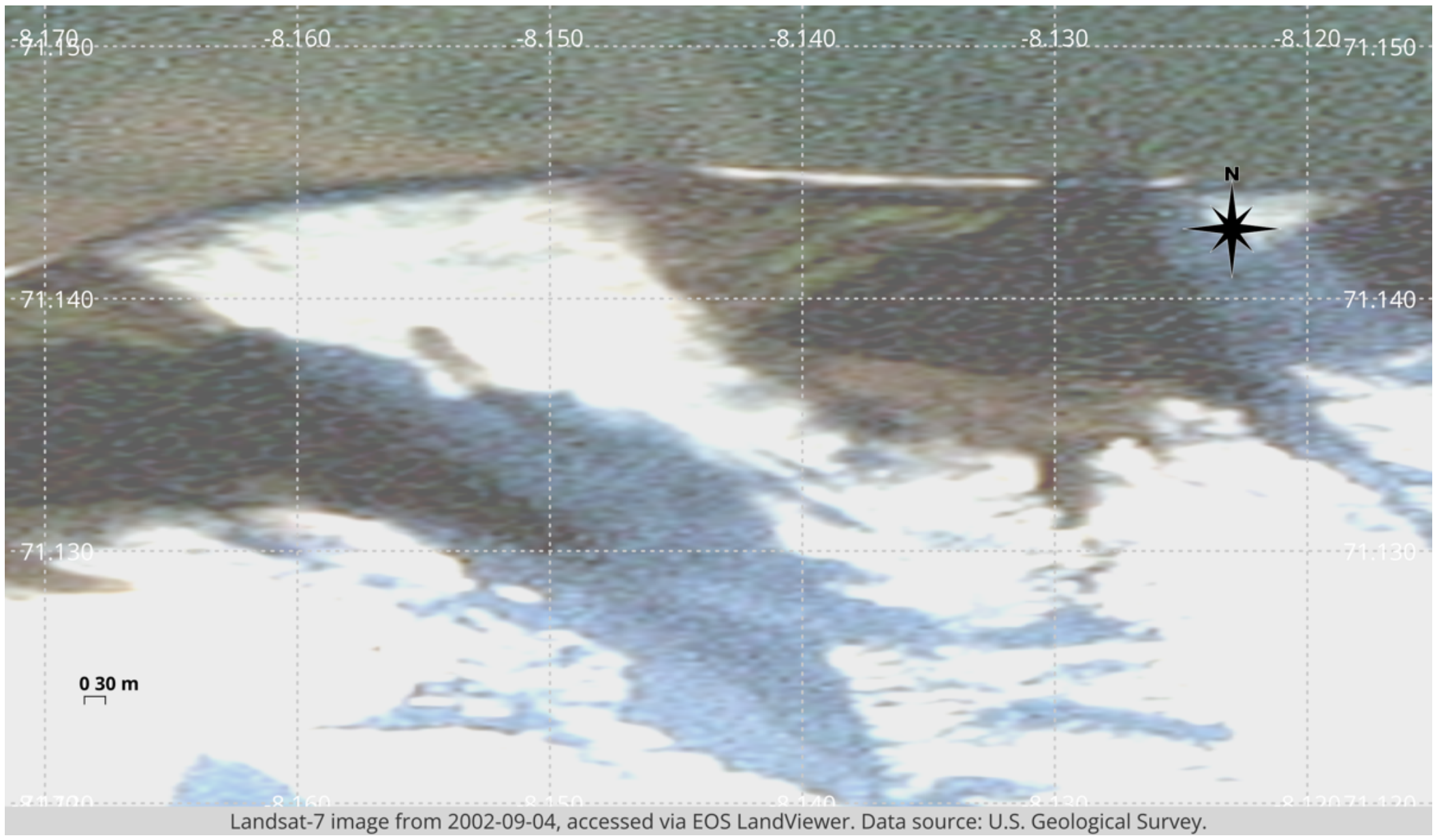

Figure S46 – Pre- 2004 Mw 5.9 earthquake Landsat-7 satellite image of Kjerulf Glacier from 2002-09-04. Image courtesy of U.S. Geological Survey, accessed via EOSDA LandViewer (https://eos.com/landviewer).

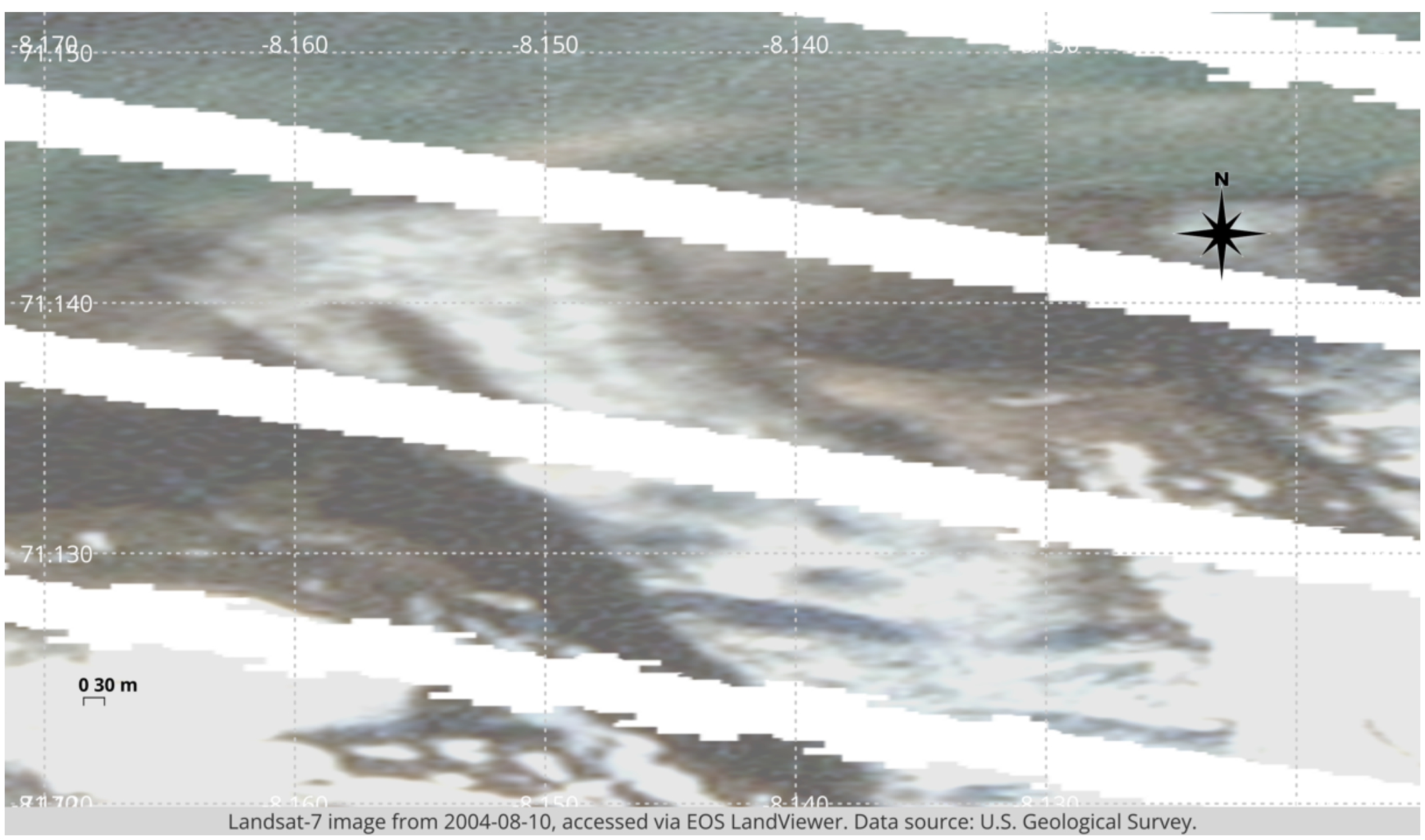

Figure S47 – Post- 2004 Mw 5.9 earthquake Landsat-7 satellite image of Kjerulf Glacier from 2004-08-10. Image courtesy of U.S. Geological Survey, accessed via EOSDA LandViewer (https://eos.com/landviewer).

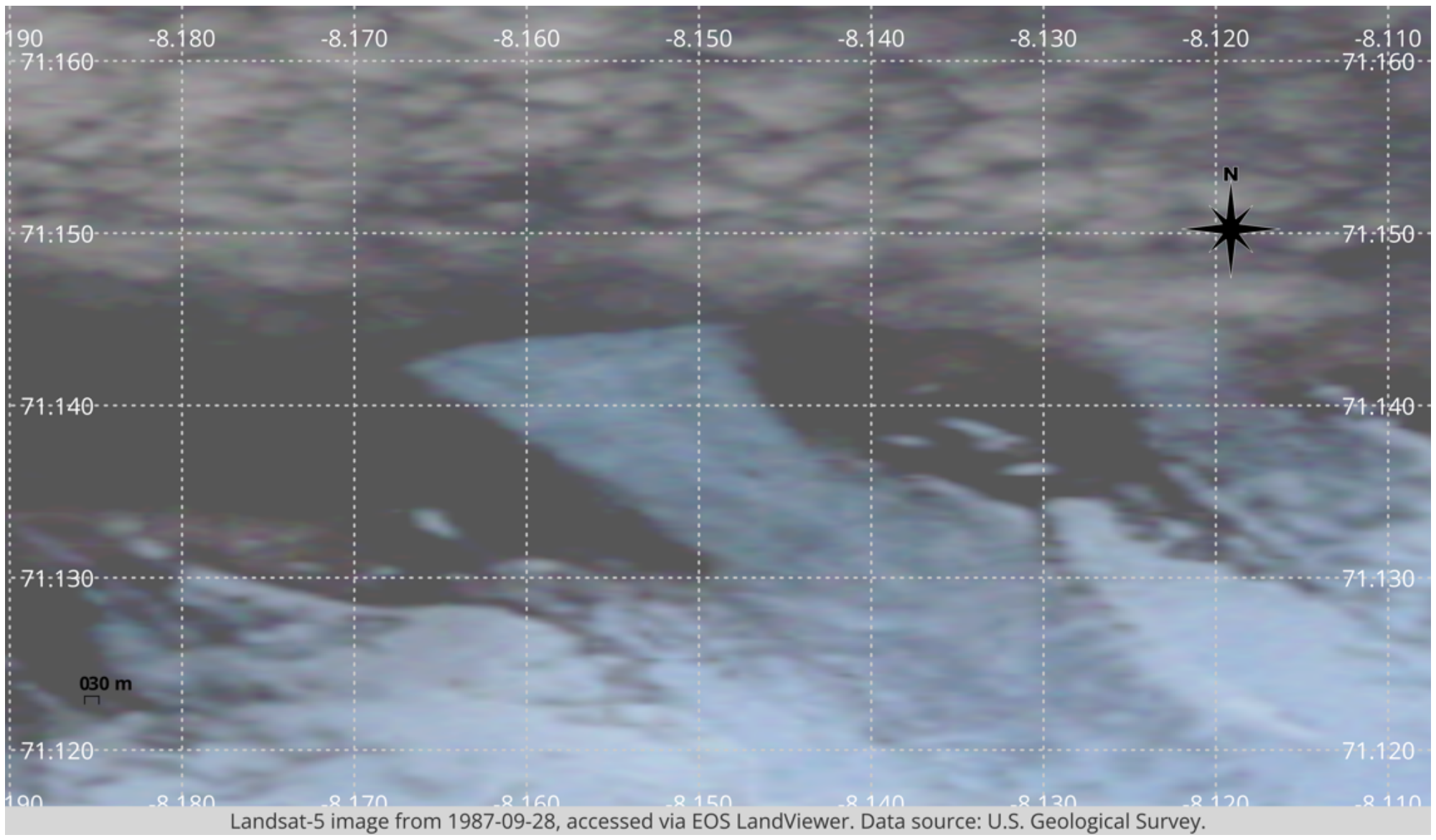

Figure S48 – Pre- 1988 Mw 6.0 earthquake Landsat-5 satellite image of Kjerulf Glacier from 1987-09-28. Image courtesy of U.S. Geological Survey, accessed via EOSDA LandViewer (https://eos.com/landviewer).

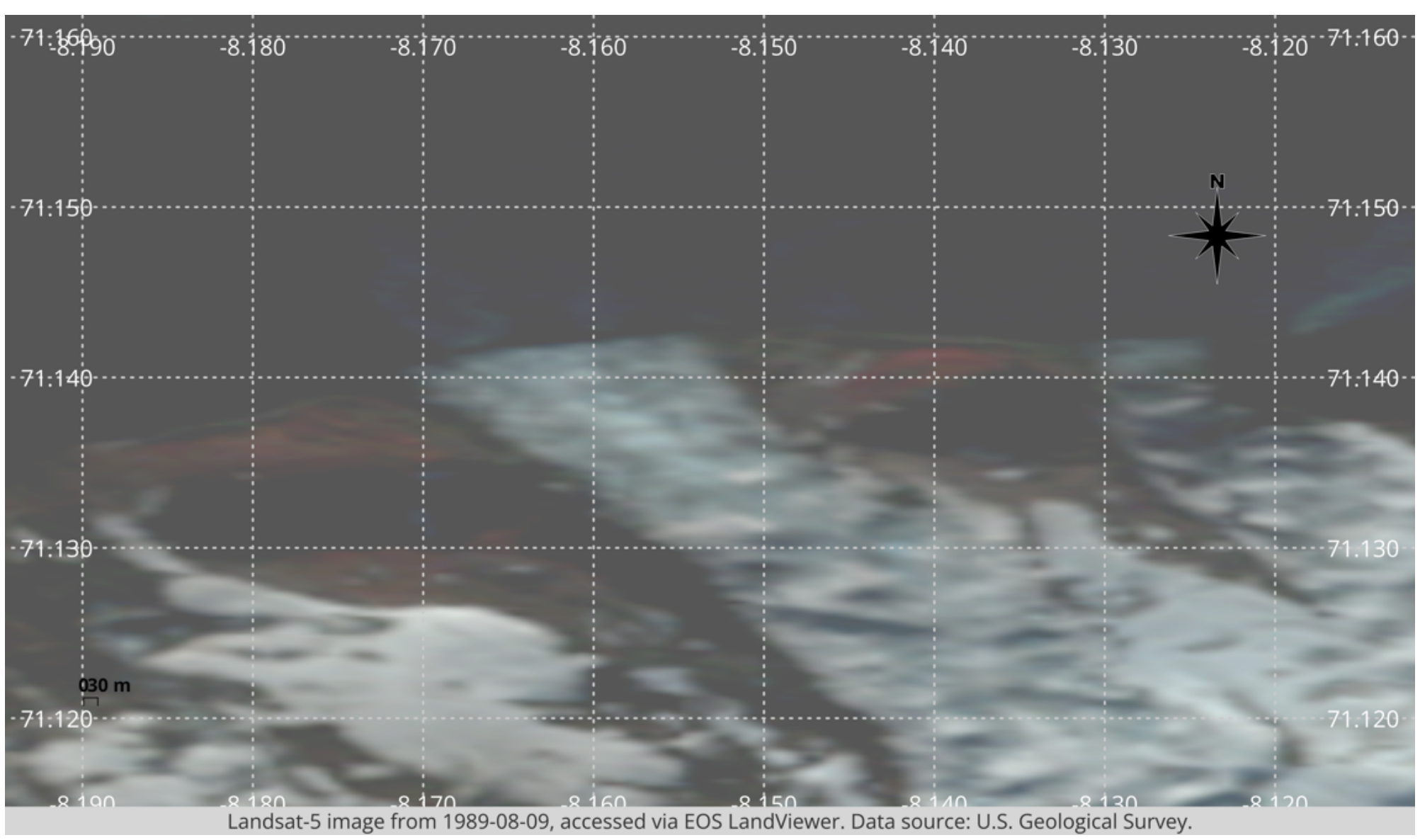

Figure S49 – Post- 1988 Mw 6.0 earthquake Landsat-5 satellite image of Kjerulf Glacier from 1989-08-09. Image courtesy of U.S. Geological Survey, accessed via EOSDA LandViewer (https://eos.com/landviewer).

Table S1 – Comparison of the relocated epicenter the focal depth using both local stations and waveform model, with the USGS (https://earthquake.usgs.gov/), GFZ (https://geofon.gfz.de/), IPGP (http://geoscope.ipgp.fr/index.php/en/catalog), and GCMT (https://www.globalcmt.org/) solutions.

Network	Focal depth, km	Latitude, º	Longitude, º
Relocated from local stations	18	71.1935	-8.1905
Waveform model	16		
USGS	13.5	71.1760	-8.1730
GFZ	18	71.1200	-8.0100
IPGP (fixed USGS)	15	71.1760	-8.1730
GCMT	19.2	71.0800	-7.7500

Table S2 – 1D velocity model extracted on CRUST1.0 package [41] used in waveform modeling.

Depth (beneath 2.7 water layer)	Vp (km/s)	Vs (km/s)	Rho (g/cm**3)
0.00	2.08	0.65	1.96
0.70	5.00	2.70	2.55
1.70	6.50	3.70	2.85
3.90	7.10	4.05	3.05
10.70	8.08	4.49	3.33

Table S3 – Geometric characteristics of the 2025 Kjerulf Glacier rock avalanche derived from MAXAR imagery and the pre-failure 2-m ArcticDEM. Runout distances were measured both as straight line path, and as a topography-following (curvilinear) path. Because the distal part of the avalanche may have extended into the ocean, the reported runout distances and corresponding H/L ratios represent minimum estimates.

Parameter	Value
Debris deposit area:	0.9 km^2
Rock volume	0.81 – 1.17x10^6 m^3
Height (H):	803 m
Length straight (L_str):	2159 m
Length curvilinear (L_curv):	2265 m
H/L_str:	0.37
H/L_curv:	0.35
Reach angle (L_str):	20.4°
Reach angle (L_curv):	19.5°

Table S4 – SLBL-modelled basal surfaces with their tolerance (*C*) value, calculated source area volume, maximum and average source area thickness, and an interpretation of their geological plausibility. Geologically feasible surfaces, based on comparison with the expected failure morphology and surrounding topography, were used to define the likely rock avalanche volume range.

Name	Volume (1 x 10^6 m^3)	Volume (m^3)	Max. Thickness (m)	Avg. Thickness (m)	Tolerance (*C*)	Geol. Plausible
slbl_auto	1,0022	1 002 230	94,05	36,67	-0,02817	Yes
slbl_c_0_035	1,1654	1 165 360	104,94	42,64	-0,0350	Yes
slbl_c_0_040	1,2849	1 284 940	112,92	47,01	-0,0400	No
slbl_c_0_045	1,4046	1 404 600	120,90	51,39	-0,0450	No
slbl_c_0_025	0,9265	926 536	88,99	33,90	-0,0250	Yes
slbl_c_0_020	0,8074	807 385	81,02	29,54	-0,0200	Yes
slbl_c_0_015	0,6889	688 884	73,07	25,20	-0,0150	No

Table S5 – USGS date, origin time, and epicenter location of the four earthquakes occurred close to Kjerulf in 1988, 1997, 2000, and 2004; and our estimations of the relatively relocated for 1997, 2000, and 2004 earthquakes.

Date (YYYY-MM-DD)	Time, UTC (USGS)	Lat.º (USGS)	Long.º (USGS)	Lat.º relocated	Long.º relocated
1988-12-13	4:01:38	71.1340	-7.6340	------------	------------
1997-12-13	7:02:03	71.2540	-8.3290	71.1830	-8.3201
2000-05-21	19:58:47	71.1900	-8.2630	71.1771	-8.2542
2004-04-14	23:07:39	71.0670	-7.7470	71.1383	-7.9013

References

1. Cesca, S., Letort, J., Razafindrakoto, H. N., Heimann, S., Rivalta, E., Isken, M. P., ... & Dahm, T. (2020). Drainage of a deep magma reservoir near Mayotte inferred from seismicity and deformation. *Nature geoscience*, *13*(1), 87-93. https://doi.org/10.1038/s41561-019-0505-5
2. Cesca, S., Sugan, M., Rudzinski, Ł., Vajedian, S., Niemz, P., Plank, S., ... & Dahm, T. (2022). Massive earthquake swarm driven by magmatic intrusion at the Bransfield Strait, Antarctica. *Communications Earth & Environment*, *3*(1), 89. https://doi.org/10.1038/s43247-022-00418-5
3. Havskov, J., & Ottemoller, L. (2010). *Routine data processing in earthquake seismology: with sample data, exercises and software*. Springer Science & Business Media. https://doi.org/10.1007/978-90-481-8697-6
4. Zahradník, J., & Sokos, E. (2018). ISOLA code for multiple-point source modeling. In *Moment tensor solutions: A useful tool for seismotectonics* (pp. 1-28). Cham: Springer International Publishing. https://doi.org/10.1007/978-3-319-77359-9_1
5. Gibbons, S. J., Harris, D. B., Dahl-Jensen, T., Kværna, T., Larsen, T. B., Paulsen, B., & Voss, P. H. (2017). Locating seismicity on the Arctic plate boundary using multiple-event techniques and empirical signal processing. *Geophysical Journal International*, *211*(3), 1613-1627. https://doi.org/10.1093/gji/ggx398
6. Jeddi, Z., Ottemöller, L., Sørensen, M. B., Rezaei, S., Gibbons, S. J., Strømme, M. L., ... & Dahl-Jensen, T. (2021). Improved seismic monitoring with OBS deployment in the Arctic: A pilot study from offshore western Svalbard. *Seismological Society of America*, *92*(5), 2705-2717. https://doi.org/10.1785/0220200471
7. NNSN (2025). Norwegian National Seismic Network (NNSN) [Dataset]. University of Bergen, https://nnsn.geo.uib.no/ (accessed 2025).
8. Gomes, J. R. A., & do Nascimento, A. F. (2021). Monitoring seismic velocity changes in the Saint Peter and Saint Paul Archipelago-Equatorial Atlantic using ambient noise

interferometry. *Journal of South American Earth Sciences*, *109*, 103251. https://doi.org/10.1016/j.jsames.2021.103251
9. de Queiroz, D. É., do Nascimento, A. F., & Schimmel, M. (2017). Microseismic noise in the Saint Peter and Saint Paul Archipelago, equatorial Atlantic. *Journal of South American Earth Sciences*, *80*, 304-315. https://doi.org/10.1016/j.jsames.2017.09.035
10. Claerbout, J. F. (1968). Synthesis of a layered medium from its acoustic transmission response. *Geophysics*, *33*(2), 264-269. https://doi.org/10.1190/1.1439927
11. Rickett, J., & Claerbout, J. (1996). Passive seismic imaging applied to synthetic data. *Stanford Exploration Project*, *92*, 83-90.
12. Illien, L., Turowski, J. M., Sens-Schönfelder, C., Berenfeld, C., & Hovius, N. (2025). Predictable recovery rates in near-surface materials after earthquake damage. *Nature communications*, *16*(1), 1790. https://doi.org/10.1038/s41467-025-57151-8
13. D'hour, V., Schimmel, M., Do Nascimento, A. F., Ferreira, J. M., & Lima Neto, H. C. (2016). Detection of subtle hydromechanical medium changes caused by a small-magnitude earthquake swarm in NE Brazil. *Pure and Applied Geophysics*, *173*(4), 1097-1113. https://doi.org/10.1007/s00024-015-1156-0
14. Dietze, M. (2018). eseis: Environmental seismology toolbox for R [Software]. GitHub, https://github.com/coffeemuggler/eseis.
15. Wegler, U., & Sens-Schönfelder, C. (2007). Fault zone monitoring with passive image interferometry. *Geophysical Journal International*, *168*(3), 1029-1033. https://doi.org/10.1111/j.1365-246X.2006.03284.x
16. Brenguier, F., Campillo, M., Hadziioannou, C., Shapiro, N. M., Nadeau, R. M., & Larose, É. (2008). Postseismic relaxation along the San Andreas fault at Parkfield from continuous seismological observations. *science*, *321*(5895), 1478-1481. https://doi.org/10.1126/science.1160943
17. Hobiger, M., Wegler, U., Shiomi, K., & Nakahara, H. (2014). Single-station cross-correlation analysis of ambient seismic noise: application to stations in the surroundings of the 2008 Iwate-Miyagi Nairiku earthquake. *Geophysical Journal International*, *198*(1), 90-109. https://doi.org/10.1093/gji/ggu115
18. Ji, D., Zhao, H., Cheng, H., Yang, X., & Ge, L. (2022). Damage evolution and fracture behavior of different materials specimens containing a central hole subjected to local loading. *Scientific Reports*, *12*(1), 16560. https://doi.org/10.1038/s41598-022-21020-x
19. Sens-Schönfelder, C., & Wegler, U. (2006). Passive image interferometry and seasonal variations of seismic velocities at Merapi Volcano, Indonesia. *Geophysical Research Letters*, *33*(21), 1-5. https://doi.org/10.1029/2004jb003011
20. Guillemot, A., van Herwijnen, A., Larose, E., Mayer, S., & Baillet, L. (2021). Effect of snowfall on changes in relative seismic velocity measured by ambient noise correlation. *The Cryosphere*, *15*(12), 5805-5817. https://doi.org/10.5194/tc-15-5805-2021
21. Pagani, M., Monelli, D., Weatherill, G., Danciu, L., Crowley, H., Silva, V., Henshaw, P., Butler, L., Nastasi, M., Panzeri, L., Simionato, M., & Viganò, D. (2014). OpenQuake

Engine: An open hazard (and risk) software for the Global Earthquake Model. *Seismological Research Letters*, 85(3), 692–702. https://doi.org/10.1785/0220130087

22. de Melo, G. W.S., Grevemeyer, I., Lange, D., Metz, D., & Kopp, H. (2025). Relationship between rupture length and magnitude of oceanic transform fault earthquakes. *Geophysical Research Letters*, *52*(1), e2024GL112891. https://doi.org/10.1029/2024GL112891
23. Kotha, S.R., Weatherill, G., Bindi, D. *et al.* A regionally-adaptable ground-motion model for shallow crustal earthquakes in Europe. *Bull Earthquake Eng* **18**, 4091–4125 (2020). https://doi.org/10.1007/s10518-020-00869-1
24. Weatherill, G., Kotha, S.R. & Cotton, F. A regionally-adaptable "scaled backbone" ground motion logic tree for shallow seismicity in Europe: application to the 2020 European seismic hazard model. *Bull Earthquake Eng* **18**, 5087–5117 (2020). https://doi.org/10.1007/s10518-020-00899-9
25. Atkinson, G. M. (2010). Ground-motion prediction equations for Hawaii from a referenced empirical approach. *Bulletin of the Seismological Society of America*, *100*(2), 751-761. https://doi.org/10.1785/0120090098
26. Maufroy, E., Cruz-Atienza, V. M., Cotton, F., & Gaffet, S. (2015). Frequency-scaled curvature as a proxy for topographic site-effect amplification and ground-motion variability. *Bulletin of the seismological society of America*, *105*(1), 354-367. https://doi.org/10.1785/0120140089
27. Maxar Technologies (2025). WorldView-2 satellite imagery, Jan Mayen Island, acquired 14 June 2024, 16 May 2025, and 14 July 2025 [Dataset]. Copyright Maxar Technologies.
28. QGIS.org (2025). QGIS Geographic Information System. QGIS Association. http://www.qgis.org
29. Scheffler, D., Hollstein, A., Diedrich, H., Segl, K., & Hostert, P. (2017). AROSICS: An automated and robust open-source image co-registration software for multi-sensor satellite data. *Remote sensing*, *9*(7), 676. https://doi.org/10.3390/rs9070676
30. Jaboyedoff, M., Carrea, D., Derron, M. H., Oppikofer, T., Penna, I. M., & Rudaz, B. (2020). A review of methods used to estimate initial landslide failure surface depths and volumes. *Engineering Geology*, *267*, 105478. https://doi.org/10.1016/j.enggeo.2020.105478
31. Hermanns, R. L., Niedermann, S., Villanueva, G. A., & Schellenberger, A. (2006). Rock avalanching in the NW Argentine Andes as a result of complex interactions of lithologic, structural and topographic boundary conditions, climate change and active tectonics. In *Landslides from Massive Rock Slope Failure:[proceedings of the NATO Advanced Research Workshop on Massive Rock Slope Failure-New Models for Hazard Assessment, Celano, Italy, 16-21 June 2002]* (pp. 497-520). Springer.
32. Hilger, P., Hermanns, R. L., Gosse, J. C., Jacobs, B., Etzelmüller, B., & Krautblatter, M. (2018). Multiple rock-slope failures from Mannen in Romsdal Valley, western Norway, revealed from Quaternary geological mapping and 10Be exposure dating. *The Holocene*, *28*(12), 1841-1854.

33. Svennevig, K., Hermanns, R. L., Keiding, M., Binder, D., Citterio, M., Dahl-Jensen, T., ... & Voss, P. H. (2022). A large frozen debris avalanche entraining warming permafrost ground—the June 2021 Assapaat landslide, West Greenland: Svennevig et al. *Landslides*, *19*(11), 2549-2567.
34. Hungr, O., Leroueil, S., & Picarelli, L. (2014). The Varnes classification of landslide types, an update. *Landslides*, *11*(2), 167-194.
35. Sandwell, D. ., R. . Mellors, X. Tong, M. Wei, and P. Wessel (2011), Open radar interferometry software for mapping surface deformation, Eos Trans. AGU, 92(28), doi:10.1029/2011EO280002.
36. McGuire, J. J. (2008). Seismic cycles and earthquake predictability on East Pacific Rise transform faults. *Bulletin of the Seismological Society of America*, *98*(3), 1067-1084. https://doi.org/10.1785/0120070154
37. Nishimura, C. E., & Forsyth, D. W. (1988). Rayleigh wave phase velocities in the Pacific with implications for azimuthal anisotropy and lateral heterogeneities. *Geophysical Journal International*, *94*(3), 479-501. https://doi.org/10.1111/j.1365-246X.1988.tb02270.x
38. Shearer, P. M. (1997). Improving local earthquake locations using the L1 norm and waveform cross correlation: Application to the Whittier Narrows, California, aftershock sequence. *Journal of Geophysical Research: Solid Earth*, *102*(B4), 8269-8283. https://doi.org/10.1029/96JB03228
39. United States Geological Survey (2004). *Landsat-5 TM and Landsat-7 ETM+ satellite imagery of Jan Mayen Island, acquired 15 July 1998, 10 June 2000, 3 August 2001, 28 July 2002, 12 September 2003, and 22 July 2004* [Dataset]. Accessed via EOSDA LandViewer (https://eos.com/landviewer/). Copyright U.S. Geological Survey.
40. Cesca, S., Heimann, S., & Dahm, T. (2011). Rapid directivity detection by azimuthal amplitude spectra inversion. *Journal of seismology*, *15*(1), 147-164.
41. Laske, G., Masters, G., Ma, Z., & Pasyanos, M. (2013, April). Update on CRUST1. 0—A 1-degree global model of Earth's crust. In *Geophysical research abstracts* (Vol. 15, No. 15, p. 2658).
